# Computational screening of oncogenic genetic variations in tumor suppressor proteins driving gastric cancer pathogenesis

**DOI:** 10.1371/journal.pone.0358440

**Published:** 2026-09-17

**Authors:** Faria Ferdouse Mim, Taslima Akter Sumiya, Roksana Khanam, Jannati Akter, Farhana Arzu, Samia Haque, Md. Roman Miah, K.M. Tanjida Islam, Shahin Mahmud

**Affiliations:** 1 Department of Biotechnology and Genetic Engineering, Mawlana Bhashani Science and Technology University, Santosh, Tangail-1902, Bangladesh; 2 Department of Clinical Pharmacy and Pharmacology, University of Dhaka, Dhaka, Bangladesh; The University of Texas Rio Grande Valley, UNITED STATES OF AMERICA

## Abstract

Gastric cancer (GC) is currently the fifth most common cancer globally, often driven by dysregulation of tumor suppressor pathways. While individual studies on genetic variations of proteins are common, a comprehensive systems-level analysis of proteins regulating GC pathways showing both expression dysregulation and high mutation frequency remains unexplored. Therefore, our study aimed to identify critical genes and their pathogenic variations disrupting the tumor-suppressive capacity of the GC pathway. We employed a deep learning-based graph neural network model to identify genes exhibiting both dysregulated expression and high mutation propensity in GC. Key tumor suppressor proteins (TP53, CDH1, and APC), their genetic variants, and molecular components were subjected to in-depth computational analyses, including evolutionary conservation profiling, biophysical energetics assessment, and unsupervised machine learning for conformational change detection. Variants were validated from the cBioPortal database and patient survival data. Our deep learning model demonstrated exceptional performance (MSE: 0.00482 ± 0.00023 to 0.07108 ± 0.00437; R²: 0.85098 ± 0.01903 to 0.85899 ± 0.01987; AUC-ROC: 0.93095 ± 0.01758 to 0.93309 ± 0.00725) and identified 1,886 genes exhibiting both differential expression and mutation propensity. Graph neural network analysis revealed TP53 as the most prominent hub gene (47.6% mutation frequency), followed by ERBB2 (8.8%), CDH1 (8.2%), and APC (6.8%). Variants validation from cBioPortal confirmed the association of GC with 36 missense SNPs that critically affect post-translational modification (methylation and phosphorylation) sites and 60 nonsense SNPs. Furthermore, TP53, CDH1, and APC were significantly upregulated in GC tissues and associated with altered patient survival (p < 0.05). The transcription factor EZH2 and miRNA miR-129-5p were identified as key regulatory elements affecting all three tumor suppressors. Additionally, mutations trigger dysregulation of multiple common oncogenes, including CCNE1/2 and FGFR2. This systems-level analysis provides a molecular framework demonstrating how pathogenic variants fundamentally compromise tumor suppressor proteins in GC pathways, leading to the identification of potential biomarkers and precise therapeutic decisions for GC intervention.

## 1. Introduction

Gastric cancer (GC) is recognized as the primary epithelial malignancy of the stomach, characterized by its complexity and diversity [1–3]. Although there has been a general decline in both incidence and mortality rates in many countries over the past few decades, GC remains the fifth most common cancer and the fourth leading cause of cancer-related deaths globally [4,5].

GC involves dysregulation of several key signaling pathways that contribute to its development, progression, metastasis, and therapeutic resistance, including receptor tyrosine kinases (RTKs) such as EGFR, HER2, VEGFR, and c-MET, which activate downstream pathways like MAPK/ERK and PI3K/AKT/mTOR, promoting cell growth, proliferation, survival, and angiogenesis [6,7]. The p53 pathway, crucial for DNA repair, cell cycle control, and apoptosis, is often disrupted in GC, while the Wnt/β-catenin pathway plays a role in cell proliferation and migration. Additionally, the NF-κB and TGF-β pathways are involved in inflammation and immune response [8].

In general, tumor suppressor proteins act as guardians of the genome, regulating vital cellular processes like cell cycle control, apoptosis, and DNA damage repair [9]. Genetic variations in tumor suppressor genes, such as *TP53*, *CDH1*, and *APC,* are closely associated with GC pathways and extensively reported in GC patients [10–14]. Tumor suppressor proteins, which normally regulate cell growth and prevent tumor formation, are mutation-prone in most of the cancers, including GC. Pathogenic genetic variations in these proteins can disrupt their normal function, leading to uncontrolled cell growth and tumor development [15,16].

Among genetic variations, frameshift, missense, nonsense, splice site, ncRNA, and UTR (untranslated region) variations are most frequent and predominantly found in humans. Generally, frameshift mutations disrupt the reading frame, missense mutations alter amino acids, nonsense mutations introduce premature stop codons, splice site mutations affect RNA splicing, mutations in ncRNAs impact gene regulation, and UTR mutations influence mRNA stability and translation, all potentially leading to altered or non-functional proteins and various phenotypic consequences, including cancers [17,18].

However, the impact of missense SNPs (Single-nucleotide polymorphisms) on protein function, stability, and disease development is not always straightforward to predict, like other genetic variations. This complexity arises from the intricate interplay between the altered amino acid, its location within the protein structure, its role in the protein’s overall function, and molecular interference by TFs (transcription factors), miRNAs, methylation, phosphorylation, ubiquitylation sites, etc. [19].

Missense SNPs in tumor suppressor proteins can alter protein structure, stability, and function, thereby disrupting their ability to suppress tumorigenesis [20]. In addition, missense SNPs can affect tumor suppressor proteins through multiple mechanisms, including altering protein folding and stability, disrupting critical functional domains, and regulating transcriptional, post-transcriptional, and post-translational modifications [21,22].

Evaluating the impact of clinically significant missense SNPs in tumor suppressor proteins and understanding the functional consequences and molecular interferences of missense SNPs is essential for unraveling the complex genetic landscape of GC. Furthermore, these structural and functional changes as a result of the pathogenic genetic variations can impair protein-protein interactions, disrupt signaling pathways, and ultimately impair the tumor suppressor activity, rendering cells more susceptible to malignant transformation and ultimately contributing to the development and progression of cancer.

Therefore, elucidating the impact and molecular mechanism of clinically significant pathogenic variations in tumor suppressor proteins TP53, APC, and CDH1 of the GC pathway is crucial for improving our understanding of the genetic factors underlying GC risk and pathogenesis. This knowledge can guide the development of personalized diagnostic and therapeutic strategies, ultimately enhancing patient outcomes.

**Fig 1:**
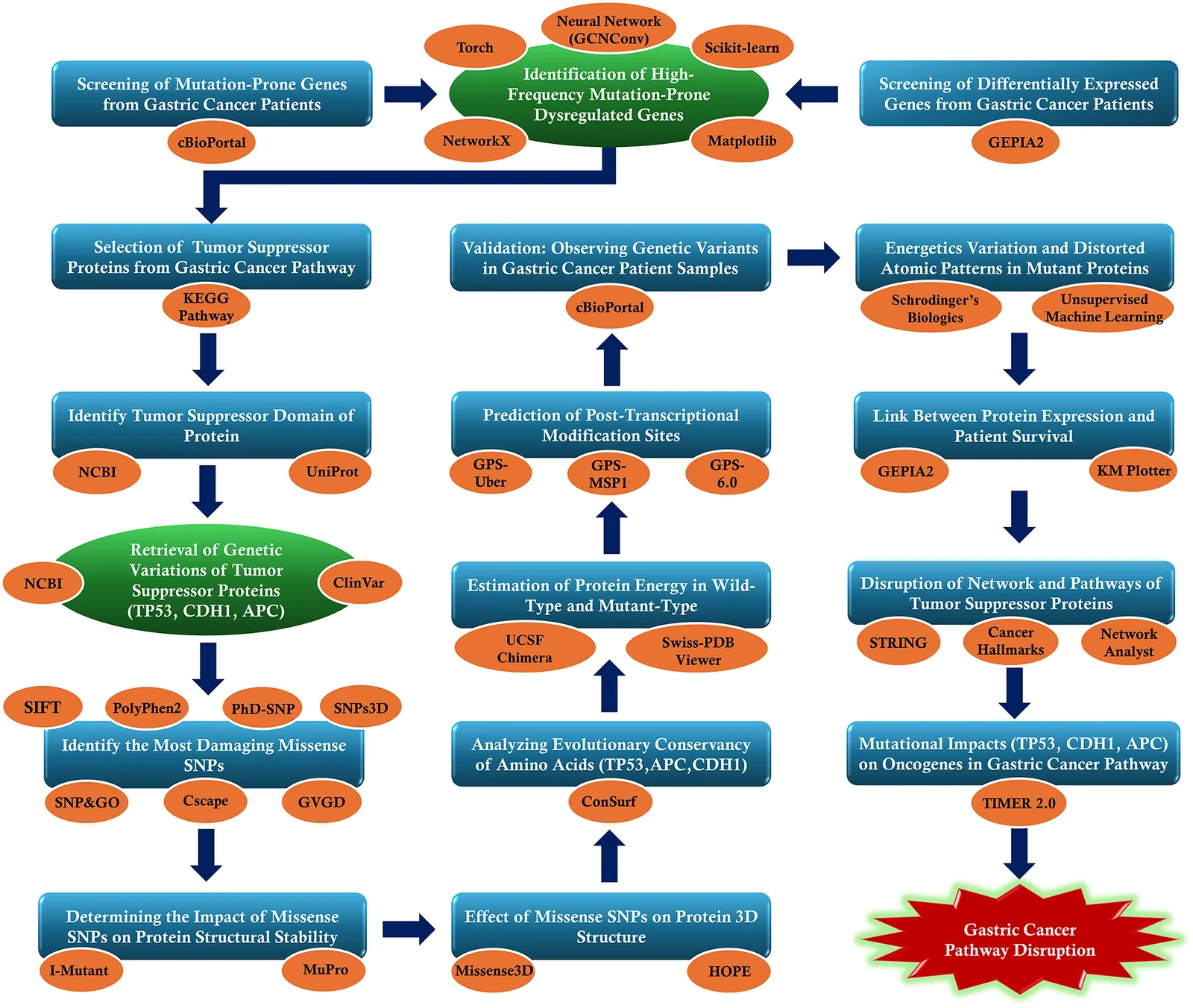
The sequential flow diagram of the study to establish a link between differentially expressed high-frequency mutation-prone genes, pathogenic genetic variations, and GC pathogenesis.

While previous studies have explored the molecular basis of early diagnostic and prognostic biomarkers for gastric cancer [23] and the effects of missense SNPs on individual tumor suppressor proteins TP53, APC, and CDH1 [24–26], comprehensive analysis to identify key proteins that are highly dysregulated and have high mutation frequency in GC at the systems level is lacking. Additionally, most of the previous studies focus on the identification of an isolated type of pathogenic variation, whereas our study focuses on the identification and validation of the direct impact of all clinically significant pathogenic genetic variations on key proteins that disrupt the balance of the tumor suppressor capability of the GC pathway. Our study also focused on identifying genetic components crucial to disrupting this balance, contributing to aiding this disruption, and corroborating the development of GC, unveiling the molecular mechanisms of genetic variations in GC pathogenesis. The overview of this study is presented in Fig 1.

## 2. Methodology

### 2.1. Screening of differentially expressed mutation-prone genes in GC

We developed a deep learning prioritization and ranking Graph Neural Networks (GNNs) model, specifically GCNConv (Graph Convolutional Networks), to identify GC pathway-specific differentially expressed mutation-prone genes. At first, RNAseq gene expression data were retrieved from the GEPIA2 database (http://gepia2.cancer-pku.cn/) [27], and GC-specific mutation-prone genes were retrieved from the cBioPortal database (https://www.cbioportal.org/) [28]. The common genes between dysregulated genes and mutation-prone genes were identified using a Venn diagram from the InteractiVenn tool (https://www.interactivenn.net/) [29]. The protein-protein interaction network was constructed using the common genes from the STRING database (https://string-db.org/) [30], which contains both experimentally validated and predicted protein-protein interactions. Furthermore, GC pathway-specific genes were collected from the KEGG pathway database (https://www.kegg.jp/) [31] that contains pathways of molecular mechanisms for disease development, including cancer pathways [32]. These commonly mutation-prone differentially expressed genes (DEGs), genes from protein-protein interaction networks, and GC pathway-specific genes were utilized as input to develop a robust computational framework to identify pathway-specific, highly dysregulated, mutation-prone genes responsible for GC pathogenesis. Secondly, the NetworkX library of Python was implemented for graph construction and topological analysis (degree, betweenness, closeness) [33]. Thirdly, the PyTorch library for tensor operations and neural network architecture, and torch_geometric for graph neural network implementation, were utilized. Finally, the StandardScaler of the scikit-learn library was used for data preprocessing [34]. Specifically, we employed a Graph Convolutional Network (GCN) implemented through the GCNConv layers, which enabled the propagation of node features across the network topology [35,36]. Node features were normalized using StandardScaler to ensure consistent scaling across heterogeneous molecular data types [34]. The network architecture consisted of two GCNConv layers followed by ReLU (Rectified Linear Unit) activation functions to introduce non-linearity into the neural network, which is crucial because most of the real-world problems are non-linear, and neural networks need non-linear activation functions to learn complex patterns [37]. Subsequently, dropout regularization (rate = 0.3) was applied between layers to prevent overfitting. Model performance was rigorously assessed through train-validation-test splitting (60:20:20 ratio) using the train_test_split module from scikit-learn, where training and validation datasets were utilized for learning and internal validation purposes, whereas the testing set was utilized and masked for external validation. Moreover, a nested 5-fold cross-validation (CV) strategy was applied to maintain both hyperparameter tuning and unbiased test estimate with 2 loops: outer loop for model evaluation and inner loop for hyperparameter tuning. In this framework, the inner loop was exclusively used to optimize model hyperparameters without exposing the outer test folds, whereas the outer loop evaluated performance on completely unseen data partitions to provide an unbiased estimate of generalization performance. To mitigate information leakage, we implemented a gene-level partitioning strategy in which all features and network relationships associated with a given gene are confined to a single data split, preventing cross-set propagation of topological or annotation-derived information. Finally, the performance metrics of the model were evaluated using training and validation loss over 200 epochs.

To develop the model, the GNN is first trained to predict a propensity score based on the relevance of 3 gene categories.

(1) Mutation-prone Differentially Expressed Genes (FDR-adjusted p-values < 0.05).
(2) Genes from the protein-protein interaction network (confidence score: 0.4 (medium to balanced network).
(3) Gastric cancer (GC) pathway-specific genes.

This represents the integrated likelihood that a gene contributes to gastric cancer pathogenesis due to mutations, expression dysregulation, and network-level functional importance. A continuous propensity score is operationalized by combining normalized gene expression magnitude (Log2FC) with multiple graph-topological centrality measures (degree, betweenness, and closeness) and, when available, pathway membership information. These biological components reflect the premise that genes that are both dysregulated and centrally positioned in protein interaction networks, along with being present in the GC pathway, are more likely to act as key disease drivers. The resulting composite score serves as the dependent variable in a regression framework and ranks the top genes. Therefore, the primary regression performance metrics were mean squared error (MSE), root mean squared error (RMSE), and coefficient of determination (R²).

Afterward, the classification metrics were used as a secondary interpretive analysis (AUC-ROC reporting) rather than the primary modeling objective. For this purpose, the continuous propensity scores were binarized using a biologically interpretable threshold (≥0.5 after sigmoid normalization), representing high-confidence versus low-confidence pathogenic candidates. This transformation allowed us to assess the model’s ability to discriminate high-risk oncogenic genes from background genes, resulting in identifying the top 20 hub genes based on both regression and classification metrics. Due to the complexity of several layers of input data in the model, we did not consider the Youden index or cost-sensitive approaches in this case; rather, we chose a simplified classification boundary threshold (≥0.5 after sigmoid normalization) to remove background genes from more valuable genes. The regression captured fine-grained biological risk ranking, while ROC and precision-recall analyses provided the discrimination performance to remove background genes for prioritization tasks. All reported metrics were computed on independent training, validation, and test partitions to ensure robustness.

### 2.2. Identification of high-frequency mutation-prone genes

We have screened high-frequency mutation-prone genes by analyzing 147 GC patient samples from the cBioPortal database due to their impact on the dysregulation of proteins. Based on the deep learning neural network model and frequency analysis, we have found that tumor suppressor proteins are the most frequently mutated proteins. We have further examined their involvement in the GC pathway from the KEGG database (https://www.genome.jp/kegg/), which is a comprehensive resource for understanding high-level biological functions and utilities, including molecular-level information such as genes, proteins, and biochemical pathways [31]. Another crucial reason for choosing tumor suppressor proteins for single-nucleotide polymorphism analysis is that they play critical roles in inhibiting GC, and mutations in these genes are likely to have been implicated in the development and progression of GC [15,16].

### 2.3. Identification of tumor suppressor domains in proteins

The tumor suppressor domains of proteins were identified using the NCBI Conserved Domain Search Tool (https://www.ncbi.nlm.nih.gov/Structure/cdd/wrpsb.cgi) [38] and validated through the UniProt database (https://www.uniprot.org/) [39]. The NCBI Conserved Domain Search Tool was used to search for conserved domains within the target protein sequences. This allows searching against various domain databases, including the NCBI-curated Conserved Domain Database (CDD), to identify significant matches to known functional domains. The UniProt database was then used to gather additional information on the identified tumor suppressor domains responsible for tumor suppressor activity.

### 2.4. Retrieval of clinical variations of tumor suppressor proteins

Clinically significant variations were retrieved from the ClinVar database of NCBI (https://www.ncbi.nlm.nih.gov/clinvar/) to investigate their association with cancer [40]. ClinVar consists of curated and annotated clinically significant variants, such as frameshift, missense, nonsense, splice site, ncRNA, and UTR, to prioritize potentially pathogenic variations and provide evidence of a link between these variants and disease phenotypes. Only pathogenic variations were selected, and likely pathogenic clinical variations were considered only for missense SNP variation analysis, as sophisticated methodologies have been established to analyze missense variations compared to other variations, and these variants are often directly linked to functional consequences [41].

### 2.5. Identifying damaging, deleterious, and disease-causing missense SNPs

To identify potentially damaging, deleterious, disease-causing, and oncogenic missense SNPs, we employed a multi-faceted bioinformatics approach utilizing seven established prediction tools. We leverage the capabilities of PolyPhen2 (https://genetics.bwh.harvard.edu/pph2/) [42], SIFT (https://sift.bii.a-star.edu.sg/) [43], SNPs&GO (https://snps.biofold.org/snps-and-go/) [44], PhD-SNP (https://snps.biofold.org/phd-snp/) [45], SNPs3D (http://snps3d.org/) [46], GVGD (http://agvgd.hci.utah.edu/) [47], and Cscape (http://cscape.biocompute.org.uk/) [48]. Each tool was selected for its unique strengths to meet the ACMG variant classification guidelines [49], which are standard practice in SNP studies [50,51]. PolyPhen2 assessed the potential impact of amino acid substitutions on protein function using structural and comparative considerations, whereas SIFT predicted the effects of amino acid changes based on sequence homology and physical properties. SNPs&GO correlated SNPs with gene ontology to predict disease relevance, PhD-SNP evaluated the deleterious effects of mutations, SNPs3D provided structural insights into damaging SNPs, GVGD classified the likelihood of pathogenicity, and finally, Cscape evaluated the oncogenic potential of mutations. Incorporating the scores of seven computational tools improved forecast precision and ensured the stringency and accuracy of results.

### 2.6. Determining the impact of missense SNPs on protein structural stability

To determine the impact of missense SNPs on the structural stability of tumor suppressor proteins, we utilized the I-Mutant 2.0 tool [https://folding.biofold.org/i-mutant/i-mutant2.0.html] [52], which is an online support vector machine-based tool that predicts the effects of single-site mutations on protein stability from the sequence or structure of the protein. We also employed the MUPro tool [https://mupro.proteomics.ics.uci.edu/] [53] to corroborate our findings, as it is another well-established method for predicting the impact of mutations on protein stability.

### 2.7. Examining the effects of missense SNPs on protein 3D Structure

The structural effects of missense SNPs were examined using two complementary bioinformatics tools: Missense3D (https://missense3d.bc.ic.ac.uk/) [54] and HOPE server (https://www3.cmbi.umcn.nl/hope/) [55]. Missense3D, a web server developed by the Structural Bioinformatics Group at Imperial College London, predicts the structural changes introduced by an amino acid substitution by analyzing both experimental protein structures (PDB) and homology-predicted structures. HOPE is another web server hosted by the CMBI group at the University of Nijmegen that collects and combines structural information from a variety of web services and databases to produce a comprehensive report on the effects of a point mutation. The combined use of these two tools allowed for a thorough investigation of how missense SNPs might impact protein 3D structure, with the goal of better understanding the potential functional consequences of these genetic SNPs.

### 2.8. Analysis of protein evolutionary conservation

To assess the evolutionary conservation of the proteins, we employed the ConSurf server (https://consurfdb.tau.ac.il/) [56]. This analysis was undertaken to identify functionally important residues within these proteins, as highly conserved residues are often crucial for protein structure and function. The ConSurf server utilizes a phylogenetic approach, combining multiple sequence alignments with evolutionary modeling to predict the evolutionary conservation of each amino acid position within a protein structure. The conservation scores, which range from 1 (variable) to 9 (highly conserved), were then mapped onto the protein structures to identify the evolutionarily conserved regions.

### 2.9. Comparison of protein energy and minimized energy in wild-type and mutant proteins

To investigate the impact of mutations on tumor suppressor proteins, we further employed UCSF Chimera to introduce mutations into the proteins of interest [57]. Subsequently, the energy calculation and minimized energy calculation of these mutated proteins were performed using the Swiss-Pdb Viewer [58]. The selection of Swiss-Pdb Viewer was based on its intuitive interface and capabilities for energy minimization calculations and analysis of structural features such as H-bonds, angles, and interatomic distances. This process provided quantitative data on the energetic consequences of the introduced mutations, thereby elucidating their potential effects on protein stability and function. To assess the effects of minimized energies of the mutated proteins, they were compared to the energies of wild-type proteins.

### 2.10. Prediction of post‑translational modification sites

The impact of missense SNPs on post-translational modification (PTM) sites was elucidated by employing GPS-MSP 1.0 (https://msp.biocuckoo.org/) [59], GPS-6.0 (https://gps.biocuckoo.cn/) [60], and GPS-Uber (https://gpsuber.biocuckoo.cn/) [61] to predict methylation, phosphorylation, and ubiquitination sites, respectively. These tools were chosen due to their high accuracy and specificity in identifying PTM sites. GPS-MSP 1.0 integrates deep neural networks, convolutional neural networks, and penalty logistic regression to predict methylation sites. On the other hand, GPS-6.0, a group-based prediction system, employs transfer learning and machine learning algorithms to predict phosphorylation sites. Finally, GPS-Uber is a ubiquitin-protein ligase enzyme-substrate relationship prediction tool to predict ubiquitination sites. By applying these tools, we aimed to identify potential PTM sites in missense SNPs and elucidate their effects on protein function and stability.

### 2.11. Validation of GC-specific variations

Before further analysis, clinical variations were validated by establishing a link between SNPs and GC pathogenesis using the cBioPortal database (https://www.cbioportal.org/) [28]. The reason behind choosing the cBioPortal database is that it is a reliable and widely used specialized database focusing only on cancer genomics data for exploring the complex relationships between genomic alterations and cancer outcomes, and retrieving and analyzing genomic data from a large cohort of GC patients. Therefore, this analysis enables the identification of statistically significant correlations between genetic variations and GC pathogenesis [62].

### 2.12. Comparison of energetic patterns between wild-type and mutant proteins

Specific missense SNPs that were correlated with GC pathogenesis were further analyzed using titration curve analysis to elucidate mutation-induced changes in protein electrostatic properties. We utilized Schrodinger’s Biologics Package to generate titration curves, as this approach provides high-resolution insights into protein ionization behaviors across physiological conditions [63]. The analysis was conducted by calculating the net charge of proteins at pH intervals from 2 to 12. This methodology enabled quantitative comparison of wild-type and mutant protein ionization behaviors, revealing shifts in pKa values, protonation states, and isoelectric points that impact protein stability and function, ultimately providing insights into mutation-specific perturbations in protein electrostatic properties.

Furthermore, energetic pattern analysis was implemented to quantify mutation-induced biophysical perturbations affecting protein stability and function. We employed Schrodinger’s Biologics Package, which utilized sophisticated force field implementations with accurate energy minimization algorithms for a multi-parameter energetic analysis combining hydropathy calculations, potential energy distributions, and interaction energy computations [63]. To reveal crucial biophysical perturbations of mutation in hydrophobicity patterns, the energetic patterns exhibit changes in thermodynamic stability and molecular interactions, providing mechanistic insights into mutation-induced protein dysfunction.

### 2.13. Atomic-level analysis of proteins utilizing unsupervised machine learning

The atomic-level evaluation was conducted using unsupervised machine learning to detect subtle conformational changes and disrupted interaction networks in mutant proteins. This applied to atomic-level structural features to explore potential conformational patterns and provide comparative diversity between wild-type and mutant structures at the atomic level. We implemented scikit-learn’s k-means clustering algorithms in Python 3, combined with dimensionality reduction techniques, as this approach enables unbiased pattern recognition and scalable analysis of protein structural features [64]. The methodology involved preprocessing atomic coordinates through principal component analysis, followed by k-means clustering. Atomic features were encoded using a combination of spatial coordinates, atomic properties, and atomic interaction patterns to enable the identification of conserved atomic clusters critical for protein function, detection of mutation-induced perturbations in atomic interaction networks, and quantification of structural changes, providing a systematic framework for understanding protein dysfunction mechanisms.

### 2.14. Examination of protein expression and patient survival

To investigate the possible impact of missense SNPs on protein expression, we analyzed the expression profiles of proteins in GC using the GEPIA2 web server (http://gepia2.cancer-pku.cn) [27]. This is a comprehensive and interactive web application for gene expression analysis based on The Cancer Genome Atlas (TCGA) and Genotype-Tissue Expression (GTEx) data. We utilized the differential expression analysis module (Expression DIY) of GEPIA2 to compare the expression levels of the selected proteins between tumor and normal gastric tissues to assess whether missense SNPs in these genes might lead to altered protein expression patterns, which could potentially impact protein function, contribute to disease development, and compromise patient survival. The patient survival differences were analyzed using Kaplan-Meier plot analysis (https://kmplot.com/), which is suitable for addressing the correlation between genes or proteins and survival in more than 35 thousand samples from 21 tumor types [65]. The clinical covariates analysis of survival in different stages of cancer was analyzed using TIMER 3.0 (https://compbio.cn/timer3/) [66].

### 2.15. Analysis of the network and hallmarks of tumor suppressor proteins

We conducted network and pathway analysis of proteins utilizing the STRING database (https://string-db.org/) [30] and cancer hallmark database (https://cancerhallmarks.com) [67] respectively. STRING reconstructed the protein-protein interaction networks of tumor suppressor proteins, which integrates multiple sources of experimental and predicted protein interaction data, providing a comprehensive view of protein networks. On the other hand, the cancer hallmark database employed the cancer hallmarks enrichment plot to identify key cancer-relevant hallmarks associated with tumor suppressor proteins.

### 2.16. Analysis of gene regulatory networks of tumor suppressor proteins

A gene-regulatory network has been constructed to determine the key elements that might interfere with the tumor suppressor proteins of GC. NetworkAnalyst 3.0 (https://www.networkanalyst.ca/) was used to identify gene-regulatory networks (GRNs) and the intricate relationships between gene-microRNAs (miRNAs) and gene-transcription factors (TFs) [68]. To build and evaluate the GRNs, we specifically used TTRUST, a database of TF-target interactions, and miRTarBase v9.0, an extensive library of miRNA-target interactions [69]. We chose NetworkAnalyst 3.0 because it can integrate and analyze large biological networks and contains large databases like miRTarBase v9.0 and TTRUST.

### 2.17. Altered gene expression assessment of oncogenes

To investigate the impact of mutations on gene expression patterns in GC pathway oncogenes, we performed differential gene expression analysis between tumors with and without specific mutations. The analysis was conducted using the Gene Mutation module of TIMER 2.0 (http://timer.comp-genomics.org/) [70]. TIMER 2.0 was selected because it provides a robust platform for analyzing associations between gene mutations and expression patterns across multiple cancer types using TCGA data. The Gene Mutation module analyzed and visualized the effect of gene mutations on gene expression by comparing expression levels between mutated and wild-type tumors. For each gene of interest, we examined the statistical significance of expression differences between tumors harboring mutations and those without mutations to identify potential correlations between specific gene mutations and altered expression patterns

## 3. Results

### 3.1. Identification of the most significant dysregulated mutation-prone genes

**Fig 2:**
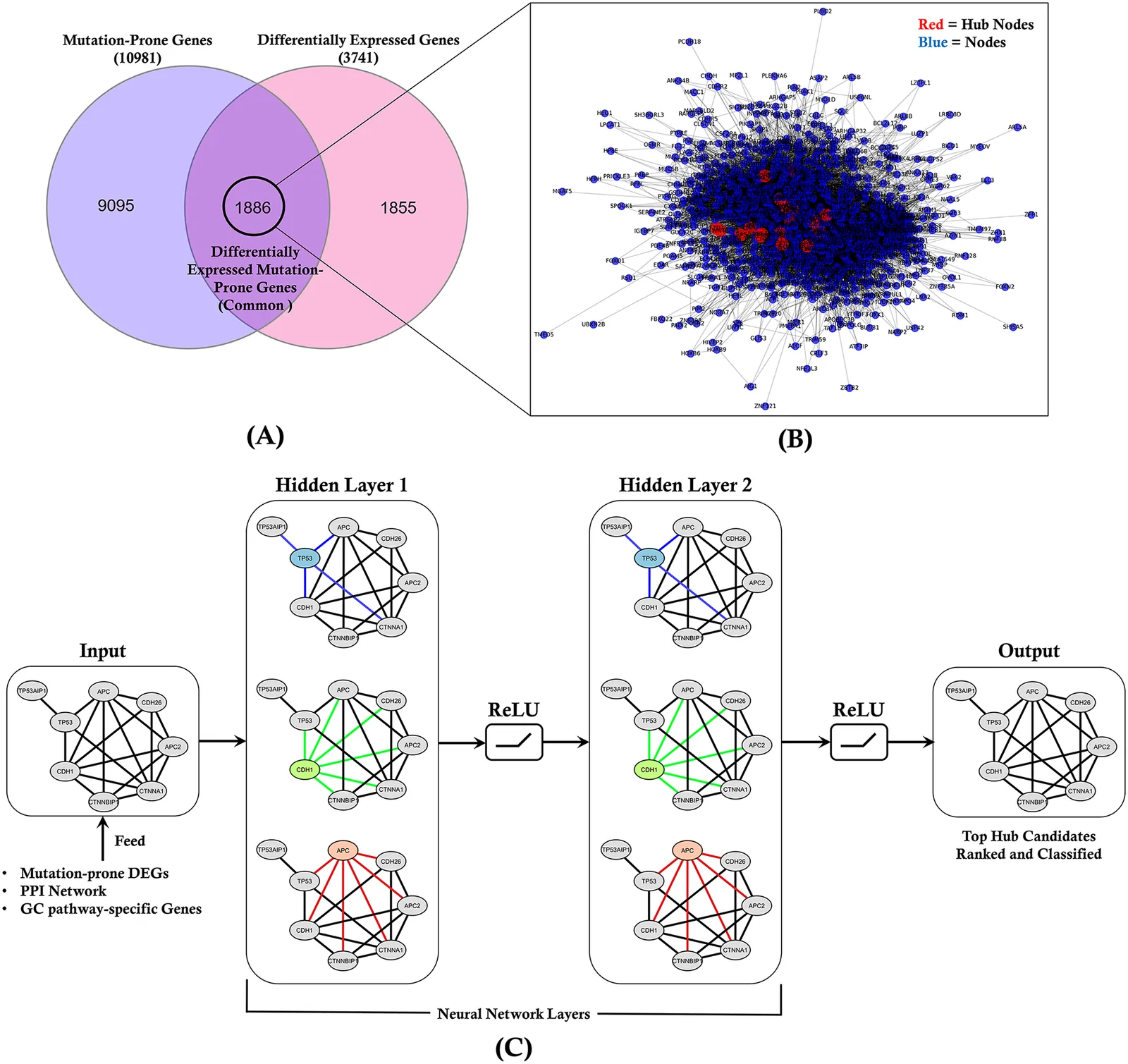
Identification of differentially expressed mutation-prone genes using the graph neural network model and evaluation of model error. **(A)** Identification of common DEGs and mutation-prone genes. **(B)** Protein-protein interaction network (Red indicates the most important GC pathway-specific genes). **(C)** GCNconv model with two hidden layers and ReLU activation.

**Fig 3:**
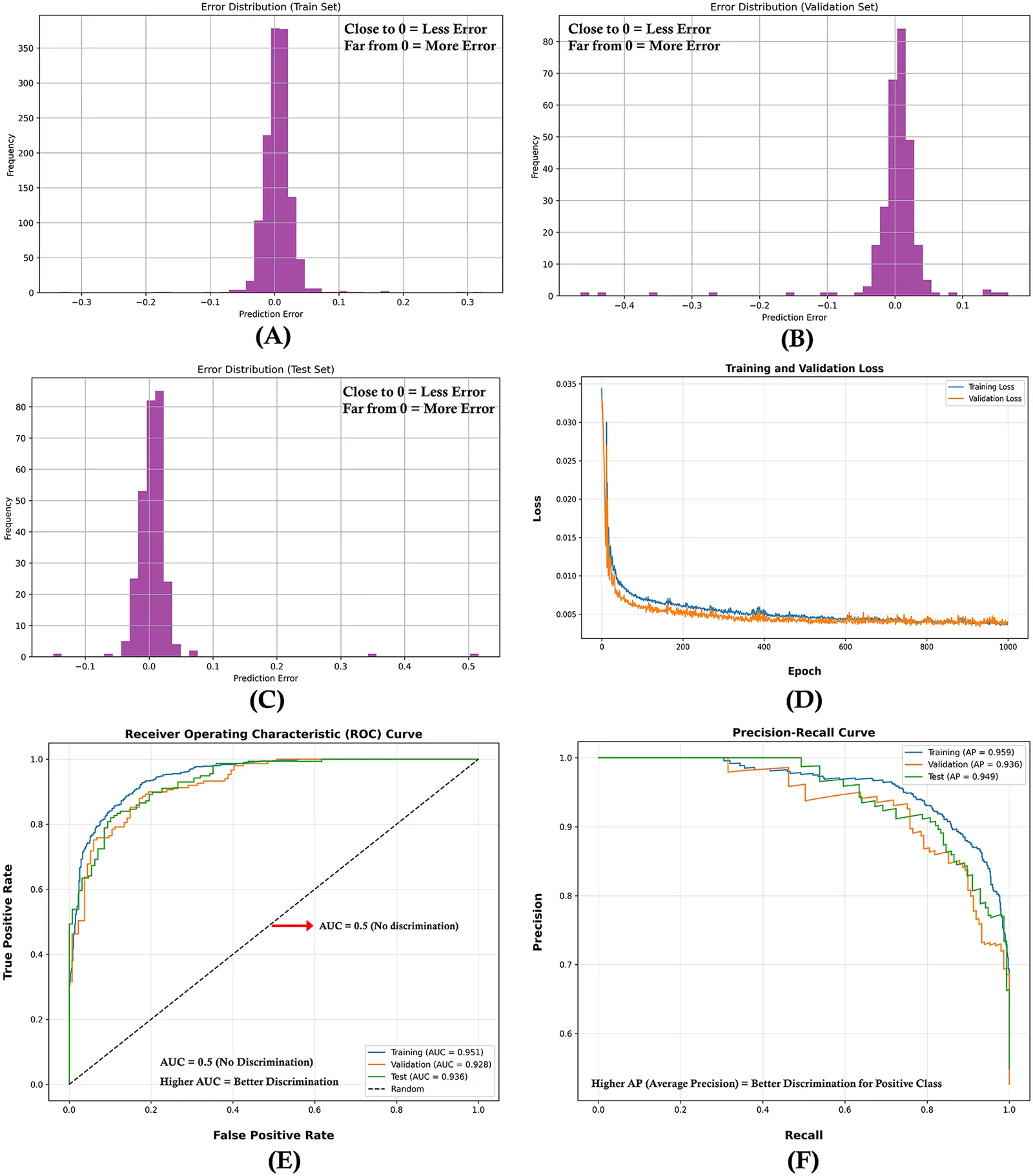
Performance evaluation of the graph neural network model. **(A)**, **(B)**, **(C)** Error distribution of training, validation, and testing datasets. **(D)** Training and validation data loss over 200 epochs. **(E)** ROC curve and **(F)** PR curve. Here, AUC = area under the curve, and AP = average precision. AUC and AP values range from 0 to 1, where 1 indicates a perfect classifier.

Understanding the molecular basis of GC requires identifying genes that exhibit both dysregulated expression and high mutation propensity, as these represent critical drivers of oncogenesis. Our graph-based neural network model, developed using differentially expressed genes, mutation-prone genes, common genes between DEGs and mutation-prone genes, protein-protein interaction network, and GC pathway genes, revealed a substantial overlap between GC-specific mutation-prone genes and differentially expressed genes (DEGs) (S1–S5 Tables, respectively). As illustrated in Fig 2A, we identified 10,981 mutation-prone genes and 3,741 differentially expressed genes, with 1,886 genes (50.4% of DEGs) showing both characteristics. The network visualization highlights the protein-protein interaction landscape of these genes, with red nodes representing the most important GC pathway-specific genes that serve as key hubs within the network (Fig 2B). Fig 2C depicts our GCN architecture with two hidden layers. The GCN model takes an input graph, processes it through two hidden layers with ReLU activation, and produces an output graph. Each layer consists of multiple nodes with weighted connections to highlight relationships within the graph, indicated by the color-coding of nodes and edges. Our graph neural network model effectively identified differentially expressed mutation-prone genes with exceptional accuracy across all datasets. The model demonstrated robust predictive capabilities after 5-fold nested cross-validation (CV) with low mean square error values (MSE: 0.00482 ± 0.00023 to 0.07108 ± 0.00437) and strong goodness-of-fit metrics (R²: 0.85098 ± 0.01903 to 0.85899 ± 0.01987) across training, validation, and testing datasets (Table 1). The error distribution histograms for training (Fig 3A), validation (Fig 3B), and testing (Fig 3C) datasets showed tightly clustered prediction errors around zero, confirming the model’s precision. The learning curve (Fig 3D) demonstrated rapid convergence within 50 epochs, stabilizing training and validation losses, indicating proper model fitting without overfitting. High discriminative power was confirmed through impressive AUC-ROC scores (Fig 3E) of 0.93287 ± 0.00373, 0.93095 ± 0.01758, and 0.93309 ± 0.00725 for training, validation, and testing datasets, respectively, significantly outperforming random classification (diagonal line). Similarly, the precision-recall curves (Fig 3F) yielded strong AUC-PR values (0.93879 ± 0.01815 to 0.94772 ± 0.00214), further validating the model’s reliability. Through this computational approach, we identified the top 20 GC pathway-specific dysregulated mutation-prone genes, with TP53 exhibiting the highest mutation frequency (47.6%), followed by ERBB2 (8.8%), CDH1 (8.2%), and APC (6.8%) (Table 2). Notably, 17 of these genes were confirmed to be present in established GC pathways, with importance scores exceeding 0.99. The network analysis revealed that TP53 exhibited the highest degree (0.239), betweenness centrality (0.081), and closeness centrality (0.521), indicating its crucial role as a hub gene in GC progression. Importance scores are derived based on co-expression, experimentally determined interaction, database annotation, automated text mining, and a combined score from the STRING database. On the other hand, the predicted scores are the final calculated scores from the model. For further analysis, we have selected TP53, CDH1, and APC based on their most important pathway-specific high-frequency mutation states and tumor suppressor capabilities.

**Table 1:** Performance Metrics of training, testing, and validation datasets of differentially expressed mutation-prone genes with ± SD (standard deviations).

Dataset	MSE	RMSE	R²	AUC-ROC	AUC-PR
**Training** **(60% datasets)**	0.00482 ± 0.00023	0.06940 ± 0.00169	0.85620 ± 0.00798	0.93287 ± 0.00373	0.94772 ± 0.00214
**Validation** **(20% datasets)**	0.00528 ± 0.00081	0.07250 ± 0.00563	0.85899 ± 0.01987	0.93095 ± 0.01758	0.93879 ± 0.01815
**Testing** **(20% datasets)**	0.00507 ± 0.00062	0.07108 ± 0.00437	0.85098 ± 0.01903	0.93309 ± 0.00725	0.94556 ± 0.00847

**Table 2:** Graph neural network-based prediction of GC pathway-specific dysregulated mutation-prone genes and mutation frequency from the cBioPortal database.

Genes	Degree	Betweenness	Closeness	Present in Pathway	Importance Score	Predicted Score	Mutation Frequency
TP53	0.239	0.081	0.521	Yes	0.990	0.960	47.60%
CTNNB1	0.162	0.038	0.49	Yes	0.965	0.935	6.10%
MYC	0.19	0.04	0.5	Yes	0.948	0.931	1.40%
CDH1	0.102	0.012	0.461	Yes	0.899	0.889	8.20%
GSK3B	0.09	0.012	0.457	Yes	0.892	0.888	2.00%
ERBB2	0.1	0.012	0.458	Yes	0.921	0.888	8.80%
CCND1	0.1	0.006	0.454	Yes	0.904	0.879	0.70%
LRP5	0.023	0.001	0.383	Yes	0.947	0.878	1.40%
LRP6	0.021	0.001	0.378	Yes	0.870	0.877	2.00%
LEF1	0.049	0.004	0.414	Yes	0.867	0.869	0.70%
SMAD3	0.067	0.005	0.437	Yes	0.880	0.866	2.70%
EP300	0.107	0.01	0.46	No	0.861	0.865	2.70%
GRB2	0.08	0.011	0.443	Yes	0.853	0.865	0.70%
HNF4A	0.046	0.004	0.42	No	0.870	0.862	0.70%
CDH17	0.04	0.004	0.409	Yes	0.864	0.860	2.00%
WNT2	0.019	0.001	0.382	Yes	0.859	0.860	1.40%
APC	0.02	0	0.389	Yes	0.844	0.854	6.80%
TCF7L2	0.025	0.001	0.389	Yes	0.812	0.857	0.70%
CDKN2A	0.086	0.009	0.449	No	0.864	0.839	2.70%
TOP2A	0.111	0.005	0.448	No	0.834	0.837	1.50%

### 3.2. Tumor suppressor proteins of the GC pathway

**Fig 4:**
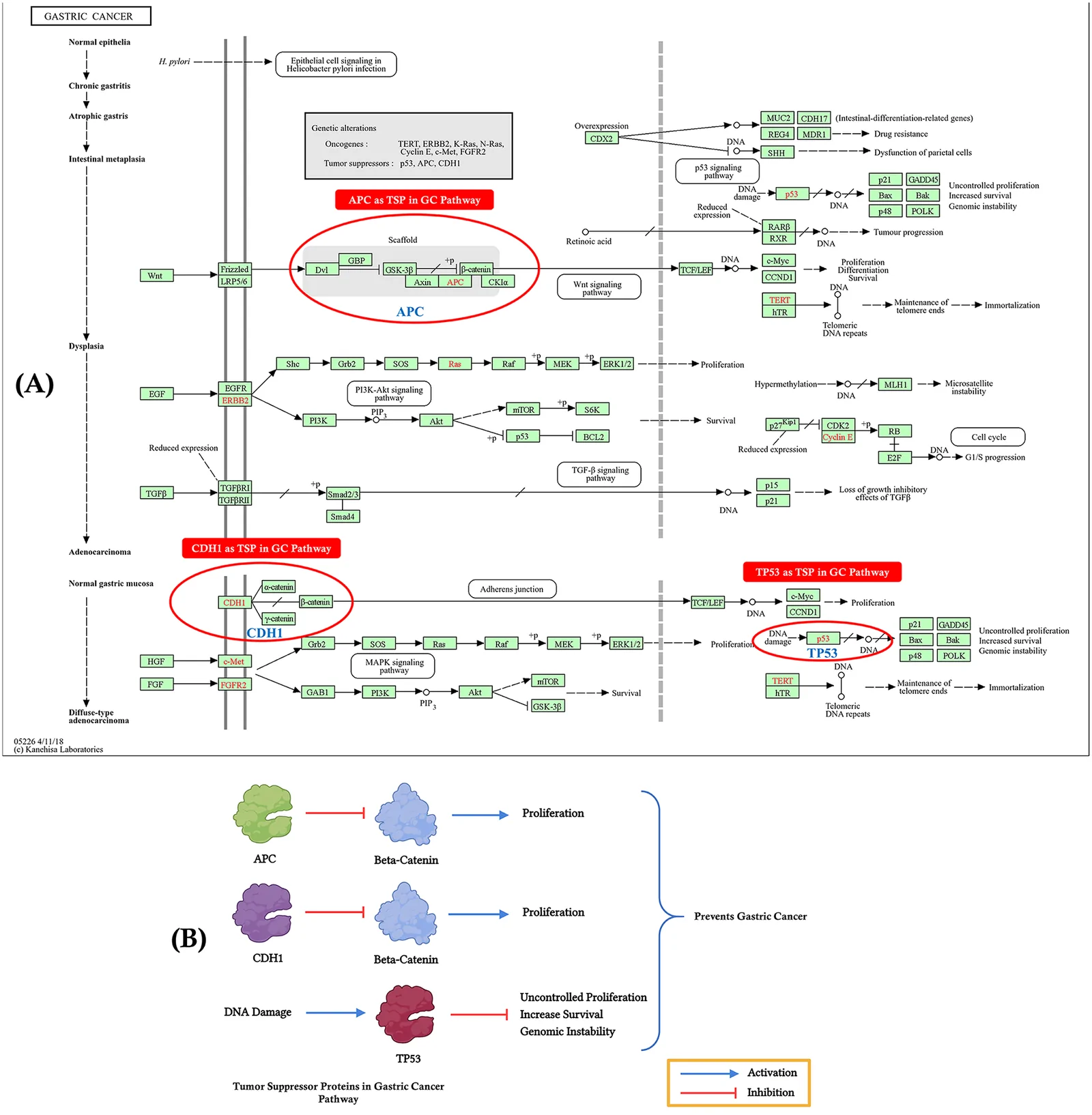
Tumor suppressor proteins mediated the regulation of the GC pathway. **(A)** GC pathway from the KEGG database. **(B)** Functional consequences of APC, CDH1, and TP53 in suppressing GC development [31].

Our analysis of the GC pathway from the KEGG database revealed that TP53, APC, and CDH1 are key tumor suppressor proteins implicated in the GC pathway in three distinct mechanisms (Fig 4A). However, functional analysis revealed that both APC and CDH1 inhibit beta-catenin, thereby preventing the uncontrolled proliferation of cancer cells. In contrast, TP53 is activated in response to DNA damage to inhibit uncontrolled proliferation, increase survival, and mitigate genomic instability [31]. The functional consequences of these three crucial proteins that play key roles in suppressing GC have been presented in Fig 4B.

### 3.3. Mapping tumor suppressor domains of proteins

**Fig 5:**
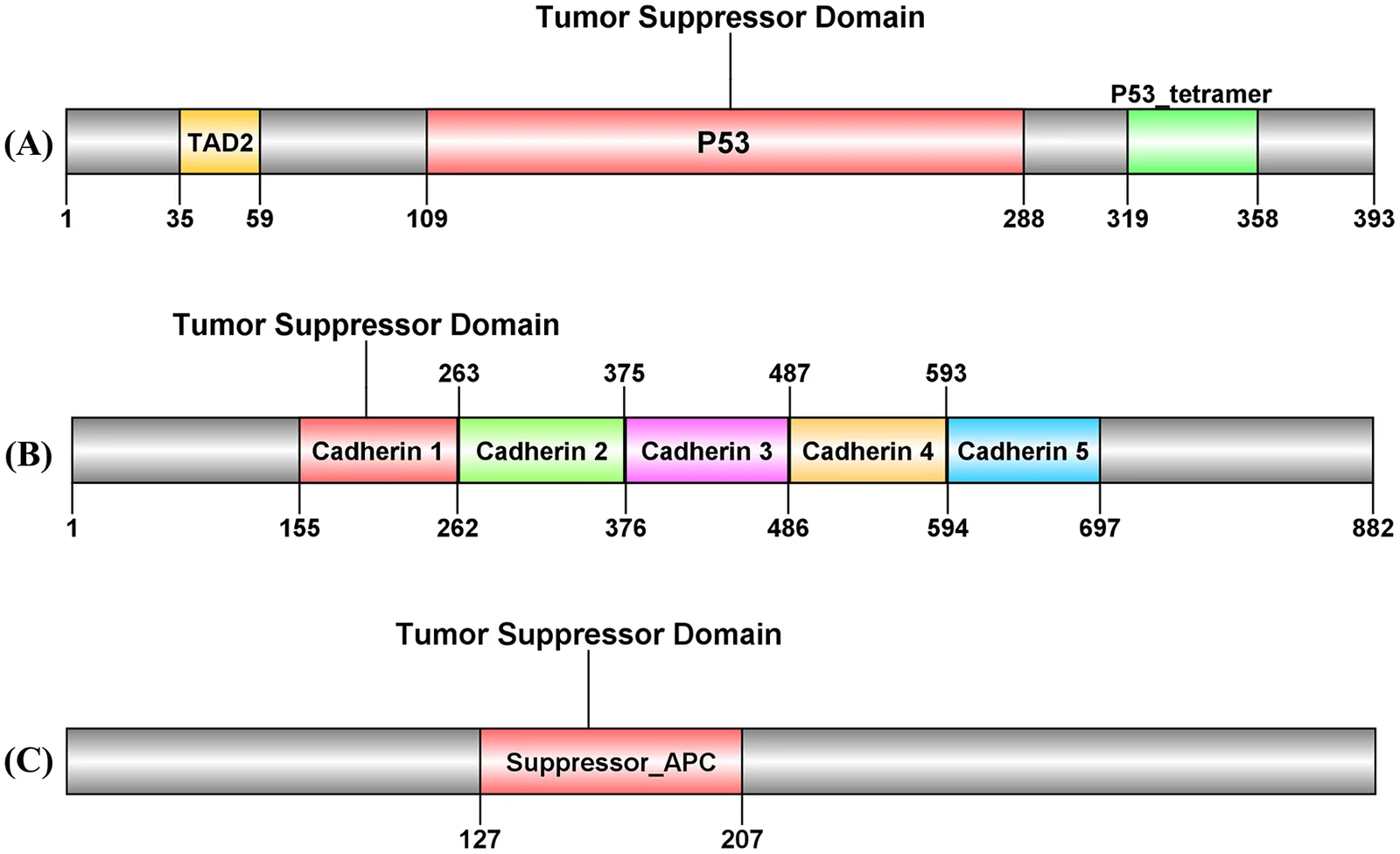
Tumor Suppressor Domains of Proteins. **(A)** TP53, **(B)** CDH1, and **(C)** APC.

Mapping tumor suppressor domains is essential to precisely identify and characterize the specific functional regions within key tumor suppressor proteins. This enables a deeper understanding of their roles in cancer prevention and facilitating targeted therapeutic development. We investigated the tumor suppressor domains of TP53, APC, and CDH1 and revealed specific domains essential for their tumor-suppressive functions. Specifically, the TP53 protein exhibits a tumor suppressor domain spanning amino acid positions 109–288, while APC encompasses a domain from positions 127–207, and CDH1 features a domain from positions 155–262. The tumor suppressor domains have been represented in Fig 5.

### 3.4. Retrieval of clinical variations

**Fig 6:**
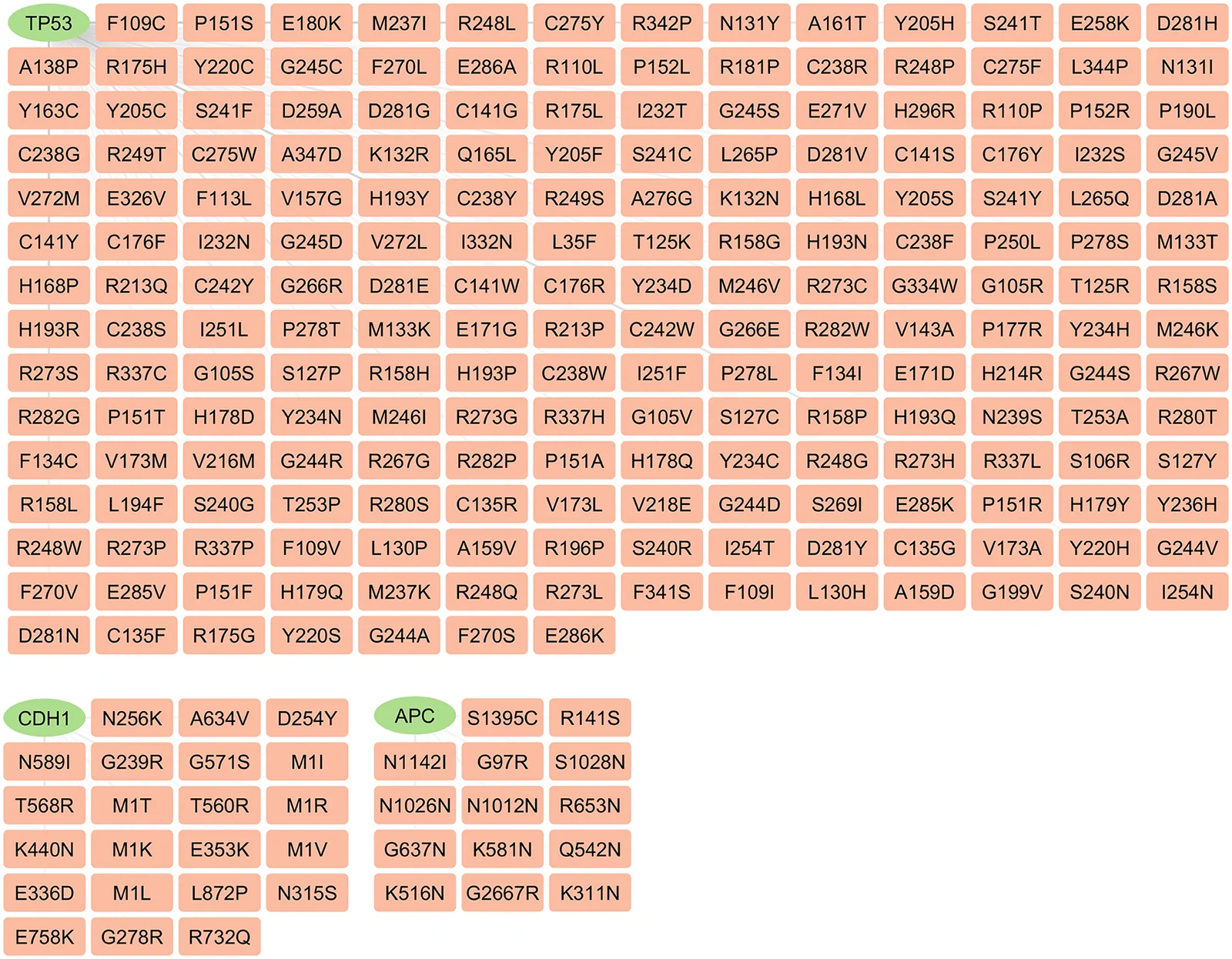
Pathogenic or potentially pathogenic clinically significant missense SNPs that could have a link with GC pathogenesis. Green Circle: Tumor Suppressor Proteins and Red Box: Missense SNPs.

The ClinVar database consists of frameshift, missense, nonsense, splice site, ncRNA, and UTR (untranslated region) variations. The pathogenic variations were initially retrieved from the database for TP53 (936), CDH1 (559), and APC (2370). Besides, for missense SNPs, both pathogenic and likely pathogenic variations were considered for further analysis. General missense SNPs from the dbSNP database are considered as common variants with uncertain clinical significance, whereas missense SNPs from ClinVar are curated and annotated variants with known or predicted clinical relevance and phenotypic disease associations. Therefore, from a total of 11,393 identified missense SNPs across the three selected proteins: APC, CDH1, and P53, only 216 missense SNPs of TP53, 19 of APC, and 22 of CDH1 were classified as clinically significant pathogenic or potentially pathogenic based on the NCBI ClinVar database that could have functional consequences and directly contribute to GC pathogenesis (Accessed at 24 February, 2025). The list of retrieved missense SNPs was incorporated in the form of the latest chromosome 38 version (GRCh38) in Fig 6.

### 3.5. Damaging, deleterious, and disease-causing missense SNPs in tumor suppressor proteins

This computational analysis was performed to systematically identify and characterize deleterious missense SNPs in tumor suppressor proteins, thereby elucidating their potential role in cancer development and progression. Through comprehensive analysis, we identified 54 missense SNPs in the TP53 gene that were consistently classified as “high-risk,” as they were unanimously predicted to be “pathogenic” or “damaging” by all the employed tools. Similarly, 9 missense SNPs in APC and 11 missense SNPs in CDH1 were also defined as “high-risk” based on their consensus classification as “pathogenic” or “damaging” across all assessment approaches. The full list of these high-risk missense SNPs has been provided in Table 3.

**Table 3:** List of potential GC pathway-disrupting missense SNPs.

Proteins	SNPs	Alleles	SIFT, PolyPhen2, SNPS&GO, PHD-SNP, SNPs3D, GVGD	I-Mutant 2.0, MuPro	Missense3D, HOPE	Cscape
TP53	rs587781525	D281A	Damaging, Deleterious, Disease-Causing	Decrease Stability of Protein	Structural Damage	Oncogenic
	rs587781525	D281G				
	rs764146326	D281H				
	rs28934576	R273P				
	rs121913343	R273G				
	rs193920774	G266E				
	rs1057519990	G266R				
	rs879253942	L265P				
		I254N				
		T253P				
	rs11540652	R248P				
	rs587780074	M246K				
	rs2073251813	G245D				
	rs121912656	G245V				
	rs28934575	G245S				
	rs28934575	G245C				
	rs985033810	G244A				
	rs985033810	G244V				
	rs985033810	G244D				
	rs1057519989	G244R				
	rs1057519989	G244S				
	rs375874539	C242W				
	rs121912655	C242Y				
	rs1567549584	S240R				
	rs1567549584	S240G				
	rs193920789	C238W				
	rs730882005	C238F				
	rs730882005	C238Y				
	rs1057519981	C238G				
	rs1057519981	C238R				
	rs587782289	Y236H				
	rs864622237	Y234H				
	rs864622237	Y234D				
	rs121912666	Y220C				
	rs121912666	Y220S				
	rs530941076	Y220H				
		V218E				
	rs587778720	R213P				
	rs1555525857	G199V				
	rs483352697	R196P				
	rs876658468	H193N				
	rs1567552847	C176R				
	rs786202962	C176F				
	rs786202962	C176Y				
	rs28934578	R175L				
		rs138729528					R175G				
	rs1555526131	A159D				
	rs1057520000	P151R				
	rs28934875	A138P				
	rs1057519975	C135R				
	rs866775781	K132N				
	rs1057519996	K132R				
	rs786201057	T125R				
	rs786201057	T125K				
CDH1	rs1555515445	G239R				
	rs759207947	D254Y				
APC	rs863224458	R141S				
	rs879254090	K516N				
	rs1554083227	G637N				

### 3.6. Impact of missense SNPs on protein structural stability

The assessment of protein structural stability is required to elucidate the structural implications of missense SNPs on tumor suppressor proteins, thereby providing insight into their potential oncogenic effects. This assessment revealed that the majority of the identified high-risk missense SNPs exerted a detrimental impact on the structural integrity of the corresponding tumor suppressor proteins. Specifically, all of the 54 missense SNPs in TP53, 9 in APC, and 7 in CDH1 were found to significantly decrease the overall structural stability of these critical proteins, as predicted by the I-Mutant 2.0 and MUpro computational tools (Table 3).

### 3.7. Effects of missense SNPs on protein 3D Structure

Computational analysis through Missense3D and HOPE servers is required to predict the structure-disrupting effects of missense SNPs on protein 3D structure. Using computational analyses through Missense3D and the HOPE servers, we evaluated the effects of missense SNPs on the three-dimensional structures of tumor suppressor proteins TP53, APC, and CDH1. Our findings revealed 54 structural damages in TP53, three in APC, and two in CDH1, indicating significant alterations in protein conformation attributable to these SNPs. Furthermore, the HOPE scores for the point mutations corroborated the potential deleterious impacts on protein 3D structure (Table 3).

### 3.8. Protein evolutionary conservation sites

**Fig 7:**
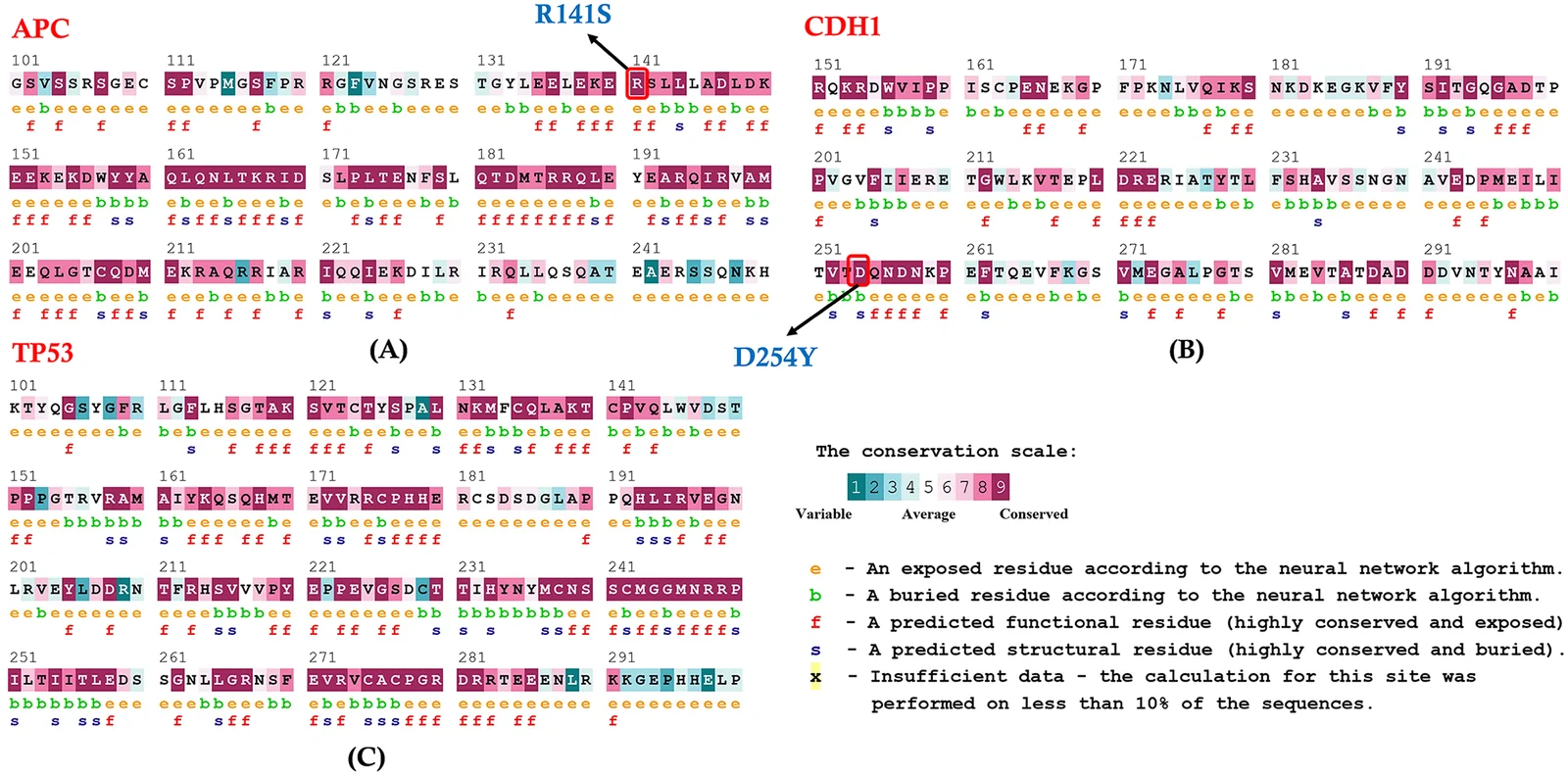
Evolutionary conservation in the region of the representative domain of proteins. (A) APC, (B) CDH1, and (C) TP53. Conservation scores 6-9 (Purple) are considered highly conserved amino acid positions.

Protein evolutionary conservation analysis is required to identify evolutionarily conserved sites within tumor suppressor domains of TP53, APC, and CDH1 proteins. This highlights critical regions where mutations are likely to have significant functional consequences and contribute to tumorigenesis. The tumor suppressor proteins TP53, APC, and CDH1 revealed a high degree of evolutionary conservation throughout the tumor suppressor domains. The analyzed 54 TP53 SNPs exhibited a ConSurf conservation score of 7–9, indicating highly conserved, surface-exposed residues. Similarly, APC R141S and CDH1 D254Y showed a ConSurf score of 9, indicating their critical role within the tumor suppressor domain. This high conservation suggests that alterations at these sites may have profound effects on protein function and tumorigenesis. In contrast, CDH1 G239R displayed a lower score of 6, also suggesting good conservancy but potentially less stringent selective pressure at this site than APC R141S and CDH1 D254Y. Protein conservancy is shown in Fig 7.

### 3.9. Estimation of protein energy

The energy estimation and minimization analysis revealed insights into the immediate structural instability of mutant proteins compared to wild-type proteins. The mutations in the APC protein at position R141S and the CDH1 protein at position D254Y exhibited high energy values, indicating that the overall protein structure was unfavorable. This suggests that these mutations may have a significant impact on the structural integrity and stability of proteins. In contrast, the TP53 protein had a high number of mutated sites with 42 high-energy values, including 31 abnormally high energy values, often indicating that the protein is not in its ideal or stable state and is therefore at high risk. Specific mutations in TP53, such as D281A, D281G, D281H, R273P, R273G, G266E, G266R, and L265P, displayed particularly high energy minimization values, further suggesting the potential instability of these mutant forms of the protein. Protein Energy estimation before and after minimization, together with the wild-type form of proteins, was represented in S6 Table.

### 3.10. Post‑translational modification sites

**Fig 8:**
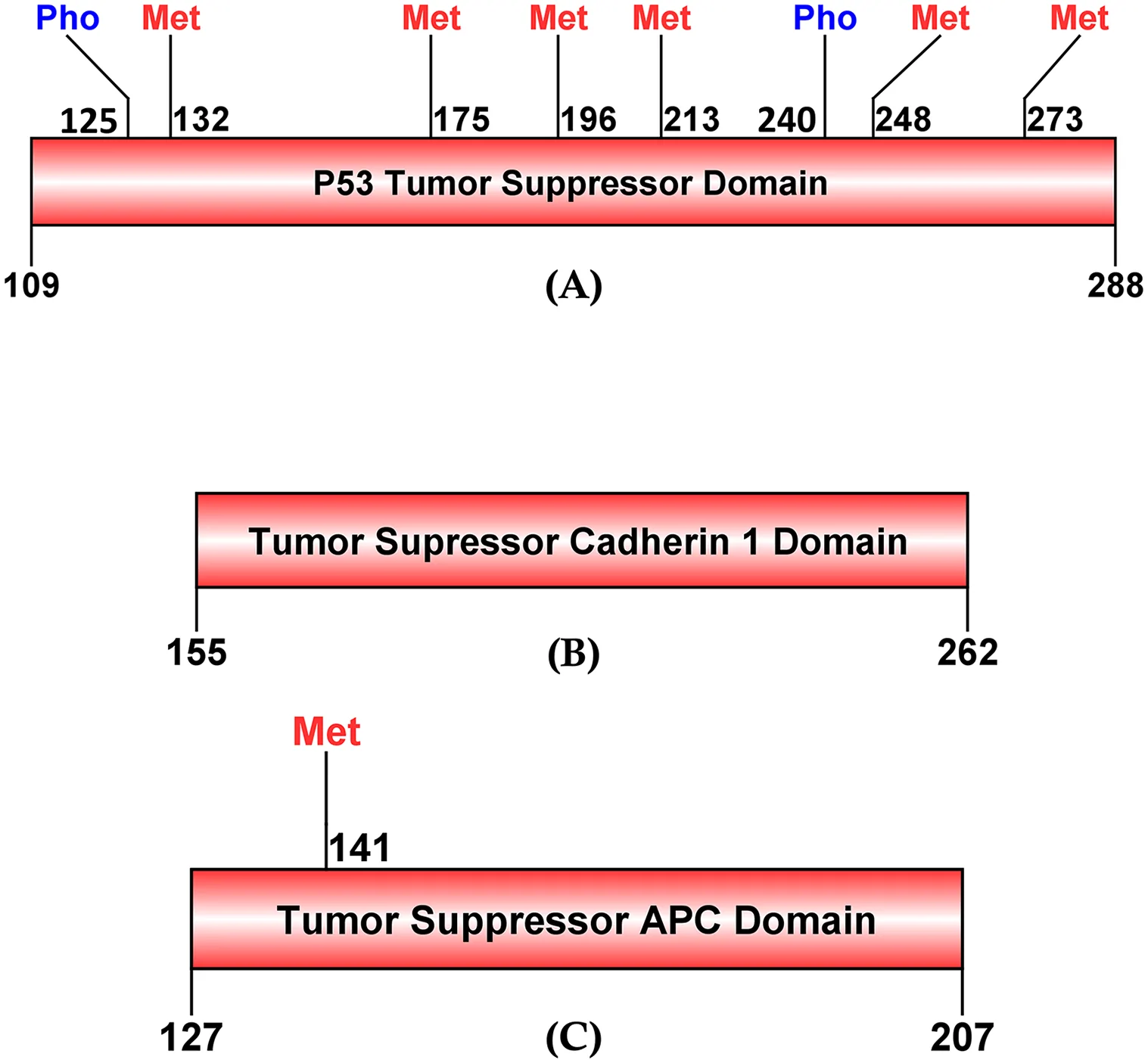
Post-translational modification sites of proteins. **(A)** TP53, **(B)** CDH1, and **(C)** APC. TP53 contains both Methylation and Phosphorylation sites, CDH1 does not contain any PTM sites, and AMP contains 1 Methylation site in the tumor suppressor domain regions.

The post-translational modification site analysis using GPS-MSP 1.0 and GPS-6.0 is essential to identify potential regulatory modifications that may influence the functional activity and stability of tumor suppressor proteins to elucidate mechanisms underlying their dysregulation in cancer. This analysis, using the GPS-MSP 1.0 and GPS-6.0 tools, revealed several post-translational modification (PTM) sites in the tumor suppressor genes TP53, APC, and CDH1. Specifically, the GPS-MSP 1.0 tool predicted nine methylation sites in TP53, one methylation site in the tumor suppressor domain of APC, and no methylation sites in the tumor suppressor domain of CDH1. Furthermore, the GPS-6.0 tool identified four phosphorylation sites in TP53, but no phosphorylation sites in APC or CDH1. Notably, no protein ubiquitin ligase sites were detected in the tumor suppressor domains of these genes. The PTM sites are represented in Fig 8.

### 3.11. Validation of clinically significant missense SNPs from GC patients

**Fig 9:**
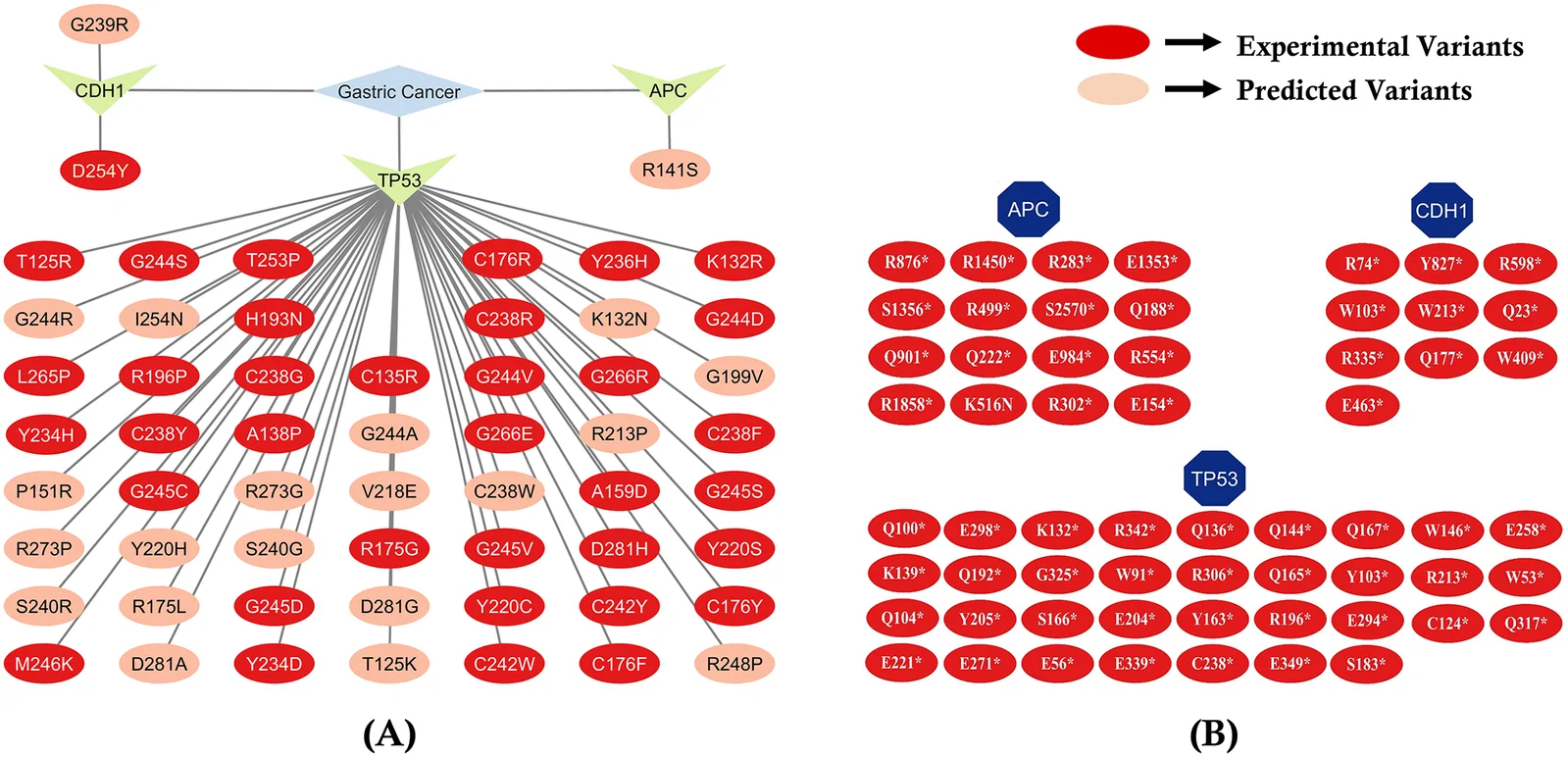
Mutations disrupting GC suppression proteins. **(A)** Missense SNPs, and **(B)** Nonsense SNPs. Light Green Triangle: Tumor suppressor Protein, Dark Red Circle: experimentally validated pathogenic SNPs in GC, and Light Red Circle: missense SNPs that may disrupt GC prevention.

Validation of clinically significant missense SNPs is required to confirm the significance and relevance of identified missense SNPs in GC patients and establish their potential association with the GC and tumor suppressor genes TP53, APC, and CDH1. The validation of clinically significant missense SNPs was conducted using cBioPortal, analyzing data from 5,623 samples of GC patients across 22 studies. The results indicated that 35 dangerous missense SNPs in the TP53 gene were identified as clinically significant and directly associated with GC, while no clinically significant SNPs were found in the APC gene. Additionally, one clinically significant, dangerous missense SNP was detected in the CDH1 gene, as illustrated in Fig 9. Validation of missense SNPs using cBioPortal confirmed the clinical relevance of the identified oncogenic missense SNPs and their direct association with GC. Apart from missense SNPs, other pathogenic clinical variations, such as frameshift, nonsense, splice site, ncRNA, and UTR, were also validated from the cBioPortal database to identify mutations that can alter the balance of the GC pathway through the disruption of tumor suppressor proteins. Our analysis found that only missense (36) and nonsense (60) pathogenic variations were linked to GC pathogenesis. The list of all validated pathogenic clinical variations of TP53, CDH1, and APC is listed in S7, S8, and S9 Tables, respectively.

### 3.12. Comparison of energetic patterns between wild-type and mutant proteins

Titration curve analysis between wild-type and mutant proteins enables direct comparison of their ionization behaviors, revealing shifts in pKa values and protonation states that could indicate potential impact on protein stability, function, and intermolecular interactions. S2 Fig shows the analysis of the titration curves for TP53, revealing highly similar protonation behavior between wild-type (pI = 8.6) and T125R mutant (pI = 8.9) variants. Both proteins exhibit a characteristic sigmoidal curve with a maximum net charge of approximately +30 at pH 2, gradually decreasing to −20 at pH 12. The subtle difference in isoelectric points suggests the minimal impact of the T125R mutation on the protein’s overall charge distribution, with near-identical titration behavior observed across the entire pH range. The CDH1 wild-type (pI = 4.3) and D254Y mutant (pI = 4.3) proteins demonstrated identical isoelectric points but showed distinct titration patterns. The curves initiate at approximately +15 net charge at pH 2, converge through the isoelectric point, and progressively decrease to approximately −30 at pH 12. Despite sharing the same pI, subtle variations in the curves between pH 6–10 suggest localized effects of the D254Y mutation on charge distribution, while maintaining the protein’s overall acidic character. APC titration curves revealed minimal differences between wild-type (pI = 5.5) and R141S mutant (pI = 5.3) forms. Both variants displayed similar titration behaviors, starting at +20 net charge at pH 2, crossing the isoelectric point near pH 5, and declining to −15 at pH 12. The marginal 0.2 unit decrease in pI for the R141S mutant indicates a slight enhancement in overall protein acidity, though the nearly superimposable nature of the curves suggests conservation of the protein’s general charge characteristics despite the mutation.

**Fig 10:**
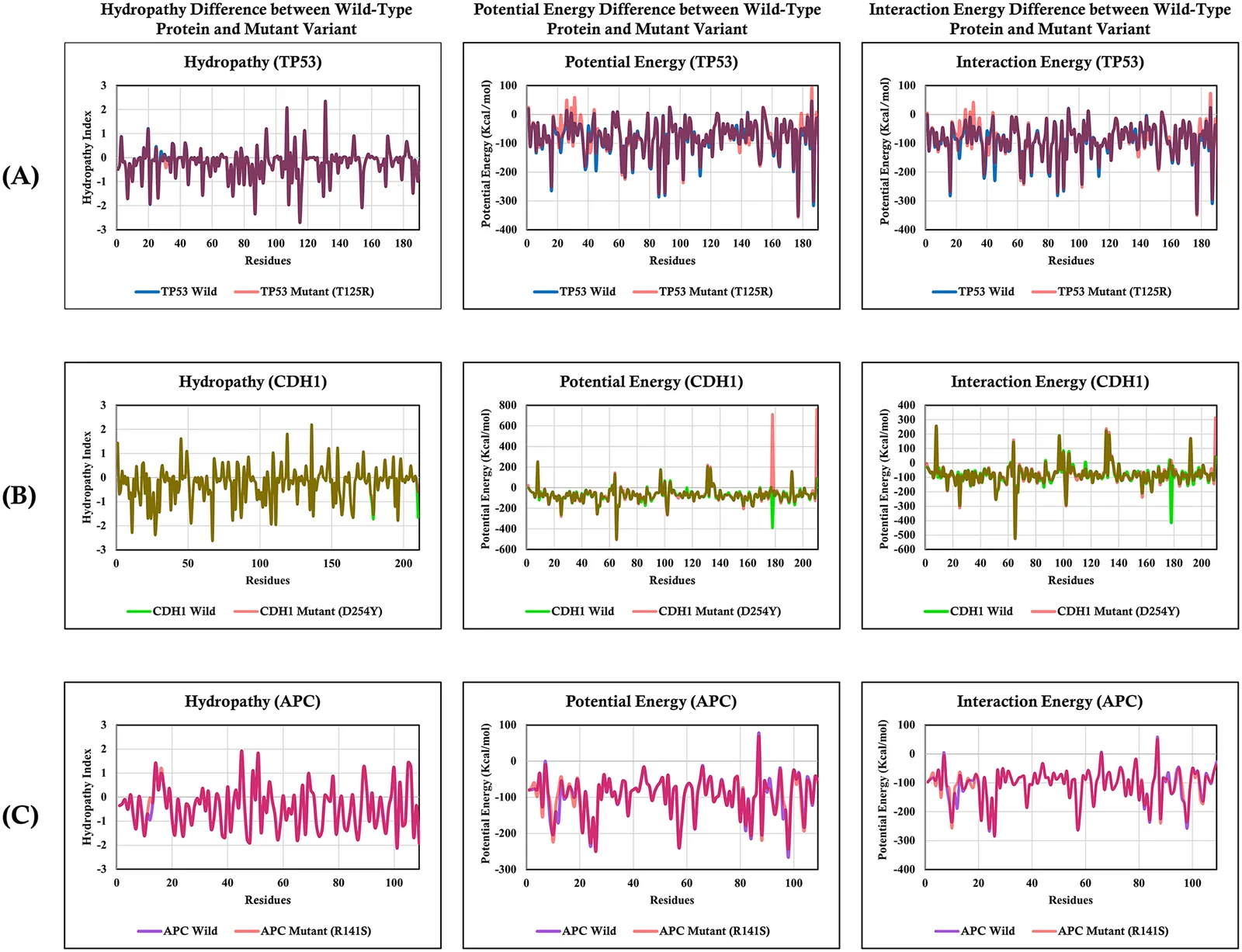
Analysis and comparison of hydropathy, potential energy, and Interaction energy. **(A)** TP53, **(B)** CDH1, and **(C)** APC. Both the potential energy and interaction energy shifts were observed in TP53 at amino acid region 20-44, and CDH1 at amino acid region 178-179.

Moreover, comparison of hydropathy, potential energy, and interaction energy between wild-type and mutant proteins reveals crucial biophysical perturbations that could affect protein conformational stability, folding dynamics, binding affinity, functional interactions, protein-ligand interactions, and solvation by quantifying changes in molecular forces and thermodynamic properties. Fig 10 displays the hydropathy, potential energy, and interaction energy plots for TP53, CDH1, and APC. Hydropathy profiles revealed distinct biophysical alterations across the three protein systems. The T125R mutant of TP53 maintained a near-identical hydropathic character to the wild-type, with localized fluctuations observed in amino acid residues 23, 26, and 29 where the hydropathy index of the wild-type was higher than the mutant T125R. In contrast, the hydropathy index of the wild type was lower than the mutant T125R in residue 32, suggesting a minimal deviation from the hydrophobic core organization. The CDH1 D254Y mutant exhibited less negative deviation than the wild type in residues 179, 180, and 211 (Green). The APC R141S mutant demonstrated minor deviation in residues 10 and 13. In the case of potential energy distributions that highlight thermodynamic instability in mutant variants, the T125R mutant of TP53 showed marginal deviations in residues 17–48, 138–143, 146, 180–194. D254Y mutant of CDH1 displayed a striking (−391.263 to +711.939 kcal/mol) spike near residue 178–183, exceeding wild-type values by 1103.192 kcal/mol, suggesting potential electrostatic repulsion in the extracellular cadherin repeat. Another potential deviation (+669.680 kcal/mol) was observed in mutant residue 211. R141S mutant of APC roughly deviated from wild type in residues 1–20 and 83–105. Subsequently, for molecular interaction energy patterns, TP53’s mutant T125R showed conserved interaction energies except at residues around 20–45, 135–145, and 177–211. In most cases, only the mutant T125R crosses the positive interaction energy boundaries, which could alter p53-MDM2 binding kinetics. On the other hand, the CDH1’s wild type manifested a −400 kcal/mol interaction energy peak at residue 179, and CDH1’s mutant D254Y manifested a + 300 kcal/mol interaction energy peak at residue 211. APC’s mutant R141S retained wild-type-like interaction energies between −300 to +100 kcal/mol except in the region (residues 2–19).

### 3.13. Atomic evaluation of proteins utilizing Unsupervised machine learning

**Fig 11:**
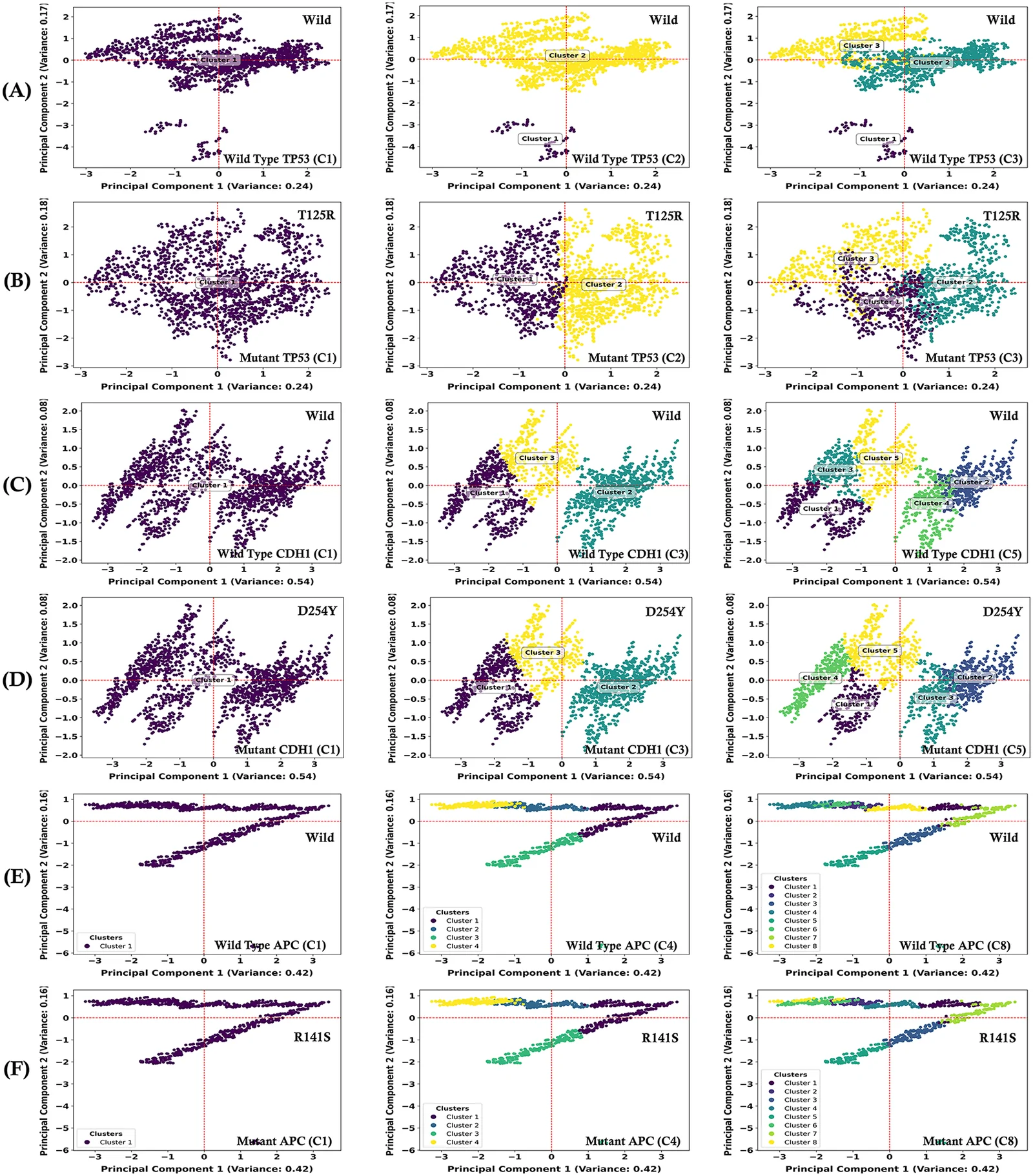
Cluster analysis using the unsupervised k-means clustering algorithm through dimensionality reduction. **(A)**, **(C)**, **(E)** Wild-type versions of TP53, CDH1, and APC, respectively, and **(B)**, **(D)**, **(F)** Mutant versions of TP53 (T125R), CDH1 (D254Y), and APC (R141S), respectively. C1 to C8 denotes the number of clusters where the initial atomic distribution in the clusters was the same; however, atomic distribution changes throughout the increasing number of clusters due to mutation.

Atomic-level evaluation and comparison of wild-type and missense mutant proteins through unsupervised machine learning may enable the detection of subtle intrinsic conformational changes, pattern recognition, and scalability. The atomic content of TP53, CDH1, and APC of both wild and mutant types has been utilized for an unsupervised machine learning algorithm (S10A-F Table). Native clusters of wild-type TP53 containing the atomic content of particular amino acids should be maintained to retain tumor suppressor activity (Table 4). In TP53, the first cluster group showed variations between wild type and mutant type (T125R) in cluster 1, indicating the most prominent atoms that were required to maintain the tumor suppressor activity of TP53. Till the analysis of cluster group 3, this scenario continued, which confirmed that T125R disrupted the atomic associations of particular amino acids responsible for tumor suppressor activity modulation. The structural and functional disrupting impact of missense SNPs T125R, D254Y, and R141S in TP53, CDH1, and APC provides hypothesis-generating outcomes from the disruption of Cluster 1, Cluster 3, and Cluster 4, respectively. For clusters of TP53 and APC, both contain 13 crucial amino acids in the tumor suppressor domain region that were extremely affected. On the other hand, the cluster of CDH1 contains 50 amino acids, the atomic content of which could be responsible for the distortion of the atomic content. The disruption of the atomic pattern was clearly visualized in wild-type TP53 and mutant-type TP53 (Figs 11A and 11B); however, the subtle variation was only observed in the case of CDH1 (Figs 11C and D) and APC (Figs 11E and F) after analysis of cluster groups 5 and 8, respectively.

**Table 4:** Native clusters containing atomic content of particular amino acids are retained in the wild-type tumor suppressor protein, but these patterns are messed up in the mutant type, possibly disrupting structural and functional consequences. The amino acids in clusters 1, 3, and 4 for TP53, CDH1, and APC were retained in wild type but were messed up in mutant type (T125R of TP53), (D254Y of CDH1), and (R141S of APC), respectively.

Clusters of Amino Acids		
**TP53 (Cluster 1)**	**CDH1 (Cluster 3)**	**APC (Cluster 4)**
THR150, PRO151, PRO152, PRO153, GLY154, ARG202, GLU221, PRO222, PRO223, GLU224, VAL225, GLY226, SER227	PHE108, ALA121, LEU122, PRO123, GLY124, THR125, SER126, VAL130, THR131, TYR148, THR149, ILE150, LEU151, SER152, GLN153, ASP154, PRO155, GLU156, LEU157, PRO158, ASP159, LYS160, ASN161, MET162, PHE163, THR164, ILE165, THR169, GLY170, VAL171, ILE172, SER173, VAL174, VAL175, THR176, THR177, GLY178, LEU179, TYR187, THR188, LEU189, VAL190, VAL191, GLN192, ALA193, SER202, THR203, THR204, ALA205, ILE209	GLN223, LYS226, ASP227, LEU229, ARG230, ILE231, ARG232, GLN233, LEU234, LEU235, GLN236, SER237, GLN238

### 3.14. Link among missense SNPs, protein expression, and patient survival

**Fig 12:**
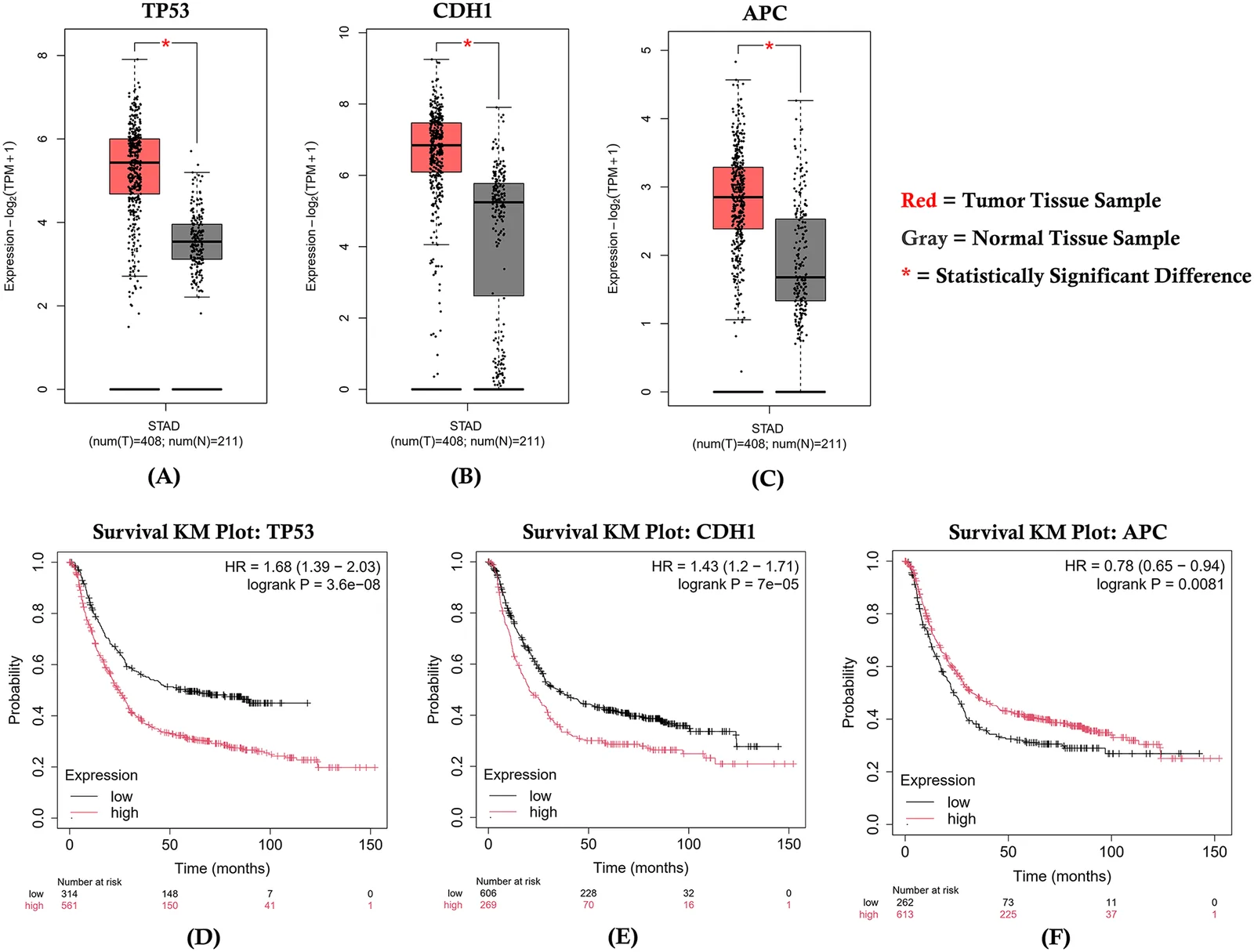
Protein expression and patient survival differences. Plots show significant expression of proteins **(A)** TP53, **(B)** CDH1, **(C)** APC, and significant survival differences in proteins **(D)** TP53, **(E)** CDH1, and **(F)** APC.

Evaluating protein expression and its association with patient survival in GC revealed that tumor suppressor genes TP53, CDH1, and APC were significantly upregulated in GC-developing tissues (Figs 12A, B, and C). Subsequently, survival analysis was assessed using the log-rank test, indicating a significant difference in patient outcomes associated with these genes, with FDR-adjusted p-values of 3.6e-08, 7e-05, 0.0081 for TP53, CDH1, and APC, respectively, with an FDR (False Discovery Rate) cutoff set to 1% for all. Moreover, the hazard ratios of TP53, CDH1, and APC were 1.68, 1.43, and 0.78, respectively (Figs 12D, E, and F). The survival was evaluated using the difference calculations between the low expression cohort (months) 57.63, 44.07, 24.03, and the high expression cohort (months) 24.5, 24.4, 57.8 for TP53, CDH1, and APC, respectively. Moreover, across TP53, APC, and CDH1, hazard ratios progressively increase with advancing cancer stage, indicating that although early stages show relatively protective effects, the prognostic risk consistently escalates in higher stages, reflecting stage-dependent worsening survival impact. The S11 Table presents significant covariate analysis of survival in different stages of cancer.

### 3.15. Networks and cancer hallmarks of tumor suppressor proteins

**Fig 13:**
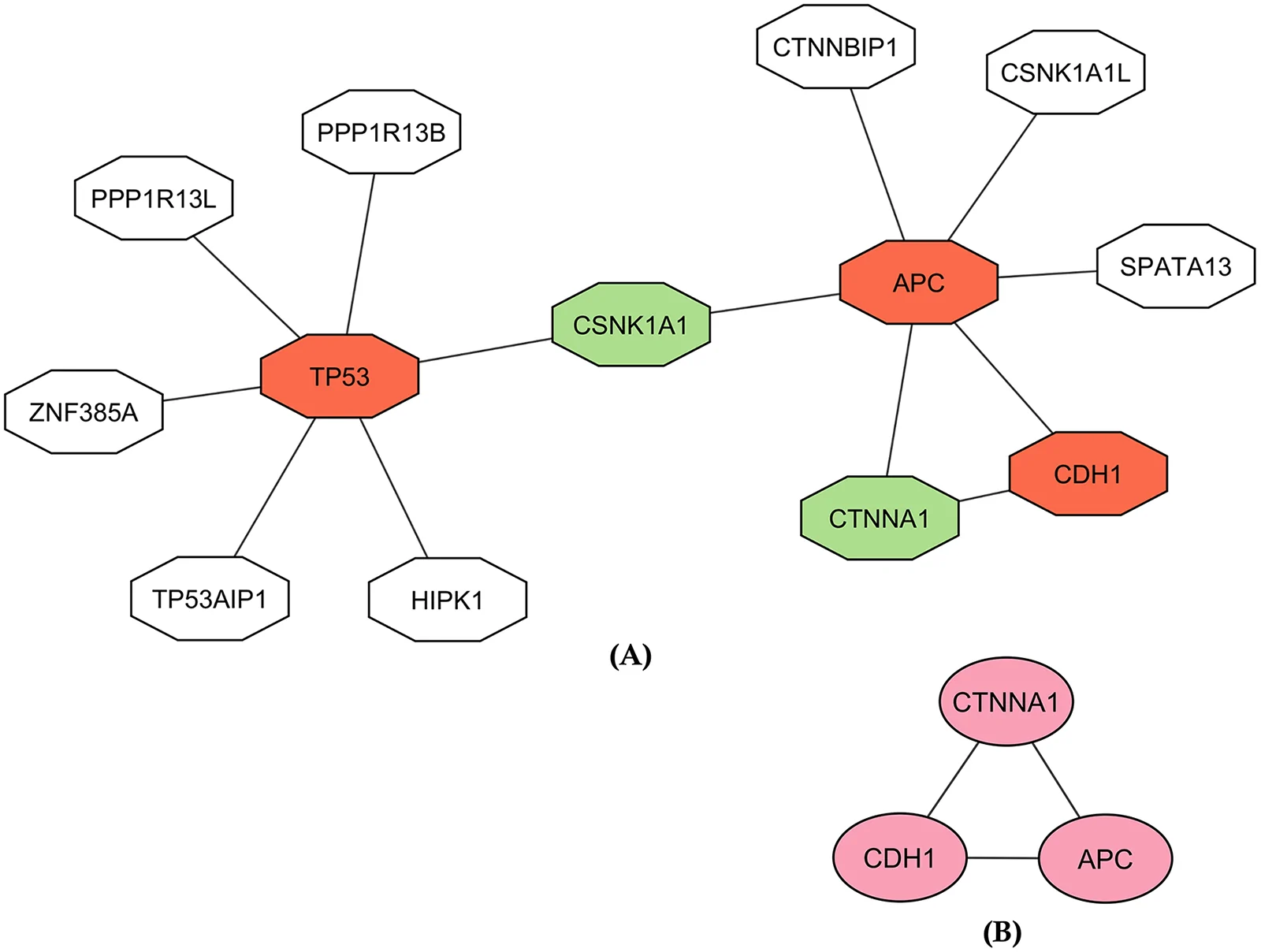
Protein-Protein interaction networks. **(A)** Whole network with the first shell of interactors, and **(B)** the protein cluster that may function together. Red Box: Tumor Suppressor Proteins and Green Box: Interconnected Nodes.

We observed a strong correlation between TP53, APC, and CDH1 proteins and the GC pathway by analyzing protein-protein interactions using the STRING server. This analysis revealed CSNK1A1 as a common interacting protein between TP53 and APC, while CTNNA1 is linked to APC and CDH1. The identification of CDH1, APC, and CTNNA1 as a distinct cluster of proteins further suggested that APC and CDH1 may exert their tumor suppressor functions via distinct mechanisms compared to TP53, which we have already evaluated at the beginning of our study (Fig 4). Fig 13 represents the whole network and protein cluster. Therefore, this network and the identified protein cluster are highly susceptible to disruption by the identified missense SNPs. Further cancer hallmarks enrichment analysis indicated significant involvement of TP53, CDH1, and APC in multiple cancer hallmarks, including sustaining proliferative signaling, evading growth suppressors, genome instability, resisting cell death, tissue invasion and metastasis, and tumor-promoting inflammation (FDR-adjusted p-values <0.05). Only TP53 showed involvement in additional hallmarks such as evading immune destruction and sustained angiogenesis (Table 5 and S1 Fig).

**Table 5:** Proteins involved in Cancer Hallmarks.

Terms	FDR-adjusted P-Value	Genes
Sustaining Proliferative Signaling	0.016897	CDH1, APC, TP53
Genome Instability	0.012678	APC, TP53
Evading Growth Suppressors	0.015035	CDH1, APC, TP53
Evading Immune Destruction	0.150911	TP53
Sustained Angiogenesis	0.150911	TP53
Tissue Invasion And Metastasis	0.012678	CDH1, APC, TP53
Tumor-Promoting Inflammation	0.012678	CDH1, TP53
Resisting Cell Death	0.012678	CDH1, APC, TP53
Reprogramming Energy Metabolism	0.012678	CDH1, TP53
Replicative Immortality	0.012678	APC, TP53

### 3.16. MicroRNAs and transcription factor-mediated gene regulatory networks

**Fig 14:**
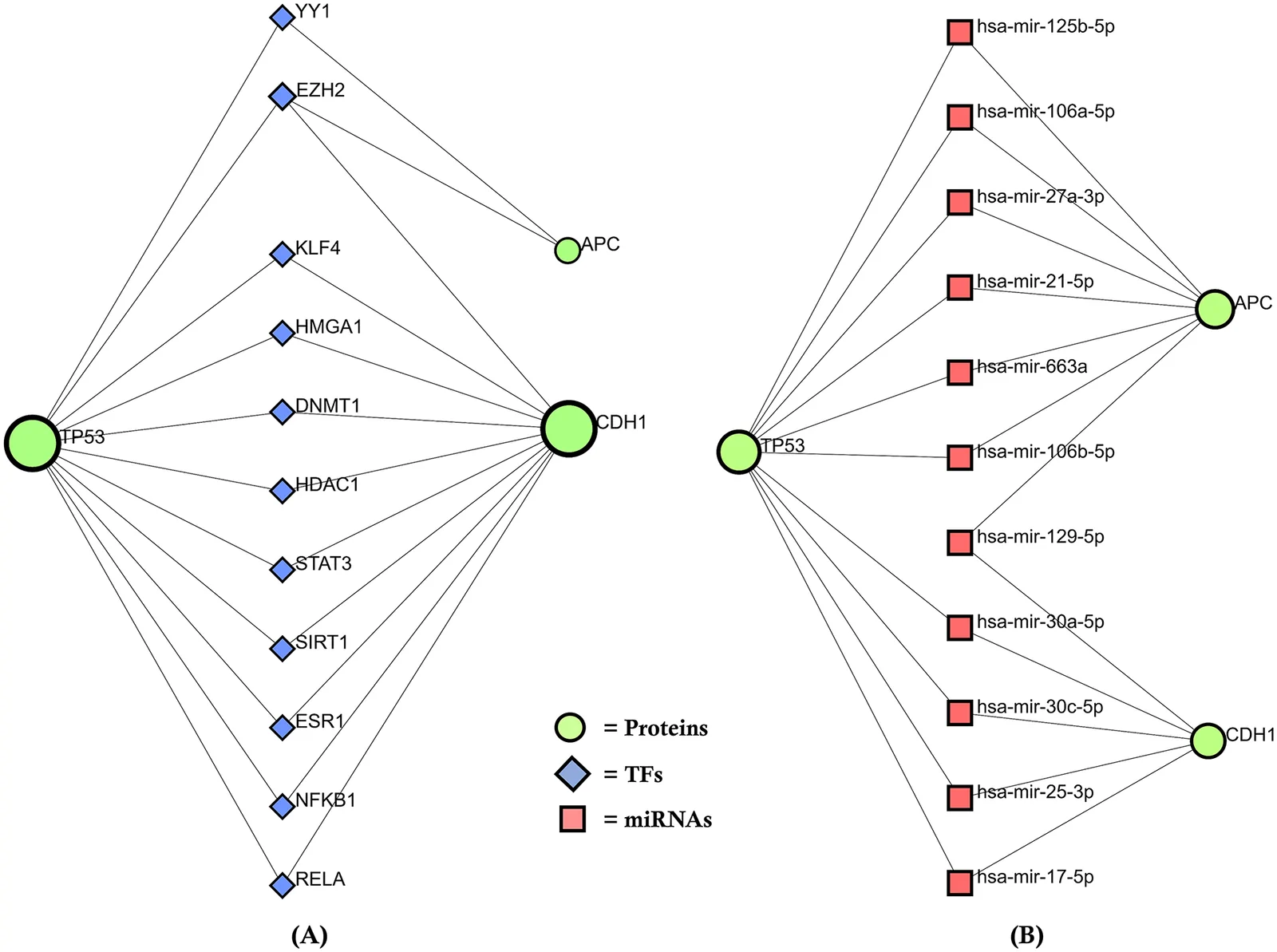
Gene regulatory networks of tumor suppressor proteins. **(A)** Gene-TFs network and **(B)** Gene-miRNAs network.

We elucidated the intricate gene regulatory networks involving tumor suppressor proteins TP53, APC, and CDH1, highlighting their potential interference in GC development. Our findings reveal that *TP53*, *APC*, and *CDH1* interact with various transcription factors and microRNAs, suggesting a complex interplay that may regulate critical cellular processes such as apoptosis, cell cycle control, and epithelial integrity. EZH2 was a common TF among TP53, APC, and CDH1. On the other hand, HMGA1, DNMT1, HDAC1, STAT3, SIRT1, ESR1, NFKB1, RELA were common between TP53 and CDH1. Only YY1 was common between TP53 and APC. Additionally, only miRNA hsa-miR-129-5p was common between TP53, APC, and CDH1. The gene regulatory network has been presented in Fig 14.

### 3.17. Altered gene expression of oncogenes

**Fig 15:**
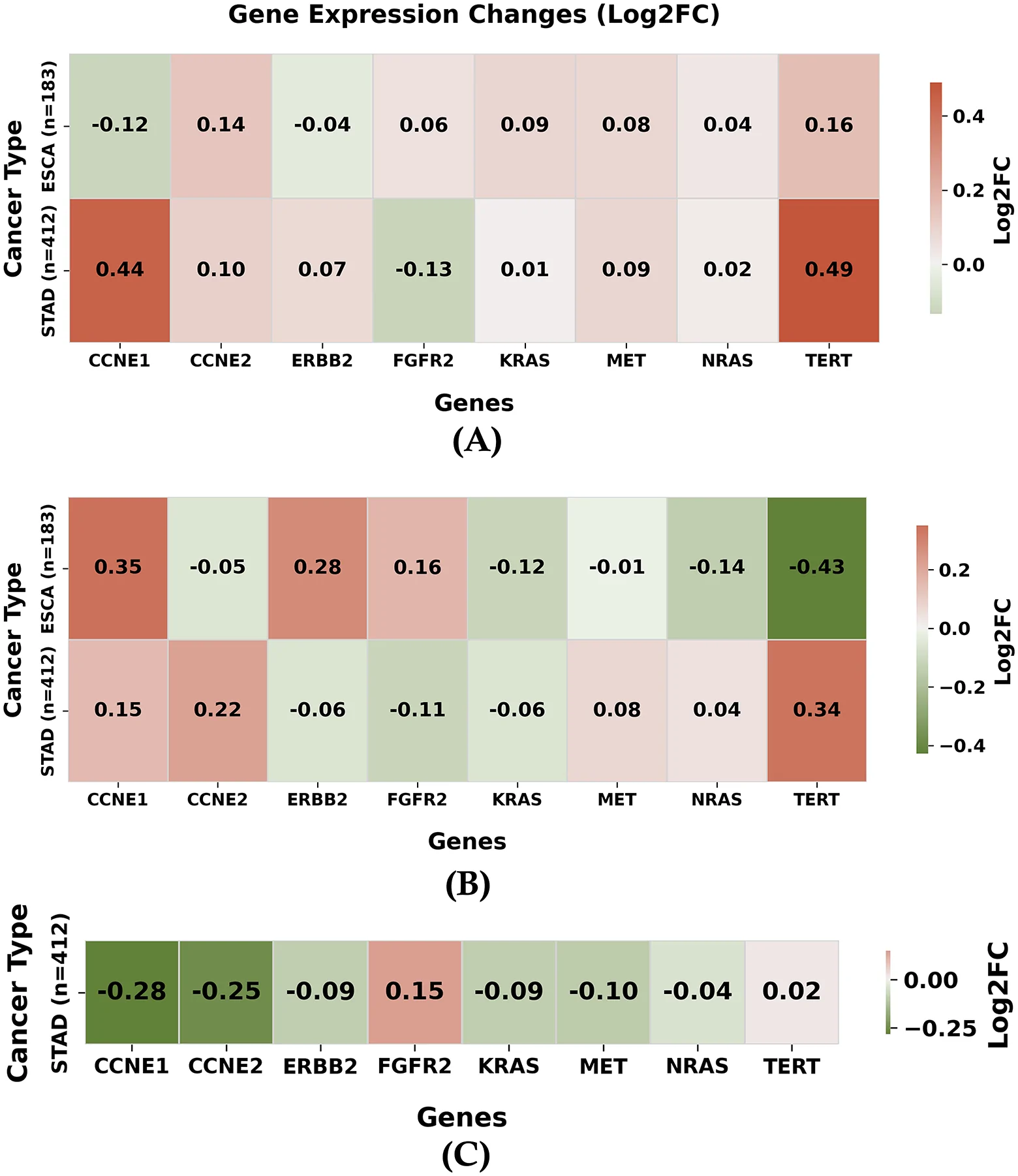
Changes in gene expression of oncogenes in the GC pathway (*TERT*, *ERBB2*, *KRAS*, *NRAS*, *CCNE1*, *CCNE2*, *MET*, and *FGFR2*) due to the mutational impact on TP53, APC, and CDH1. **(A)** TP53, **(B)** APC, and **(C)** CDH1. GC falls under ESCA: Esophageal carcinoma, and STAD: Stomach Adenocarcinoma.

Identifying gene expression alterations in oncogenes is crucial for understanding the molecular mechanisms underlying gastric carcinogenesis and developing targeted therapeutic approaches. Our analysis revealed significant changes in the expression profiles of key oncogenes within the GC pathway following mutations in tumor suppressor genes TP53, APC, and CDH1. Specifically, the oncogenes *TERT*, *ERBB2*, *KRAS*, *NRAS*, *CCNE1*, *CCNE2*, *MET*, and *FGFR2* exhibited abnormally elevated expression levels in tumor samples harboring these mutations compared to wild-type controls (Fig 15). The FDR-adjusted p-value < 0.05 was considered significant. In the case of TP53 mutation, the expression of CCNE1 (log2FC: 0.44) and TERT (log2FC: 0.49) was more significantly altered (upregulated). The mutation in APC altered the gene expression of CCNE1 (log2FC: 0.35), CCNE2 (log2FC: 0.22), ERBB2 (log2FC: 0.28), FGFR2 (log2FC: 0.16), and TERT (log2FC: 0.34). Finally, the mutation in CDH1 dysregulated (upregulated) the expression of FGFR2 (log2FC: 0.15).

## 4. Discussion

Gastric cancer (GC) is one of the leading causes of cancer-related mortality worldwide, and understanding the genetic factors and their molecular mechanisms contributing to its pathogenesis is crucial [71,72]. Our study identified 36 missense SNPs and 60 nonsense SNPs as oncogenic that were found in GC patients validated from the cBioPortal database, which includes cancer-specific evidence from published literature [28,62]. Surprisingly, our study did not observe any pathogenic and oncogenic frameshift, splice site, ncRNA, and UTR variants linked to GC pathogenesis.

We employed a deep learning-based graph neural network model to prioritize critical genes exhibiting both dysregulated expression and high mutation propensity in GC. Our model demonstrated exceptional performance across all datasets, with low mean square error (MSE: 0.00482 ± 0.00023 to 0.07108 ± 0.00437), strong goodness-of-fit metrics (R²: 0.85098 ± 0.01903 to 0.85899 ± 0.01987), and high discriminative power (AUC-ROC: 0.93095 ± 0.01758 to 0.93309 ± 0.00725). The GCN model is aligned with previous studies to prioritize central hubs based on classification and regression studies [73,74]. However, performance metrics (AUC, R²) reflect internal consistency and ranking capability, and should be interpreted as a gene prioritization model. Among the identified 3,741 differentially expressed genes and 10,981 mutation-prone genes, 1,886 genes (50.4% of DEGs) exhibited both characteristics. The analysis of the graph neural network revealed TP53 as the most prominent hub gene with the highest mutation frequency (47.6%), degree centrality (0.239), betweenness centrality (0.081), and closeness centrality (0.521), followed by ERBB2 (8.8%), CDH1 (8.2%), and APC (6.8%). Importantly, 17 of the top 20 identified genes were confirmed to be present in established GC pathways, with both the importance and predicted scores exceeding 0.83 for all genes. However, both the importance score and predicted score are computational findings derived from biological data, and therefore are not suitable for the estimation of a direct biological effect. Functional analysis of the KEGG database further revealed that TP53, CDH1, and APC function as key tumor suppressor proteins within the GC pathway, with both APC and CDH1 inhibiting beta-catenin to prevent uncontrolled proliferation, while TP53 activates in response to DNA damage to inhibit proliferation, increase survival, and mitigate genomic instability, making these three proteins the guardian of GC prevention and therefore chosen for further investigation in GC pathogenesis.

In the beginning, we identified 216 clinically significant missense SNPs in TP53, 19 in APC, and 22 in CDH1 that may contribute to GC pathogenesis. A substantial number of these SNPs (54 in *TP53*, 9 in *APC*, and 11 in *CDH1*) were predicted to be highly deleterious, decreasing protein structural stability and altering the 3D structure, and oncogenic, by multiple computational tools. For assessing the deleterious impact of missense SNPs in the TP53, APC, and CDH1 proteins, we estimated the evolutionary conservation profile of all amino acid positions in proteins via the ConSurf web server [56]. This server predicted that all of these missense SNPs are buried structural residues with a conservation score of mostly 7–9, indicating an extremely strong conservation. Furthermore, six sites (132, 175, 196, 213, 248, 273) in TP53 and one (141) in APC were methylated. On the other hand, two (125, 240) sites in TP53 were phosphorylated. The presence of potentially damaging SNPs at the post-translational modification sites could lead to structural consequences [75]. The missense SNPs (K132R, K132N, R175G, R175L, R196P) were present in the methylation sites of TP53, and missense SNPs (T125R, T125K, S240R, S240G) were present in phosphorylation sites. Hence, these particular missense SNPs present in methylation and phosphorylation sites could be methylated or phosphorylated and thus may disrupt gene expression of tumor suppressor proteins TP53 and APC. The cBioPortal validation of SNPs confirmed 35 SNPs in TP53 and 1 in CDH1 as clinically significant in GC patients that destabilize the GC pathway, increase GC risk, and might interfere with patient survival (Fig 9) [28]. This cBioPortal validation was completely different from the initial gene validation based on mutation frequency score, as in this case, all the variants themselves were validated from the database.

Afterward, biophysical energetics analyses of TP53, CDH1, and APC revealed distinct mutation-specific alterations. While titration curves showed subtle changes in charge distributions (TP53 T125R: pI 8.6 to 8.9; CDH1 D254Y: maintained pI 4.3; APC R141S: pI 5.5 to 5.3), energetic patterns demonstrated more significant effects. CDH1 D254Y exhibited a dramatic potential energy spike (+711.939 kcal/mol) at residues 178–183, TP53 T125R showed possibly altered interaction energies, and APC R141S displayed localized deviations while maintaining wild-type-like interaction energies (−300 to +100 kcal/mol). These results indicate that mutations can preserve global protein characteristics while inducing local perturbations. However, this is a comparative and computational prediction only, and hence, additional experimental studies are necessary to validate the hydropathy and energy difference impact on protein function.

Furthermore, unsupervised machine learning analysis revealed mutation-specific conformational changes at the atomic level of the region of tumor suppressor domains. TP53’s T125R mutation altered atomic associations in the first three cluster groups, while CDH1’s D254Y and APC’s R141S mutations disrupted Clusters 3 and 4, respectively. TP53 and APC showed alterations in 13 crucial amino acids, whereas CDH1 exhibited broader changes across 50 amino acids. While TP53’s disruptions were immediately apparent, CDH1 and APC variations emerged only in higher cluster groups (5 and 8), indicating that hierarchical mutation could have an impact on protein structure. However, references to silhouette scores or other experimental validation methods are required to drive any definitive conclusion.

Subsequently, our analysis and further validation study with variations other than missense mutations revealed 60 nonsense SNPs that were also confirmed from the cBioPortal database, directly linked to GC pathogenesis. Besides these observations, all three tumor suppressor genes were found to be significantly upregulated in GC-developing tissues compared to normal tissues. Survival analysis using the log-rank test revealed a statistically significant association between elevated expression of these genes and poorer patient survival (p < 0.05 for all three genes; p = 3.6e-08 for TP53, p = 7e-05 for CDH1, and p = 0.0081 for APC). These correlative outcomes suggest that increased expression of TP53, CDH1, and APC may contribute to the progression and poor prognosis of GC, potentially serving as prognostic biomarkers (Fig 12). Our study further suggested that pathogenic variations within these genes could modify protein expression and further influence tumor behavior and patient survival.

The identified pathogenic variations potentially disrupt protein-protein interactions within the GC pathway, contributing to the involvement of cancer hallmarks such as sustained proliferative signaling, evading growth suppressors, and resisting cell death. These findings provide molecular insights into how specific genetic variations may compromise tumor suppressor function and contribute to GC development. Additionally, our study revealed CSNK1A1 and CTNNA1 as crucial connecting nodes. By knowing cancer hallmarks and interacting nodes, targeted therapeutics development is possible against specific conditions that are responsible for cancer development [76].

In addition, transcription factors and miRNAs interfered with the regulation of TP53, APC, and CDH1. The transcription factor EZH2 was connected to TP53, APC, and CDH1. While YY1 was connected to TP53 and APC. Other TFs (KLF4, HMGA1, DNMT1. HDAC1, STAT3, SIRT1, ESR1, NFKB1, RELA) were connected to both TP53 and CDH1. On the other hand, only microRNA connected to TP53, APC, and CDH1 was has-mir-129-5p. MicroRNAs (has-mir-125b-5p, has-mir-106a-5p, has-mir-27a-3p, has-mir-21-5p, has-mir-663a, has-mir-106b-5p) were connected to both TP53 and APC and microRNAs (has-mir-30a-5p, has-mir-30c-5p, has-mir-25-3p, has-mir-17-5p) were connected to TP53 and CDH1. These interconnections between the gene-TFs network suggested that tumor suppressor genes of the GC pathway are regulated by these TFs, and the gene-miRNAs network suggested tumor suppressor genes may be inhibited by these miRNAs, which again provided crucial insights into the molecular mechanism behind GC pathogenesis. Previous studies identified the involvement of both EZH2 and has-mir-129-5p [77,78]; however, their association with all three critical tumor suppressor proteins has not been assessed yet. Hence, the potential structural or functional disruption of TP53, CDH1, or APC by genetic variants could alter the gene regulation in GC suppression and, consequently, might imbalance the GC pathogenesis prevention mechanism.

Moreover, the dysregulation of multiple oncogenes (TERT, CCNE1, CCNE2, MET, and FGFR2) following mutations in key tumor suppressor genes (TP53, APC, and CDH1) was observed, which suggests potential for developing combination therapies that target these interconnected oncogenic pathways simultaneously as a more effective therapeutic strategy than single-target approaches in treating GC [79].

**Fig 16:**
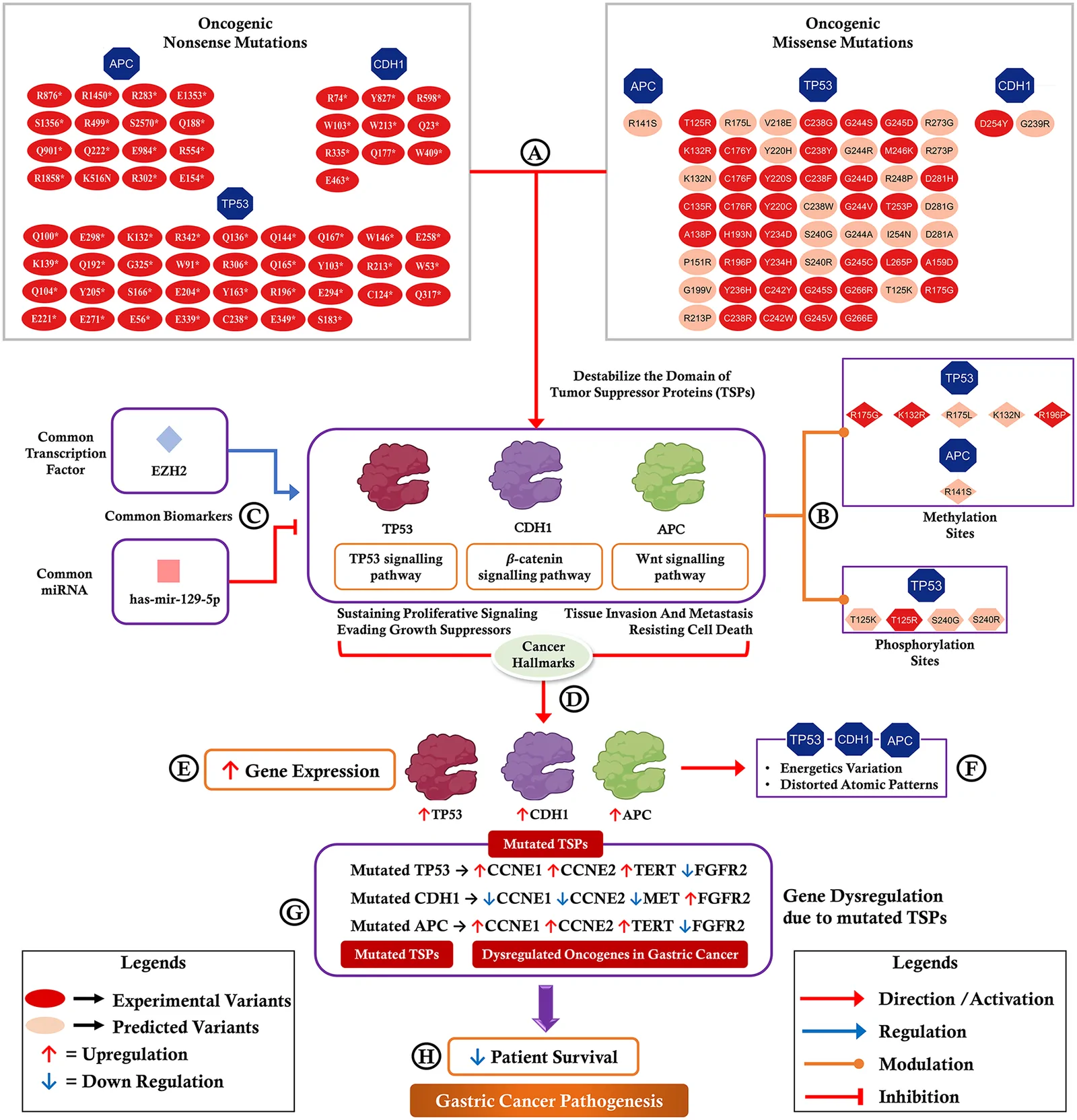
Proposed mechanistic insights into the impact of genetic variants on tumor suppressor proteins in GC pathogenesis. **(A)** Oncogenic missense and nonsense mutations on TP53, CDH1, and APC significantly compromise the structural integrity of tumor suppressor protein domains, including **(B)** the modulation of functionally critical methylation and phosphorylation sites. **(C)** These destabilizations are aided through multiple molecular interferences involving transcription factors, including EZH2, miRNA regulation, including has-miR-129-5p, leading to altered signaling pathway regulation and exhibiting cancer hallmarks. **(D)** Altered signaling pathways exhibiting cancer hallmarks additionally lead to **(E)** the dysregulation (overexpression) of tumor suppressor proteins, and **(F)** altered energetic profiles of amino acids, with distorted atomic patterns. **(G)** Subsequently, the resulting mutations cause overexpression of TP53 and APC, further leading to upregulation of CCNE1, CCNE2, and TERT, and downregulation of oncogene FGFR2. In the case of CDH1, the overexpressed CDH1 causes downregulation of CCNE1, CCNE2, MET, and upregulation of FGFR2. **(H)** As a consequence, the functional impairment of these tumor suppressor proteins contributes to GC pathogenesis and is associated with diminished patient survival.

The individual investigation of the role of genetic variations on TP53, CDH1, and APC in GC has been reported in previous studies [80–82]. However, our study expands on this by comprehensively analyzing clinically significant pathogenic genetic variations in all three genes simultaneously, integrating computational predictions of genes and validation of genetic variants using the cBioPortal database [28]. Moreover, our study provides a more holistic view by demonstrating their interconnectedness within the GC pathway and the potential synergistic effects of disrupting this pathway. The identification of molecular interferences, together with specific pathogenic variants disrupting protein structure and function, added novel insights beyond previous studies (Fig 16).

Although our study identified clinically significant oncogenic genetic variations that have evidence of disease progression, our study is limited to pathogenic, clinically significant variations and ignores genetic variations with no evidence of disease pathogenesis. Hence, our analysis might miss novel variants that are currently not assessed for their pathogenicity. Moreover, though the GCN prioritization framework minimizes circular inference through non-linear network propagation, the composite propensity score is partly derived from input features, which may introduce potential circularity that necessitates cautious interpretation.” In addition, the 0.5 sigmoid-normalized threshold for binarizing propensity scores was adopted as a practical prioritization boundary rather than a biologically optimized cutoff; hence, it may not optimally discriminate pathogenic candidates across all biological contexts. Since the graph neural network was trained to predict a composite propensity score derived from gene expression, network topology, and pathway data, not an independent clinical outcome, the reported performance metrics reflect the model’s ability to prioritize biologically relevant candidates within this framework, rather than true clinical predictive or prognostic accuracy. Subsequently, the use of multiple pathogenicity prediction tools enhances variant prioritization confidence; however, potential algorithmic dependencies and shared training databases among these tools may introduce correlated predictions, which could overestimate the robustness of variant classification. Furthermore, survival analyses were conducted without covariate adjustment and effect size reporting due to the constraints of publicly available datasets. Moreover, our validation of genetic variants using cBioPortal included evidence from published literature, potentially overlooking variants in underrepresented populations [28,83,84]. Additionally, the basis of our study is hypothesis-driven, with multi-layer computational analysis requiring further experimental studies for a definitive conclusion.

Despite these limitations, our study provides a comprehensive systems-level and potential mechanistic genes and variants prioritization framework, particularly for clinically significant pathogenic genetic variations in TP53, CDH1, and APC simultaneously. Moreover, this study offers a more holistic view by demonstrating the interconnectedness of genetic variations and molecular components within the GC pathway and the potential synergistic effects of disrupting this pathway, unlike any previous traditional network-based analysis [85]. Additionally, the identification of common TFs and miRNAs linked to tumor suppressor proteins in GC pathways may serve as biomarkers for the effective management of GC. Therefore, future research should prioritize and investigate the combinatorial approach specifically targeting the identified oncogenic pathways by providing more effective, tailored, and personalized therapeutic strategies that can significantly improve patient outcomes and survival rates in GC.

## 5. Conclusion

Gastric cancer (GC) is the fourth leading cause of cancer-related mortality worldwide, and therefore, understanding the genetic factors and their molecular mechanisms contributing to its pathogenesis may aid current insights into new avenues for gastric cancer progression and prevention processes. Our computational study unveils critical destabilization mechanisms in the gastric cancer pathway driven by genetic mutations in key tumor suppressor proteins TP53, CDH1, and APC that harbor clinically significant oncogenic variants. Our novel multi-tiered approach, combining conventional approaches with deep learning-based graph neural network modeling, biophysical energetics analysis, and unsupervised machine learning, revealed oncogenic mutations that disrupt the structure-function relationship of proteins through complex molecular interferences, including altered methylation and phosphorylation sites. Importantly, we demonstrated how these mutations trigger cascading effects on oncogene expression (TERT, CCNE1, CCNE2, MET, and FGFR2) and identified crucial regulatory elements (EZH2 transcription factor and has-miR-129-5p) that can affect all three tumor suppressors (TP53, CDH, and APC) simultaneously. While our analysis focused exclusively on pathogenic variants with established clinical significance, it may have missed novel pathogenic mutations. Hence, this computational prioritization study makes a valuable contribution by providing a systems-level understanding of mutation-induced pathway destabilization in gastric cancer. However, due to the Graph Neural Network being trained on a composite propensity score rather than an independent clinical outcome, its performance metrics reflect candidate prioritization within this framework instead of true clinical predictive accuracy. Therefore, future research should explore combinatorial therapeutic approaches targeting the interconnected oncogenic pathways, alongside developing diagnostic panels based on the mutation patterns and molecular interferences revealed to advance personalized medicine approaches for improved gastric cancer management and patient outcomes. Ultimately, this research contributes a sophisticated computational framework that bridges genetic variation with molecular mechanisms by prioritizing molecular components and variants for further precision oncology and targeted GC management.

## Supporting information

S1 Fig Cancer Hallmarks of Tumor Suppressor Proteins were significant, indicated by adjusted p-value <0.05. (DOCX)

S2 Fig Titration curve analysis to enable direct comparison of ionization behaviors. Comparison of TP53, CDH1, and APC wild-type and mutant types (A), (B), and (C), respectively.
(DOCX)

S1 File Genes utilized for the model development. (XLSX)

S2 File Detailed Model performance metrics. (XLSX)

S1 Table Differentially expressed genes involved in Gastric cancer pathogenesis. (XLSX)

S2 Table Frequency of mutation-prone genes in clinical samples of Gastric cancer patients from the cBioPortal database. (XLSX)

S3 Table List of common genes between differentially expressed genes and mutation-prone genes. (XLSX)

S4 Table Protein-protein interaction network of common genes between differentially expressed genes and mutation-prone genes. (XLSX)

S5 Table Gastric cancer pathway genes from the KEGG database. (XLSX)

S6 Table Estimated Protein Energies before minimization and after minimization. (DOCX)

S7 Table Common Genetic Variants of TP53 Genes from ClinVar and cBioportal Database. (XLSX)

S8 Table Genetic Variants of the CDH1 Gene from ClinVar Database. (XLSX)

S9 Table Genetic Variants of APC Genes from ClinVar Database. (XLSX)

S10 Table Atomic content of wild-type and mutant-type. (XLSX)

S11 Table Covariate analysis of survival in different stages of cancer. (DOCX)
