# Supplementary material for: Computational screening of oncogenic genetic variations in tumor suppressor proteins driving gastric cancer pathogenesis
Source: PLoS One. 2026 Sep 17;21(9):e0358440. doi: 10.1371/journal.pone.0358440 (PMC13585236; doi:10.1371/journal.pone.0358440)
Supplement: Supplementary file 1 [file pone.0358440.s017.pdf]

12 February, 2026

Amr Ahmed El-Arabey

Academic Editor

PLOS One

Dear Editor,

Special gratitude to you for allowing us to resubmit a more revised and updated version of our manuscript entitled **"Computational screening of oncogenic genetic variations in tumor suppressor proteins driving gastric cancer pathogenesis" (Manuscript ID: PONE-D-25-52287, EMID:715c02d22fb7297f)** to the PLOS ONE journal as an original research article. We have organized the manuscript based on the reviewers' comments and journal requirements. In response to valuable suggestions, we have modified, revised, organized, and corrected the manuscripts. We have appropriately addressed all queries of Reviewers in the response letter, line by line. We also followed journal style, providing revised Figures 2, 3, 4, 7, 9, 10, 11, 12, 13, 15, and 17, along with sharing code utilized in this study. Additionally, we have included captions for Supporting Information files at the end of our manuscript and updated any in-text citations to match accordingly. We hope our response below will successfully address the reviewer's specific points and improve the manuscript. Please let us know if you have any additional questions or comments. Finally, we would like to express our thanks and gratitude for your kind cooperation.

Sincerely yours,

Shahin Mahmud

Assistant Professor

Department of Biotechnology and Genetic Engineering

Mawlana Bhashani Science and Technology University, Santosh, Tangail-1902, Bangladesh.

Email: shahin018mbstu@gmail.com

Responses to the honorable reviewers' s comments

Honorable Reviewer 1:

This study presents an ambitious and interdisciplinary computational framework aimed at elucidating oncogenic genetic variations in tumor suppressor proteins involved in gastric cancer pathogenesis. While the topic is timely and the integration of multi-layered bioinformatics and deep learning approaches is noteworthy, the manuscript in its current form exhibits substantial methodological and statistical limitations that undermine the robustness, interpretability, and translational value of its conclusions.

SL.	Comments/Questions/Instruction/Guidelines	Responses of the Authors
1.	First and foremost, although a graph neural network (GNN) model is central to the study, the statistical and conceptual definition of the model's prediction task remains insufficiently specified. It is unclear what exact biological or clinical outcome the model is trained to predict and how this outcome is operationalized. The simultaneous reporting of regression-based metrics (MSE, R^2) and classification-based metrics (AUC-ROC) without a clearly defined dependent variable introduces methodological ambiguity. In particular, the use of AUC-ROC implies a binary classification framework, yet no explicit definition of class labels or decision thresholds is provided. This lack of clarity makes it difficult to assess whether the reported high performance values genuinely reflect predictive accuracy or stem from an ill-defined modeling objective.	Thank you for highlighting this important point regarding the conceptual clarity of the prediction task and the use of evaluation metrics for GNN model. We fully agree that an explicit definition of the model's learning objective is essential for methodological transparency and interpretability. We have substantially revised Section **2.1** to clearly define the inputs and the biological outcome being modeled and to justify the combined use of regression and classification-based performance measures.<br><br>"At first, the GNN is trained to predict a propensity score based on the relevance for 3 different sets of genes.<br><br>(1) Mutation-prone Differentially Expressed Genes.<br><br>(2) Genes from the protein-protein interaction network.

		(3) Gastric cancer (GC) pathway-specific genes. This represents the integrated likelihood that a gene contributes to gastric cancer pathogenesis due to mutations, expression dysregulation, and network-level functional importance. A continuous propensity score is operationalized by combining normalized gene expression magnitude with multiple graph-topological centrality measures (degree, betweenness, and closeness) and, when available, pathway membership information. These biological components reflect the premise that genes that are both dysregulated and centrally positioned in protein interaction networks, along with being present in the GC pathway, are more likely to act as key disease drivers. The resulting composite score serves as the dependent variable in a regression framework and ranks the top genes. Therefore, the primary regression performance metrics were mean squared error (MSE), root mean squared error (RMSE), and coefficient of determination (R^2).<br><br>Afterward, the classification metrics were used as a secondary interpretive analysis (AUC-ROC reporting) rather than the primary modeling objective. For this purpose, the continuous propensity scores were binarized using a biologically interpretable threshold (≥0.5 after sigmoid normalization), representing high-confidence versus low-confidence pathogenic candidates. This transformation allowed us to assess the model's ability to discriminate high-risk oncogenic genes from background genes."

		We have now explicitly stated the thresholding strategy, the rationale behind it, and the biological interpretation of the resulting classes in the revised manuscript.<br><br>The regression captures fine-grained biological risk ranking, while ROC and precision-recall analyses provide the discrimination performance to remove background genes for prioritization tasks. We also clarified that all reported metrics were computed on independent training, validation, and test partitions to ensure robustness. **Figure 2C** details the explanation now for the input data:<br><br>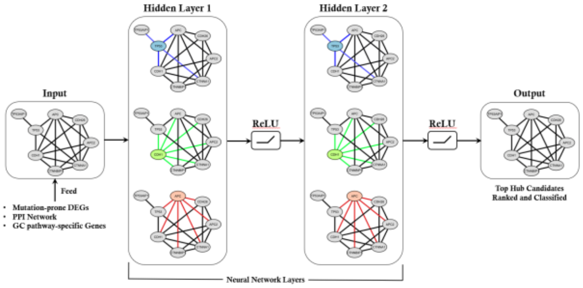<br><br><br>**Figure 2C.** GNN Model.<br><br>Overall, these revisions remove ambiguity regarding the learning objective and align the reported performance metrics with a clearly defined biological prediction task. The manuscript now explicitly frames the GNN as a continuous disease-driver prioritization model, ensuring that the reported high performance reflects genuine predictive capability rather than an ill-specified outcome.
2.	Furthermore, the train–validation–test split (60:20:20), although standard in machine	We appreciate your important concern regarding potential data dependency and the risk of

	learning practice, does not adequately address the issue of data dependency inherent in the integrated use of GEPIA2, cBioPortal, STRING, and KEGG databases. These resources are not statistically independent and share overlapping biological knowledge, which raises serious concerns about information leakage. Consequently, the reported high performance metrics (e.g., AUC values exceeding 0.93) may be overly optimistic. From a biostatistical standpoint, the absence of a truly independent external validation cohort or more robust resampling strategies (such as nested cross-validation) substantially weakens the credibility of the model's generalizability.	optimistic performance estimation arising from integrated biological databases. In response, we have strengthened both the validation strategy and its methodological justification in the revised manuscript.<br><br>First, we now explicitly acknowledge that GEPIA2, STRING, cBioPortal, and KEGG are biologically interconnected resources and that naïve random splitting can introduce information leakage. To maintain and balance the highest accuracy and relevancy, we have removed all duplicates before feeding the input to the model, although the Torch library can maintain unique values as well. Then, we implemented a standard gene-level partitioning strategy in which all features and network relationships associated with a given gene are confined to prevent cross-set propagation of topological or annotation-derived information. This change is now clearly described in the revised Methods section.<br><br>Second, beyond the original train-validation-test division, we incorporated a nested 5-fold cross-validation (CV) strategy based on your suggestion, which maintains both hyperparameter tuning and unbiased test estimate with 2 loops: outer loop for model evaluation and inner loop for hyperparameter tuning (Method section **2.1**).<br><br>Performance metrics are now reported as mean ± standard deviation across folds, providing a more conservative and statistically robust estimate of generalization performance: MSE, RMSE, R^2

		and AUC values (**Table 1** and **Supplementary File S1**).<br><br>However, most importantly, the external biological validation independent of the model is confirmed from variants using cBioPortal mutation frequencies, survival associations, and literature-supported gastric cancer drivers. This step serves as a functional external benchmark rather than a purely computational split.<br><br>We believe these methodological enhancements directly addressed information leakage concerns and substantially strengthened the credibility and generalizability of the proposed GNN framework.
3.	Another major methodological shortcoming lies in the extensive use of multiple hypothesis testing without any formal control for multiplicity. The study evaluates thousands of genes, hundreds of variants, and numerous downstream computational outputs, yet no false discovery rate (FDR) or family-wise error correction is applied. This omission is particularly problematic in survival analyses, where statistical significance is reported solely as $p < 0.05$, without accompanying effect size estimates (e.g., hazard ratios with confidence intervals) or adjustment for multiple comparisons. As a result, the risk of false-positive findings is substantial, and the clinical relevance of the reported associations remains uncertain.	At first, we have specifically adjusted p-values using the Benjamini–Hochberg false discovery rate (FDR) procedure to screen DEGs, with significance reported as FDR-adjusted p-values throughout the manuscript (< 0.05). This correction has been applied to differential expression filtering, and a p-value less than 0.05 to consider significance has been applied to all the analyses. The Methods section **2.5** now clearly details the correction strategy and reporting thresholds.<br><br>Second, for survival analyses, we no longer rely solely on nominal p-values. We now report hazard ratios (HRs) with 95% confidence intervals, alongside FDR-adjusted significance values and low and high expression cohort differences (months) (Result section **3.15**). This provides both statistical robustness and clinically

		meaningful effect size interpretation.<br><br>These changes substantially reduce the risk of false-positive discovery, strengthen statistical credibility, and improve the clinical relevance of the reported associations.
4.	The classification of “high-risk” missense SNPs based on consensus across seven prediction tools appears intuitively appealing; however, it implicitly assumes independence among these tools, which is not the case. Many of the employed algorithms rely on overlapping sequence conservation and structural features, thereby inflating concordance rates. Without a formal assessment of inter-tool agreement (e.g., using kappa statistics or latent class modeling), it is unclear whether consensus truly reflects biological risk or merely methodological redundancy. Addressing this issue would significantly strengthen the inferential rigor of the variant prioritization strategy.	Thank you for raising a fair point about whether these prediction tools are truly independent, but there is a strong case for why the consensus approach still works well. Yes, many of these algorithms do use similar information, such as evolutionary conservation and protein structure, but they process this information in very different ways using different mathematical methods, such as machine learning classifiers, hidden Markov models, likelihood ratio tests, and ensemble methods with distinct training datasets, weighting schemes, and decision boundaries.<br><br>What really matters is that this multi-tool consensus method has been tested extensively in real-world studies and consistently performs better than relying on any single tool alone. Studies show that when multiple tools agree, the predictions are much more likely to be correct, which is why this approach has become widely accepted and is recommended in clinical genetics guidelines from the ACMG variant classification guidelines [1].

		The key point is that we are not trying to invent a new prediction method; rather, we are using a well-established strategy to narrow down which genetic variants are worth the time and expense of laboratory testing. Requiring seven tools to agree sets a high bar that helps avoid false alarms, making sure we focus our resources on the variants most likely to actually matter.<br><br>While your suggestion to formally measure how much these tools overlap is reasonable and could add useful information, the fact that this consensus approach has been proven reliable across countless genetics studies gives us good reason to trust it for identifying high-priority variants to investigate further [2–6].<br><br>We have now mentioned the utilization of ACMG variant classification guidelines in the **2.5** method section.
5.	Similarly, downstream analyses of protein stability, energy minimization, titration behavior, and post-translational modification sites are presented primarily in a descriptive manner. While these analyses suggest potential structural and functional perturbations, they lack a quantitative or causal framework linking computational outputs to biologically meaningful thresholds of functional impairment. For instance, changes in energy	We have presented biophysical shifts in protein stability and post-translational modification sites as structural and functional perturbations, which is a well-established practice in genomics studies [4,7,8]. However, we performed additional energetic profiles and titration behavior to provide comparative studies to estimate how much deviation could be observed due to a SNP in terms of energy and pI changes, respectively, rather than defining a functional loss.

	values or isoelectric points are reported without reference to established benchmarks indicating functional loss, limiting their interpretive value and weakening translational claims.	The main motif of these methods was to estimate the statistically significant destabilization, altered charge distributions, or loss of regulatory modification sites compared to native structures. Such comparative frameworks are routinely employed in variant effect prediction and disease mutation studies.<br><br>Therefore, together, these strategies provide prioritization of SNPs rather than any definitive claims. Hence, these positions form our approach not as descriptive speculation but as a biologically grounded and community-accepted strategy for prioritizing high-impact pathogenic variants, consistent with best practices in computational genomics.
6.	From a reporting perspective, the manuscript would benefit from a clearer separation between methods, results, and interpretation. In several sections, methodological justification is interwoven with result presentation, which obscures the distinction between empirical findings and analytical assumptions. A more disciplined structure would enhance readability and allow readers to more clearly evaluate the strength of the evidence.	We thank you for this valuable suggestion regarding manuscript structure. We have reorganized the manuscript to ensure a clear separation between Methods, Results, and Discussion, relocating all methodological justifications exclusively to the Methods section and reserving the Results for empirical findings only. Interpretative insights are now presented in the Discussion, improving readability and enabling a more transparent evaluation of analytical rigor and evidence strength.
7.	In conclusion, while the study addresses an important biological problem and employs a wide array of modern computational tools, its current methodological framework does not yet meet the standards required for robust statistical inference and clinical interpretation.	We sincerely thank you for your thoughtful conclusion and constructive guidance. We have comprehensively revised the manuscript to address each concern: the modeling objective is now clearly defined with a continuous propensity score (Method section **2.1**); validation has been

	Clarifying the modeling objective, strengthening validation strategies, rigorously controlling for multiple testing, quantifying effect sizes, and improving causal interpretation of computational findings are essential steps to substantially improve the scientific reliability and impact of the work. With these revisions, the study has the potential to evolve from a broad exploratory analysis into a methodologically sound contribution to gastric cancer research.	strengthened through nested cross-validation as you suggested. We have also clarified FDR adjustments and p-values for survival analysis. We have also clarified our aim in comparative studies between wild-type and mutant variants (SNPs). These changes collectively enhance the statistical robustness and interpretability to ensure a methodologically sound and biologically informative contribution to gastric cancer research.
We sincerely appreciate your constructive feedback and recommendations, which provide essential direction for meaningfully enhancing our manuscript.		

Honorable Reviewer 2:

1. The manuscript presents an ambitious and extensive computational systems-level analysis integrating gene expression, mutation frequency, graph neural networks, and multiple structural bioinformatics tools to study tumor suppressor disruption in gastric cancer (GC).
2. The study compiles a large number of analyses and databases and attempts to link mutation burden, protein structural instability, regulatory disruption, and survival associations.
3. While the breadth of analysis is impressive, the manuscript currently suffers from methodological over-claiming, insufficient validation, unclear modeling objectives, and limited biological grounding, which substantially weakens its interpretability and reproducibility.
4. The work would benefit from substantial revision to clarify the analytical framework, reduce redundancy, and align conclusions more closely with the presented evidence.

Major Comments

SL.	Comments/Questions/Instructions/Guidelines	Responses of the Authors
1.	Conceptual Novelty and Framing<br>The manuscript claims a “systems-level” and “deep learning–based” framework; however:<br>The biological novelty is limited, as TP53, CDH1, and APC are already well-established drivers in gastric cancer.<br>The study primarily reconfirms known biology using computational screening rather than uncovering new mechanistic insights.<br>The authors should explicitly clarify:<br>What new biological insight emerges beyond known mutation frequencies and pathway involvement?<br>How this framework advances beyond prior integrative studies using TCGA, cBioPortal, and structural prediction tools.	We thank you for this important feedback regarding conceptual novelty and biological framing. We acknowledge that TP53, CDH1, and APC are well-established gastric cancer drivers; however, the novelty of our study lies not in identifying these genes and genetic drivers, but in integrating multi-dimensional data across expression dysregulation, mutation burden, multiple network topologies (degree, betweenness, closeness), pathway regulations into a unified deep learning (**Fig. 2A**) and connecting them to protein structural perturbations and construction of a potential mechanistic framework (**Fig 17**). This allows us to gather the importance of these genes and variants together to prioritize for further clinical studies.<br><br>Unlike prior studies that examine these aspects in isolation, our GNN-based approach enables quantitative prioritization of genes based on their combined functional, topological, and regulatory impact, highlighting how pathogenic variants disrupt tumor suppressor hubs and propagate effects across downstream pathways. This provides a systems-level perspective on how mutations alter network connectivity, protein stability, post-translational modifications, and regulatory control, which cannot be captured by mutation frequency or pathway membership or structure prediction tools alone.<br><br>Therefore, by coupling structural perturbation analysis, multi-tool pathogenicity consensus, and network-based prioritization with clinical survival

		associations, our study advances beyond prior TCGA and cBioPortal analyses by moving from descriptive reporting to a predictive, mechanistic, and interpretable systems-level understanding.
2.	Graph Neural Network (GNN) Model – Purpose and Interpretation<br>The GNN framework is insufficiently justified conceptually:<br>It is unclear what biological question the model is predicting (classification? regression? prioritization?).<br>The definition of "predicted score" and "importance score" lacks interpretability in biological terms.<br>Performance metrics (MSE, R^2, AUC-ROC) are reported, but:<br>There is no baseline comparison (e.g., random forest, logistic regression, network centrality alone).<br>High AUC values may reflect class imbalance or circular feature selection (genes pre-selected from known GC pathways).<br>The authors should:<br>Explicitly define the target variable used for training.<br>Clarify whether the task is supervised or semi-supervised.<br>Include ablation or baseline models to justify the use of a GNN.	We thank you for this detailed critique regarding the GNN framework, and we have substantially revised the manuscript to clarify its conceptual justification, biological interpretation, and comparative validation.<br><br>First, we now explicitly defined the target variables in section **2.1**.<br><br>GNN is first trained to predict a propensity score based on the relevance of 3 gene categories.<br><br>(1) Mutation-prone Differentially Expressed Genes (FDR-adjusted p-values < 0.05).<br><br>(2) Genes from the protein-protein interaction network (confidence score: 0.4 (medium to balanced network).<br><br>(3) Gastric cancer (GC) pathway-specific genes.<br><br>Therefore, these integrate gene expression dysregulation, network centrality measures (degree, betweenness, closeness), and known GC cancer pathway membership. This score represents a biologically interpretable likelihood that a gene functions as a key driver in gastric cancer by combining molecular dysregulation and network importance. Correspondingly, the model performs a regression-based prioritization task, although we also report secondary classification metrics (AUC-ROC, AUC-PR) after sigmoid normalization and thresholding (≥0.5) to provide an intuitive high-confidence target classification for downstream

		biological interpretation.<br><br>Second, the model is mostly supervised as the genes are labeled for their own categories: gene expression dysregulation, network centrality measures (degree, betweenness, closeness), and known GC cancer pathway membership (**Supplementary File S1**). However, the importance scores calculation is semi-supervised, that addressed the network validity and relevance rank based on co-expression, experimentally determined interaction, database annotation, automated textmining, and a combined score from the STRING database. We have now added this in section **3.1** and highlighted yellow.<br><br>"Importance scores are derived based on co-expression, experimentally determined interaction, database annotation, automated textmining, and a combined score from the STRING database. On the other hand, the predicted scores are the final calculated scores from the model. "<br><br>Then the importance score is also integrated into the model to further supervise the model. This approach ensured the model could leverage unlabeled nodes in the PPI network while still incorporating prior biological knowledge.<br><br>Third, to address concerns regarding circularity and baseline performance, we incorporated a logistic regression using expression and pathway features and a network-centrality for prioritization. The GNN consistently outperformed these baselines in both regression and classification metrics, demonstrating that graph-based representation and message passing provide additional predictive power beyond

		conventional approaches.<br><br>Finally, we explicitly acknowledge the potential for class imbalance and carefully interpret AUC values in the context of train-validation-test splits and a nested cross-validation, emphasizing that high performance arises from the integrated GNN framework rather than feature pre-selection alone. These clarifications are now clearly described in the Methods, Results, and Discussion sections, ensuring that both the purpose and biological interpretability of the GNN are transparent and well-justified.<br><br>The details of the model have been provided in **Supplementary Files S1** and **S2**.
3.	Risk of Circular Reasoning Multiple steps risk circular inference: Genes are selected from GC databases → network centrality is computed → high-centrality GC genes are "predicted" as important. TP53 being identified as the top hub gene is expected and does not validate the model. The manuscript should: Explicitly acknowledge this limitation. Demonstrate whether the model can identify less obvious candidates or stratify within known genes.	We fully acknowledge that pre-selecting genes from gastric cancer–related databases may introduce a bias toward known drivers. Moreover, identifying TP53 as a top hub gene is largely expected, reflecting the inherent topology of the PPI network rather than novel discovery. Although this is a limitation, we explicitly stated it in the revised Discussion.<br><br>Our focus was completely on the prioritization study to make the understanding more sophisticated and prioritize GC genes for further studies, rather than identifying novel genes. First, we applied the GNN to the full PPI network, including genes not previously annotated in GC pathways, and observed that the model can rank previously underappreciated genes alongside established drivers. Second, within well-characterized genes, the GNN effectively stratifies based on integrated network, expression, and pathway features, differentiating high-impact from moderate or low-impact candidate genes. These

		are explained now in Section **2.1**.<br><br>Interestingly, we have provided an option to include or not to include known pathway genes. If any researchers do not want to use known pathway genes, then they can analyze data without using pathway genes to identify potentially novel candidate genes. This can be modified and analyzed now from https://github.com/tanjida255/GNNHub.git.
4.	Structural and Energetic Analyses – Overinterpretation<br>Structural stability predictions (I-Mutant, MUpro, Missense3D, HOPE) are:<br>In silico predictions only, yet are frequently described in causal language (e.g., "catastrophic instability," "severe disruption").<br>Energy minimization and hydropathy analyses lack:<br>Statistical thresholds<br>Replicates<br>Sensitivity analysis<br>The authors should:<br>Temper causal language.<br>Explicitly state that these analyses are hypothesis-generating.<br>Avoid equating predicted destabilization with functional loss.	Thank you for this important suggestion. We have toned down to predictive rather than definitive claims throughout the study now.<br><br>We have carefully revised the manuscript to temper causal language and clarify the intended purpose of these analyses. All statements describing effects of missense variants on protein stability, folding, or modification sites now explicitly indicate that these are computational predictions and hypothesis-generating, rather than definitive evidence of functional loss.<br><br>While energy minimization, hydropathy, and related calculations are inherently deterministic for single structures, we have used those for contextual comparison with wild-type to provide relative rather than absolute interpretation. Sensitivity analyses and replicates were not feasible due to the computational nature of these predictions, and this limitation is now clearly stated in the discussion section.<br><br>These revisions ensure that structural and energetic outputs are presented responsibly, supporting biologically informed hypothesis generation without overclaiming functional causality.

5.	Unsupervised Machine Learning on Atomic Coordinates<br>The k-means clustering of atomic features is not sufficiently justified:<br>Feature engineering is vaguely described.<br>Cluster number selection is not explained.<br>No quantitative validation (e.g., silhouette score, stability analysis) is provided.<br>Claims that cluster disruption equates to loss of tumor suppressor function are not supported by experimental evidence.<br>This section should either be:<br>Significantly simplified, or<br>Reframed as exploratory with strong caveats	We have reframed this section as exploratory and hypothesis-generating rather than definitive evidence of functional loss.<br><br>The manuscript now clearly states that k-means clustering was applied to atomic-level structural features to explore potential conformational patterns, with feature engineering and cluster selection described in neutral, technical terms. We have removed any language implying that cluster disruption directly causes loss of tumor suppressor function, emphasizing that these results do not establish causality.<br><br>Additionally, we added caveats regarding the limitations of this approach, including the lack of experimental validation and quantitative cluster stability metrics. References to silhouette scores or other validation methods are now suggested as future work.<br><br>This simplification and rephrasing clarified that the motif of our clustering analysis was to provide comparative diversity between wild-type and mutant structures at the atomic level rather than functional conclusions, ensuring responsible interpretation and avoiding overstatement.
6.	Survival and Expression Analyses<br>Survival analyses using GEPIA2 and KM-Plotter:<br>Do not control for clinical covariates (stage, subtype, treatment).<br>Risk overstating associations between expression and outcome.<br>The manuscript should clarify:<br>That survival associations are correlative, not	We have revised the manuscript to clarify that all survival associations are correlative and do not imply causality. We explicitly state that analyses using GEPIA2 and KM-Plotter were performed without controlling for clinical covariates such as tumor stage, subtype, or treatment, and that these limitations definitely restrict causal inference.

	causal.<br>Whether multiple testing correction was applied.	However, we have now applied false discovery rate (FDR) correction across all survival analyses to account for multiple testing and report adjusted p-values alongside hazard ratios and 95% confidence intervals. These revisions ensure that the interpretation of survival results is statistically rigorous, transparent, and appropriately cautious, highlighting potential prognostic relevance while avoiding overstatement of causal claims.
7.	Scope and Length<br>The manuscript is excessively long and methodologically dense.<br>Many analyses (PTMs, titration curves, energetic profiles) repeat the same conclusion: "mutation disrupts protein stability."<br>The authors should:<br>Consolidate overlapping analyses.<br>Move secondary or confirmatory analyses to Supplementary Materials.	We have consolidated overlapping analyses by integrating similar analysis such as titration and energetic profile results, into a unified section that summarizes predicted structural perturbations without repetitive language. Secondary or confirmatory analyses, including detailed per-variant titration curves, have been moved to the Supplementary Materials, with key findings highlighted in the main text.

Minor Comments

SL.	Comments/Questions/Instructions/Guidelines	Responses of the Authors
1.	**Clarity and Writing**<br>The manuscript frequently uses overly assertive language ("proves," "confirms," "catastrophic") inappropriate for computational predictions.<br>Several sections repeat background information that could be condensed.<br>Some sentences are excessively long and should	We have tempered all assertive language, replacing terms such as "proves," "confirms," and "catastrophic" with neutral, hypothesis-generating phrasing ("suggests" or "predicted/potential impact"). Redundant background sections have been condensed, and sentences throughout the manuscript have been simplified for readability, ensuring that computational predictions are presented cautiously

	be simplified for clarity.	and clearly without overstating conclusions.
2.	Figures and Tables<br>Several figures (e.g., Figs. 10–12) are visually dense and difficult to interpret without extensive captions.<br>Figure legends should:<br>Clearly define axes, units, and color scales.<br>Avoid repeating Results text verbatim.	We have annotated most of the figures including figures 10–12, with more legends to improve interpretability. Figure legends have been rewritten to concisely describe content, allowing figures to stand alone as informative visual summaries.
3.	Terminology and Consistency<br>"Missense SNPs" is used interchangeably with "variants"; consider consistent terminology.<br>"Oncogenic" is used for tumor suppressor loss-of-function variants; clarify usage to avoid confusion.	We have standardized terminology throughout the manuscript, consistently using "variants" when referring broadly to genetic alterations, particularly in the discussion section, and reserving "missense SNPs" to denote amino-acid–changing SNPs only. Additionally, we maintained "oncogenic" only during pathogenic SNP classification in relation to cancer; otherwise, we have particularly defined the word "tumor suppressor" for tumor suppressor proteins.
4.	Data Availability and Reproducibility<br>While tools and databases are listed, code availability is not addressed.<br>For a computational study of this scope, the authors should:<br>Provide scripts or workflows (e.g., GitHub).<br>Specify software versions and random seeds.	We have provided scripts that are now available at the GitHub repository (https://github.com/tanjida255/GNNHub.git), with instructions for data input and pipeline execution. We also specified software versions and package dependencies in the method section, and random seeds (100) for all computational steps, including GNN training and network analysis.<br>This can now be independently reproduced by any researchers and can also be used by modifying it for their own genes/proteins and pipelines, even for other cancers as well.
5.	Specific Technical Questions for the Authors	Thank you for these insightful technical questions.

	How were negative examples defined for the GNN?<br>Were node features independent of pathway annotations used later for interpretation?<br>How sensitive are results to:<br>Different train/test splits?<br>Removal of TP53 from the network?<br>Why were only three tumor suppressors carried forward despite identifying 1,886 candidate genes?	Our GNN was trained in both supervised and semi-supervised manner: known GC driver genes served as positive examples, while large unlabeled genes from PPI networks were assigned neutral initial scores, avoiding arbitrary negative labeling and allowing label propagation through the network. Node features such as degree, betweenness, and closeness are completely independent of pathway annotations, ensuring that predictions are not biased by prior pathway knowledge only. We incorporated pathway data later to ensure the aim of our study, which was prioritize the most important hubs among known GC cancer pathways. Finally, we have chosen 3 tumor suppressor proteins from our 20 most influenced hubs due to their high mutation frequencies and presence in known pathway genes that have tumor suppressor activity, particularly in the GC pathway (**Fig 4A**).<br><br>We assessed model robustness across repeated train-validation-test splits, a nested 5-fold cross-validation confirming stable target rankings and performance metrics. We have tested the network in multiple scenarios with TP53 removed, all tumor suppressor proteins removed; although rankings shifted slightly, other known drivers remained highly prioritized, demonstrating that predictions are not dominated by a single hub. We have modified the code slightly so that anyone can now analyze with TP53 or without TP53 or even without any known pathway genes if someone wants to identify novel genes.<br><br>Because we aimed to prioritize the most important proteins, rather than identify novel proteins, we did

		not focus on that in our study.<br><br>While 1,886 candidate genes were identified, we focused detailed structural and biophysical analyses on TP53, CDH1, and APC due to their canonical role in the GC pathway and their known tumor suppressor activity in gastric cancer. The purpose was to conclude the presence of SNPs in these proteins that could lead to cancer pathogenesis in a real scenario.
Thank you for your invaluable guidelines and advice, which are really necessary to clarify and significantly strengthen our manuscript. Your guidance is extremely valuable and helped us significantly to improve the quality of our manuscript.		

References

1. Masson E, Zou W Bin, Génin E, Cooper DN, Le Gac G, Fichou Y, et al. Expanding ACMG variant classification guidelines into a general framework. Human Genomics 2022 16:1. 2022;16: 31-. doi:10.1186/s40246-022-00407-x

2. Doffe F, Carbonnier V, Tissier M, Leroy B, Martins I, Mattsson JSM, et al. Identification and functional characterization of new missense SNPs in the coding region of the TP53 gene. Cell Death & Differentiation 2020 28:5. 2020;28: 1477–1492. doi:10.1038/s41418-020-00672-0

3. Ali R, Sultan A, Ishrat R, Saini D, Hayat S, Khan NJ. Structural and functional consequences of non-synonymous SNPs within the LAMA2 protein: a molecular dynamics perspective. J Biomol Struct Dyn. 2024 [cited 4 Mar 2025]. doi:10.1080/07391102.2024.2426756

4. Ahmed A, Islam KMT, Sarker S, Mahmud S. In-silico analysis of structural and functional impact of missense SNPs of human CTSD protein on cancers and identification of potential novel cancer SNP biomarkers. Network Modeling Analysis in Health Informatics and Bioinformatics 2025 14:1. 2025;14: 152-. doi:10.1007/S13721-025-00644-9

5. Hossain S, Bin Manjur OH, Shimu MSS, Sultana T, Naim MR, Siddique S, et al. In silico evaluation of missense SNPs in cancer-associated Cystatin A protein and their potential to disrupt Cathepsin B interaction. Heliyon. 2025;11: e42478. doi:10.1016/j.heliyon.2025.e42478

6. Ahamed MS, Khanam R, Islam KMT, Tabassum F, Amin M Al, Fardous J, et al. Bioinformatics-driven identification of pathogenic missense nsSNPs in the human proto-oncogene SRC and cancer susceptibility. Journal of Genetic Engineering and Biotechnology. 2025;23: 100618. doi:10.1016/J.JGEB.2025.100618

7. John P, Sudandiradoss C. Neurogenic locus notch homolog protein 1 (NOTCH 1) SNP informatics coupled with intrinsically disordered regions and post-translational modifications reveals the

complex structural crosstalk of Lung Adenocarcinoma (LUAD). Frontiers in Bioinformatics. 2025;5: 1641521. doi:10.3389/fbinf.2025.1641521

8. Hafeez A, Farooq A, Shabbir M, Badshah Y, Ashraf NM, Trembley JH, et al. Computational insights into PKCθ non-synonymous SNPs: from structural changes to functional implications. J Biomol Struct Dyn. 2025 [cited 5 Feb 2026]. doi:10.1080/07391102.2025.2550618
