# Supplementary material for: Computational screening of oncogenic genetic variations in tumor suppressor proteins driving gastric cancer pathogenesis
Source: PLoS One. 2026 Sep 17;21(9):e0358440. doi: 10.1371/journal.pone.0358440 (PMC13585236; doi:10.1371/journal.pone.0358440)
Supplement: Supplementary file 2 [file pone.0358440.s018.pdf]

03 March, 2026

Amr Ahmed El-Arabey

Academic Editor

PLOS One

Dear Editor,

Special gratitude to you for allowing us to resubmit a more revised and updated version of our manuscript entitled **"Computational screening of oncogenic genetic variations in tumor suppressor proteins driving gastric cancer pathogenesis" (Manuscript ID: PONE-D-25-52287R1)** to the PLOS ONE journal as an original research article. We have revised the manuscript based on the reviewers' comments and journal requirements. In response to valuable suggestions, we have modified, revised, explained, and justified the manuscripts. Additionally, we have provide more clear explanation regarding model validation and performance, stability, atomic and energetic analysis, and survival and covariate analysis. We hope our response below will successfully address the reviewer's specific points and improve the manuscript. Please let us know if you have any additional questions or comments. Finally, we would like to express our thanks and gratitude for your kind cooperation.

Sincerely yours,

Shahin Mahmud

Assistant Professor

Department of Biotechnology and Genetic Engineering

Mawlana Bhashani Science and Technology University, Santosh, Tangail-1902, Bangladesh.

Email: shahin018mbstu@gmail.com

**Responses to the honorable reviewers' s comments**

Honorable Reviewer 1:

After examining the revised manuscript, it is clear that the authors attempted to respond to many comments; however, a substantial portion of the revisions remains explanatory rather than corrective, and the statistical reliability of the study has not been fully established.

SL.	Comments/Questions/Instruction/ Guidelines	Responses of the Authors
**1.**	First, the clarification regarding the GNN model target variable is not methodologically sufficient because the "propensity score" is not an observed biological outcome but a composite score constructed by the researchers. Therefore, the model does not predict a real outcome but rather relearns predefined features within the same dataset.	Thank you for your important methodological concern. We agree that the propensity score is not a directly observed biological endpoint but a composite score derived from biologically informed features. However, the intention of our framework is not to predict an external clinical phenotype; rather, it is to perform integrative, topology-aware gene prioritization under structured biological supervision.<br><br>As described, the propensity score integrates three biologically grounded gene categories: (1) mutation-prone differentially expressed genes (FDR < 0.05), (2) genes within the protein–protein interaction network (confidence ≥ 0.4), and (3) gastric cancer pathway–specific genes. The score combines multiple graph-topological centrality measures (degree, betweenness, closeness, and additional topology-derived features) and pathway membership. These components reflect a mechanistic hypothesis: genes that are dysregulated, mutation-enriched, and centrally positioned in the

		interaction network are more likely to function as gastric cancer drivers.<br><br>However, the GNN does not simply relearn this additive formula used to initialize this score. The model applies graph convolutional layers that perform neighborhood feature aggregation through weighted message passing across the PPI network. Consequently, each gene's predicted value is influenced not only by its own attributes but also by the higher-order topology and feature distribution of its interacting neighbors. This induces non-linear, context-aware transformations that differ fundamentally from the original linear weighting scheme. In other words, while the composite score provides structured supervision, the GNN learns a graph-informed representation that captures emergent network effects not explicitly encoded in the predefined formula. This is the reason the model can still capture, for example, TP53 as a top candidate, even excluding it from pathway components.<br><br>To avoid circularity, overfitting concerns, and feature re-learning within the same dataset, we implemented a full nested cross-validation pipeline (outer 5-fold, inner 5-fold) with strict train/validation/test separation at the node level. The chosen training epoch is determined exclusively within the inner loop, and performance metrics (MSE, RMSE, R^2, accuracy, precision, recall, F1, AUC-ROC, AUC-PR) are reported only

on unseen outer test folds.

The last fold (5th) during model development:

```
  Inner fold 1: best_epoch=195, best_val_loss=0.0052
  Inner fold 2: best_epoch=198, best_val_loss=0.0061
  Inner fold 3: best_epoch=190, best_val_loss=0.0050
  Inner fold 4: best_epoch=146, best_val_loss=0.0042
  Inner fold 5: best_epoch=199, best_val_loss=0.0050
  Inner CV time: 107.7s
  Chosen number of epochs after inner CV: 190
Outer fold 5 metrics: test_mse=0.0049, test_rmse=0.0701, test_r2=0.8600, test_accuracy=0.8647
```

This design ensures that the model's performance reflects generalization to unseen genes rather than memorization of the scoring formula. If the model were merely relearning a fixed linear combination, the nested CV would not yield stable generalization metrics across independent folds. To maintain more clarity, we have explained the inner and outer loops more clearly in section **2.1**.

"In this framework, the inner loop was exclusively used to optimize model hyperparameters without exposing the outer test folds, whereas the outer loop evaluated performance on completely unseen data partitions to provide an unbiased estimate of generalization performance."

Therefore, the composite propensity score just serves as a supervision signal that encodes prior biological knowledge, and the GNN functions as a topology-aware refinement mechanism for disease gene prioritization. That's why we have called it a graph-based ranking and representation learning framework for target identification, rather than as a solo predictor of an externally measured biological outcome. The objective is ranking targets under integrative molecular criteria, where the composite

		score provides structured supervision and the GNN enhances it through network-level learning.<br><br>This model is supported by previous studies, where researchers trained on a large curated PPI dataset derived from the STRING database to identify closely associated genes using co-expression, experimentally determined interaction, database annotation, and an automated text-mining score calculated from the STRING database [1,2]. On the other hand, they also used to identify hub genes using Degree, Betweenness, and Closeness centralities, supported by recent studies [3–5]. Both are established methods for identifying key genes (**Supplementary File S1**). Instead of calculating them one by one, our model learns from both and ranks them.<br><br>Now the question is, are they accurate and relevant in a real data scenario? The top genes ranked by the model are core genes supported by independent network topologies, as well as independent network measures from the STRING database: TP53, CTNNB1, MYC, CDH1, GSK3B, ERBB2, CCND1, LRP5, LRP6, LEF1, SMAD3, EP300, GRB2, HNF4A, CDH17, WNT2, APC, TCF7L2, CDKN2A, and TOP2A.

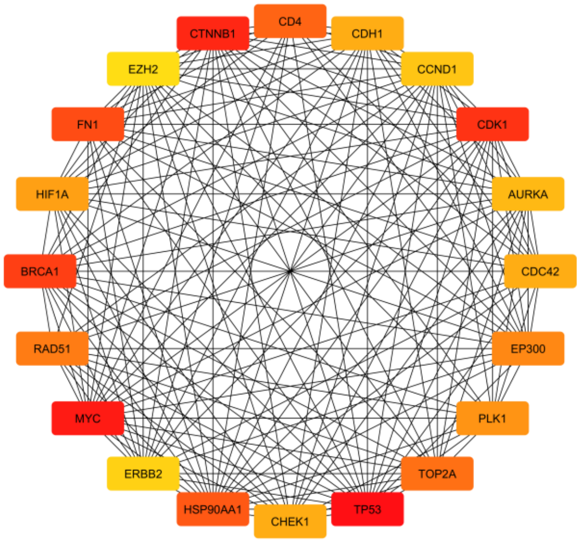

Figure 1. Hub gene identification from the degree centrality measure has no connection to our model. (Red = Top ranked, Orange = Middle ranked, Yellow = Low ranked).

Here, in **Figure 1**, we can see, even in a single method (degree centrality), several genes are overlapped with our model-predicted genes, which ensured the model's performance in an accurate direction.

Therefore, our model is not only supported by independent centralities, including degree, betweenness, and closeness centralities used by most of the previous studies during identification of hub genes, but also the integration of co-expression, experimentally determined interaction, database annotation, and an automated text-mining score calculated from the STRING database, making the model more advanced and comprehensive than any

		independent method used for the same purpose earlier.
**2.**	Consequently, the reported high AUC and R^2 values reflect internal consistency rather than genuine predictive performance, and they cannot be considered a valid validation metric.	The high AUC value does not mean the model is perfect or free from errors. If we see the details of the final fold train, validation, and test set confusion matrix, then we can understand the picture more clearly.<br><br>Train Set:<br><br>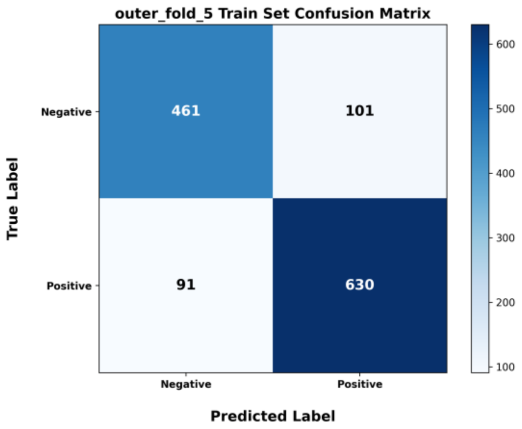<br><br><br>Validation Set:<br><br>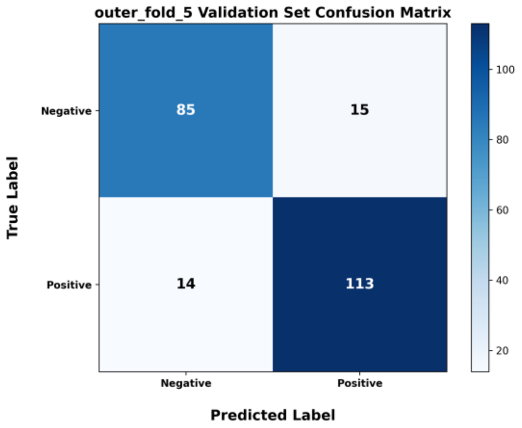<br><br><br>Test Set:

		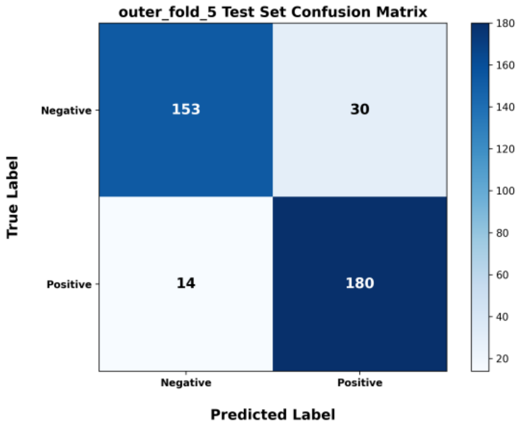 Figure 2a, b, c. Training, validation, and test sets confusion matrix.<br><br>These classification-based confusion matrix analyses on training, validation, and testing sets in **Figure 2a,b,** and **c** indicate the positive and negative distribution of genes, where the same label between true label and predicted label indicates accurate distribution (negative-negative and positive-positive), and a mislabel between them indicates inaccurate distribution (negative-positive, positive-negative).<br><br>To more clarify, the reported high AUC and R^2 values are not merely reflections of internal consistency but rather indicators of structured signal capture within a biologically integrated framework. The propensity score was constructed from heterogeneous features, including differential expression, network centrality (degree, betweenness, closeness), and GC pathway membership, thereby reducing the likelihood that performance arises from trivial self-reference.

		Moreover, regression and classification evaluations were conducted on training, validation, and independent test partitions to mitigate overfitting and to assess generalization within the defined feature space.<br><br>Therefore, we have interpreted it as validation of methodological coherence and ranking reliability rather than definitive proof of real-world prediction of clinical outcome. We have already shown how it performed in real world scenario in the explanation of **Figure 1** as an example in the previous response.<br><br>Hence, neither an individual classification analysis or regression analysis can accurately define the robust performance. This is the reason we used the model's integrated performance, including both classification and regression together, to make the model more accurate due to a ranking filtration of genes by regression and class distribution of genes by classification. Together, they ensured good performance of the model, though it is not free from errors (we can see it in the confusion matrix), which is a common attribute of a robust model.
	In addition, converting the continuous score into a binary classification using a threshold of 0.5 is statistically arbitrary and lacks biological or clinical justification.	We agree that arbitrary thresholding can introduce bias if not properly justified. In our framework, the 0.5 cutoff was not intended to imply a clinical decision boundary but rather represents the natural midpoint of a sigmoid-normalized score, corresponding to genes with above-average integrated pathogenic propensity within the modeled feature space. Thus, the threshold serves

as a pragmatic and interpretable separation between relatively high- and low-confidence candidates for prioritization purposes, not as a biologically definitive classification.

We have previously clarified that the regression was applied to rank the top 20 most influential genes. However, in the test set, which contains 377 genes with a threshold of 0.5 based on DEGs, network centralities, etc. So, the genes that ranked higher than a score of 0.5 were considered most important. In the manuscript, Table 2 shows that the predicted score of the last (20th) gene was 0.837. Therefore. Our goal was achieved adequately. We got our desired top 20 hub genes that could have most influenced GC pathogenesis within the 0.5 threshold.

Genes	Degree	Betweenness	Closeness	Present in Pathway	Importance Score	Predicted Score	Mutation Frequency
TP53	0.239	0.081	0.521	Yes	0.990	0.960	47.60%
CTNNB1	0.162	0.038	0.49	Yes	0.965	0.935	6.10%
MYC	0.19	0.04	0.5	Yes	0.948	0.931	1.40%
CDH1	0.102	0.012	0.461	Yes	0.899	0.889	8.20%
GSK3B	0.09	0.012	0.457	Yes	0.892	0.888	2.00%
ERBB2	0.1	0.012	0.458	Yes	0.921	0.888	8.80%
CCND1	0.1	0.006	0.454	Yes	0.904	0.879	0.70%
LRP5	0.023	0.001	0.383	Yes	0.947	0.878	1.40%
LRP6	0.021	0.001	0.378	Yes	0.870	0.877	2.00%
LEF1	0.049	0.004	0.414	Yes	0.867	0.869	0.70%
SMAD3	0.067	0.005	0.437	Yes	0.880	0.866	2.70%
EP300	0.107	0.01	0.46	No	0.861	0.865	2.70%
GRB2	0.08	0.011	0.443	Yes	0.853	0.865	0.70%
HNF4A	0.046	0.004	0.42	No	0.870	0.862	0.70%
CDH17	0.04	0.004	0.409	Yes	0.864	0.860	2.00%
WNT2	0.019	0.001	0.382	Yes	0.859	0.860	1.40%
APC	0.02	0	0.389	Yes	0.844	0.854	6.80%
TCF7L2	0.025	0.001	0.389	Yes	0.812	0.857	0.70%
CDKN2A	0.086	0.009	0.449	No	0.864	0.839	2.70%
TOP2A	0.111	0.005	0.448	No	0.834	0.837	1.50%

Whether our model is supported by real-world biological data or not, we have shown in **Figure 1**, where the most critical genes are ranked (Red = Top ranked, Orange = Middle ranked, Yellow = Low ranked). Hence, this is a model to predict prioritization strategy as an alternative to manual

		network analysis and different topological analyses independently, supported by real hub ranking (**Figure 1**).
3.	Although the addition of nested cross validation is a positive step regarding data leakage concerns, it still does not provide true external validation because all data sources originate from overlapping biological knowledge bases. Presenting cBioPortal confirmation as independent validation is methodologically incorrect since it belongs to the same information universe used during model construction. Therefore, the generalizability problem remains unresolved.	Sorry for the confusion. We did not consider cBioPortal confirmation as independent validation of our model. Rather, those are the variants that were validated from the cBioPortal database. To develop models, we have used mutation-prone genes but not used any specific SNPs or variants. The downstream analysis, in which we analyzed variants validated from the cBioPortal database. These 2 are completely different. 1st use of cBioPortal to train the models using genes, and 2nd use for validation of variants. We have re-evaluated the manuscript to identify any sentence that sounds like model validation from cBioPortal. We have now clearly defined that cBioPortal is for variant validation.<br><br>For the external validation scenario, we have already explained the phenomenon in the previous responses of how we have developed the composite score to supervise the model, and then distributed it under inner and outer loops for training, validation, and testing purposes.<br><br>The outer loop data are completely unseen samples with respect to model selection. It is the inner loop that is used for hyperparameter tuning and model selection, meaning the model indirectly sees the inner-loop validation folds during tuning. In contrast, the outer-loop test fold is held completely

		separate from any training or tuning process and is only used for final performance estimation (R^2, AUC, Accuracy, etc.). Therefore, performance reported from the outer loop reflects generalization to unseen data, whereas inner-loop results reflect optimization performance rather than unbiased predictive evaluation.<br><br>However, if you are indicating the 8th column of Table 2, where Mutation Frequency was reported, then we have to clarify that the frequency information was never exposed to the model; this is a completely external phenomenon that has not been fed and used to train the model. Hence, this frequency data definitely supports and validates our model's relevance to achieve our aim in this study.<br><br>However, if you are expecting that the model be derived exclusively from directly experimentally generated evidence independent of existing biological resources, then it is important to clarify that our framework is not designed to generate de novo experimental data, but rather to learn structured patterns from curated, experimentally derived knowledge embedded within transcriptomic datasets, protein–protein interaction networks, and pathway annotations. In other words, the model learns from aggregated empirical evidence encoded in these resources by integrating them into a unified predictive representation. Therefore, while it does not replace prospective wet-lab validation, it systematically leverages existing experimental

		knowledge to prioritize candidates in a data-driven manner, serving as a ranking tool whose outputs are intended to guide further experimental validation.<br><br>Therefore, we have provided the evidence of how much the model aligned with the real-world independent analysis that was performed by most of the previous studies to identify hub genes based on degree centrality (counting the total nodes that are connected with each gene or protein) (**Figure 1**). The output of our model is accurately aligned with the independent degree centrality.
4.	The application of FDR correction is an appropriate direction; however, the statement that significance was considered at $p<0.05$ across analyses is contradictory because, once FDR is applied, adjusted p values must be the primary inference criterion. Adding hazard ratios and confidence intervals to survival analysis is appropriate, yet without clinical covariates the results still do not reach the level of an interpretable prognostic model.	Thank you for you suggetion of covariate analysis. We never claim significance was considered at $p<0.05$ across analyses. We used FDR-adjusted p-values < 0.05 during the analysis of differentially expressed genes, and logrank p-value < 0.05 during survival analysis, and adjusted p-values <0.05 for cancer hallmarks analysis.<br><br>You can check the analysis from https://kmplot.com/ for Gastric cancer, where logrank p-value < 0.05 is applied.<br><br>The analysis for TP53:<br><br>https://kmplot.com/analysis/index.php?p=view&pa_id=27292044&show=bZJBbsQwCEXvknVVaRbd9DKIOGSC4hgLcGbaUe9eJ4vWycz28_15Bh6d4UqABs7j2H12I0aj7q2zSW6w0MCYqupa_kVOC95PYk9kN9TlnJBVsqizJEAHK7ryivHs6jHM9fUASiMppUCvSUDGNqMFyFEcIicCnzjMicyq43IoO90deonD64rx99b38v5xqIViLgssyKkOyeMT2-

		7KFakQZDHefrvRSVa-Tn4kpCulobX14jU-0vhnTGXpSbd5KdvcdJt4IAiUTJTTtcKE2c4wQaIoZIzk3pJuiwgThfmVBhjjDb_asGIEKypjHwnGGhlkqotsLUFUwTLVte9HctA32SQ94RWX_c723f38Ag<br><br>The analysis for APC:<br><br>https://kmplot.com/analysis/index.php?p=view&pa_id=27292045&show=bZJBbsQwCEXvknVVaRbd9DKIOGSC4hgLcGbaUe9eJ4vWycz28_15Bh6d4UqABs7j2H12I0aj7q2zSW6w0MCYqupa_kVOC95PYk9kN9TlnJBVsqizJEAHK7ryivHs6jHM9fUASiMppUCvSUDGNqMFyFEcIicCnzjMicyq43IoO90deonD64rx99b38v5xqIViLgssyKkOyeMT2-7KFakQZDHefrvRSVa-Tn4kpCulobX14jU-0vhnTGXpSbd5KdvcdJt4IAiUTJTTtcKE2c4wQaIoZIzk3pJuiwgThfmVBhjjDb_asGIEKypjHwnGGhlkqotsLUFUwTLVte9HctA32SQ94RWX_c723f38Ag<br><br>The analysis for CDH1:<br><br>https://kmplot.com/analysis/index.php?p=view&pa_id=27292047&show=bZJBbsQwCEXvknVVaRbd9DKIOGSC4hgLcGbaUe9eJ4vWycz28_15Bh6d4UqABs7j2H12I0aj7q2zSW6w0MCYqupa_kVOC95PYk9kN9TlnJBVsqizJEAHK7ryivHs6jHM9fUASiMppUCvSUDGNqMFyFEcIicCnzjMicyq43IoO90deonD64rx99b38v5xqIViLgssyKkOyeMT2-7KFakQZDHefrvRSVa-Tn4kpCulobX14jU-0vhnTGXpSbd5KdvcdJt4IAiUTJTTtcKE2c4wQaIoZIzk3pJuiwgThfmVBhjjDb_asGIEKypjHwnGGhlk

		qotsLUFUwTLVte9HctA32SQ94RWX_c723f38Ag<br><br>Due to https://kmplot.com/ not supporting clinical covariate analysis, we performed covariate analysis using TIMER 3.0 (https://compbio.cn/timer3/). The covariate analysis to interpret the different stages of cancer is presented in **Supplementary Table S11**. This is explained in the method section **2.14** and results section **3.14**.
5.	The justification of variant classification using a seven tool consensus cites literature support but does not resolve the independence assumption problem, and without measuring inter tool agreement bias persists. Therefore, inferential reliability remains limited.	We respectfully clarify that the seven-tool consensus strategy was not adopted merely as literature support only, but as part of a structured and widely accepted variant interpretation framework grounded in the American College of Medical Genetics and Genomics (ACMG) guidelines [6]. The ACMG framework explicitly recommends integrating multiple independent lines of computational and functional evidence rather than relying on a single predictive algorithm, precisely to mitigate tool-specific bias and improve robustness. The purpose of consensus-based classification is not to claim strict independence but to strengthen inferential confidence through concordance across heterogeneous methodologies (conservation-based, structure-based, and ensemble predictors). All of these tools are different from each other in terms of input, training, and methods. This is the reason most of the previous studies follow this strategy [7–11].

6.	Reframing structural analyses as hypothesis generating is scientifically appropriate, but since no quantitative thresholds linking energy change to functional loss are defined, the biological interpretability of the results remains weak. Similarly, presenting k means analysis as exploratory is acceptable, yet without confirmatory statistical validation its scientific contribution remains limited.	The goal of these analyses is not for statistical justification; it is to determine if there is any atomic divergence, including minor differences, between mutant and wild-type structures. It is a comparative study to understand if a mutation causes energy or atomic deviation from wild type structure.<br><br>Therefore, the primary objective of the structural analyses was not to establish quantitative predictive thresholds for functional loss, but rather to perform a comparative biophysical assessment to determine whether mutations induce measurable atomic-level divergence from the wild-type structure. Hence, we intentionally framed these findings as hypothesis-generating and comparative rather than definitive, which require experimental validation. Similarly, the k-means clustering was conducted as an exploratory tool to identify natural grouping patterns and structural variance trends within the dataset, not as a confirmatory, but rather as a comparative to denote any atomic divergence, even though it is minor. The scientific contribution of these analyses lies in providing mechanistic insight and comparative structural context that can guide and support subsequent experimental or quantitative validation, rather than serving as standalone statistical proof of functional consequence. This analysis is supportive and determined the variant divergence at the atomic and energetic level. To determine how they interfere at the function level, experimental studies are necessary.

		We have clearly stated this in the limitations of our discussion section:<br><br>"The basis of our study is hypothesis-driven with multi-layer computational analysis requiring further experimental studies for a definitive conclusion."
7.	Acknowledging circular reasoning as a limitation is positive, but because the dataset initially consists of cancer related genes, the model's ability to generate novel information has still not been demonstrated. Accepting that identifying TP53 is expected does not eliminate this methodological limitation.	Thank you for your comment on novel information generation. Our goal was not to generate novel information by discovering new genes; instead, prioritization of genes by ranking and classification that could have the most significant impact on GC development. Genes were prioritized if they have an interaction with other proteins within the whole network of proteins.<br><br>We agree that initiating the analysis from a cancer-enriched gene set constrains claims of de novo discovery. However, we have already clarified that the primary objective of the model was not to rediscover universally established drivers such as TP53, but to prioritize genes within a biologically relevant search space using an integrative, quantitative framework.<br><br>The model's contribution lies in its ability to rank genes based on multi-layered evidence: expression dysregulation, network centrality, and pathway context, thereby differentiating relative pathogenic propensity within the cancer-related pool. While we acknowledge that this design limits assertions of completely novel gene discovery beyond the initial dataset scope, it does not invalidate the model's

utility for refinement, prioritization, and hypothesis generation within a defined biological domain.

The calculated importance score based on network data and the model-predicted score are very close to each other, even though both are independently calculated from network data.

Genes	Degree	Betweenness	Closeness	Present in Pathway	Importance Score	Predicted Score	Mutation Frequency
TP53	0.239	0.081	0.521	Yes	0.990	0.960	47.60%
CTNNB1	0.162	0.038	0.49	Yes	0.965	0.935	6.10%
MYC	0.19	0.04	0.5	Yes	0.948	0.931	1.40%
CDH1	0.102	0.012	0.461	Yes	0.899	0.889	8.20%
GSK3B	0.09	0.012	0.457	Yes	0.892	0.888	2.00%
ERBB2	0.1	0.012	0.458	Yes	0.921	0.888	8.80%
CCND1	0.1	0.006	0.454	Yes	0.904	0.879	0.70%
LRP5	0.023	0.001	0.383	Yes	0.947	0.878	1.40%
LRP6	0.021	0.001	0.378	Yes	0.870	0.877	2.00%
LEF1	0.049	0.004	0.414	Yes	0.867	0.869	0.70%
SMAD3	0.067	0.005	0.437	Yes	0.880	0.866	2.70%
EP300	0.107	0.01	0.46	No	0.861	0.865	2.70%
GRB2	0.08	0.011	0.443	Yes	0.853	0.865	0.70%
HNF4A	0.046	0.004	0.42	No	0.870	0.862	0.70%
CDH17	0.04	0.004	0.409	Yes	0.864	0.860	2.00%
WNT2	0.019	0.001	0.382	Yes	0.859	0.860	1.40%
APC	0.02	0	0.389	Yes	0.844	0.854	6.80%
TCF7L2	0.025	0.001	0.389	Yes	0.812	0.857	0.70%
CDKN2A	0.086	0.009	0.449	No	0.864	0.839	2.70%
TOP2A	0.111	0.005	0.448	No	0.834	0.837	1.50%

Moreover, if we exclude, for example, differentially expressed top genes including TP53, the model can still capture them due to not because it learned from the input but because of the high network centrality of TP53: degree centrality which counts a node's direct connections, closeness centrality, which shows how close it is to all other nodes, betweenness centrality, which indicates how often it acts as a bridge between nodes and other important features that influenced the network topology.

8.

In conclusion, the revisions improved clarity and transparency but did not resolve the fundamental statistical problems. The model does not predict a true clinical or biological outcome, no independent

We previously clarified that the objective of the model was not to predict a direct clinical outcome, but to develop an integrative gene-prioritization framework. Accordingly, the performance metrics are intended to assess internal generalization and ranking stability within this defined modeling

	validation exists, performance metrics remain misleading due to the derived target variable, and most interpretations remain exploratory. Therefore, the revisions are partially corrective but still insufficient methodologically, and the study can only be considered reliable after strong external validation, a true dependent variable definition, and stricter limitation of causal interpretations.	space, rather than to imply direct clinical predictive utility. We fully acknowledge that the target variable is derived from integrated biological features and therefore we have consistently framed the findings as exploratory and prioritization-oriented rather than causal or diagnostic. The implementation of strict nested cross-validation was designed to mitigate overfitting and circularity within the available dataset, but we agree that a complete external validation where genes have no relation with the input genes has not been utilized for this model, because to perform prioritization of genes by any model, the model has to be a boundary of features. In the case of the discovery of new gene information, a complete external validation would substantially strengthen the inferential robustness.<br><br>In our case, the test set (20%) is independent, though it is definitely based on the initial features. This is the reason we have provided frequency information in **Table 2** of the manuscript as completely independent information to support and validate our findings. This information was even though retrieved from the cBioPortal database, but the model was never trained with the frequency data. The top 20 genes exhibited significant mutation frequency, denoting the model ranking system in the right direction. We have also performed a degree analysis, which also supports our findings in a real application scenario (**Figure**

		**1**).<br><br>Hope this response clarified your independent validation query and data learning system of the model:<br><br>1. Composite score for supervising the model.<br>2. Inner loops for training the model using known data.<br>3. Outer loop for performance evaluation and external validation using 20% of the unseen data.<br>4. Real-world independent validation of the model using a manual degree centrality analysis that calculates connecting nodes and identifies hubs (**Figure 1**).<br><br>However, if you think a complete external validation is still necessary, then we will definitely consider your suggestion.<br><br>We ensured the objective of our model and the real-world performance of the model. If you have any further queries, please let us know. We remain to explain and justify any further queries.<br><br>Finally, we want to clarify that we have followed the previous studies to design our study. The graph-neural network model for gene prioritization study is supported by several recent studies [12–14], and the methodology used in this study is also supported by recent studies [15–17].

We sincerely appreciate your insightful feedback and recommendations. Your perspective helped us clarify our methodology and the model's core contributions, which significantly strengthened the overall quality of the manuscript. We appreciate your time and expertise to meaningfully enhance our manuscript.

Honorable Reviewer 2:

I thank the authors for the detailed revision and point-by-point response. The manuscript has clearly improved in structure, clarity, and reporting transparency. Several concerns raised in the initial review have been acknowledged and textually addressed. However, in many instances the revision resolves presentation issues rather than the underlying methodological limitations. As a result, the interpretability of the biological conclusions remains only partially improved.

Major Comments

SL.	Comments/Questions/Instructions/Guidelines	Responses of the Authors
1.	Regarding conceptual novelty, the authors now explicitly state that the aim of the study is prioritization rather than discovery. While this clarification is helpful, it effectively confirms the original concern that the analysis primarily re-identifies well-established gastric cancer drivers such as TP53, CDH1, and APC rather than generating new biological insight. Thus, although the authors have answered the question about novelty, the revision does not fundamentally strengthen the scientific contribution beyond integrative ranking.	We sincerely thank you for the precise evaluation of our revised manuscript. The multi-layer analysis of our study ranked GC-associated genes, prioritized key genes, screened key variants, analyzed their gene expression changes and gene regulators, and compared them with wild-type tumor suppressor proteins, providing an integrated set of findings. Although they are derived from prioritization, the integrated findings of our study provide a novel framework for future experimental studies and decision-making, as previous studies have focused only on single proteins and their GC variants. Therefore, together our findings provide 2 novel

		insights into how the GC development could happen due to pathogenic variants, and how abnormal expression dysregulates key proteins of the GC pathway. The findings also provide an overview of the potential mechanism of GC development due to the dysregulation of key tumor suppressor proteins of the GC pathway (**Figure 16**).<br><br>Hence, our multilayer study definitely provides novel insights by not generating novel gene information but providing an integrated mechanistic framework that could help in decision-making for further studies.<br><br>Regarding conceptual novelty, we acknowledge that the primary objective of this study is prioritization rather than de novo discovery. Our intention is to provide a transparent and reproducible integrative framework that systematically ranks candidate genes in gastric cancer, including both established drivers (e.g., TP53, CDH1, APC) and less-characterized candidates. We have further prioritized these 3 drivers: TP53, CDH1, and APC, not by chance but due to 3 important considerations.<br><br>1. They appeared in the top 20 most influential genes ranked by our model.<br>2. The mutation frequencies are higher than those of most of the other top-ranked genes, which externally validates our findings (8th column of **Table 2**).

		3. They are known as tumor suppressor proteins in the GC pathway.<br><br>Additionally, we agree that the work should be interpreted as a computational prioritization resource, and we have revised the text to ensure that claims are aligned with this scope in the discussion and conclusion.
2.	For the graph neural network framework, the authors now define the model output as a composite "propensity score" derived from expression dysregulation, network centrality, and pathway membership. This clarification improves transparency but simultaneously reveals that the model is trained to predict a constructed heuristic score based on the same input features. Consequently, the reported performance metrics represent successful reconstruction of an engineered ranking rather than predictive biological inference. The authors clarified whether the task is supervised or semi-supervised and added baseline comparisons, which addresses part of the methodological description. However, the concern about inflated performance due to feature selection and lack of biological validation remains unresolved.	Concerning the graph neural network framework, the model is indeed trained to learn a composite propensity score derived from expression dysregulation, network topology, and pathway membership. Our objective was not to claim independent biological prediction but to evaluate whether a GNN can effectively integrate heterogeneous features into a stable and generalizable ranking.<br><br>While the propensity score is indeed constructed from biologically meaningful components (expression dysregulation, network topology, and pathway context), and the framework is indeed supervised in structure, the model does not simply reconstruct a fixed linear heuristic; rather, it learns non-linear relationships and higher-order interactions across graph-structured data that cannot be captured by direct feature aggregation alone. If the task were merely a deterministic reconstruction of an engineered score, complex graph propagation would not yield stable generalization across strictly

separated nested folds.

The model is evaluated using a fully implemented nested cross-validation strategy (5×5 folds), where hyperparameter selection (optimal epoch number) is performed exclusively within the inner loop, and generalization performance is estimated only on held-out outer test folds. Importantly, the test folds are never exposed during model selection or training, and performance metrics (MSE, RMSE, R^2, accuracy, AUC-ROC, AUC-PR) are computed strictly on unseen outer-fold data. This design prevents information leakage and mitigates overfitting due to feature selection or epoch tuning.

The last fold (5th) during model development:

```
  Inner fold 1: best_epoch=195, best_val_loss=0.0052
  Inner fold 2: best_epoch=198, best_val_loss=0.0061
  Inner fold 3: best_epoch=190, best_val_loss=0.0050
  Inner fold 4: best_epoch=146, best_val_loss=0.0042
  Inner fold 5: best_epoch=199, best_val_loss=0.0050
  Inner CV time: 107.7s
  Chosen number of epochs after inner CV: 190
Outer fold 5 metrics: test_mse=0.0049, test_rmse=0.0701, test_r2=0.8600, test_accuracy=0.8647
```

Moreover, the purpose of the composite score is to operationalize multidimensional biological relevance into a measurable target for ranking, not to claim direct prediction of an independent clinical outcome.

We acknowledge that the target variable is derived from biologically informed features, and therefore, the performance metrics reflect internal consistency and ranking stability within this integrative framework rather than external

		biological validation. However, the point to be noted is that the strict node-level separation, nested cross-validation, and baseline comparisons were implemented specifically to mitigate feature re-learning and inflated performance.<br><br>Accordingly, we have clarified and positioned the framework as a rigorously evaluated integrative ranking model rather than a de novo biological predictor.
3.	The issue of circular reasoning has been acknowledged by the authors, who now discuss the expected identification of TP53 as a hub and provide an option to exclude known pathway genes. This represents a partial improvement in transparency, but the study still does not demonstrate discovery capability independent of prior biological knowledge. Therefore, the core concern is acknowledged but not experimentally resolved.	The identification of canonical genes such as TP53 was not presented as evidence of novel discovery, but rather demonstrated that the integrative framework captures biologically meaningful signals consistent with established knowledge. The option to exclude known pathway genes was introduced precisely to test the model's dependence on predefined annotations and to evaluate whether network topology and expression-driven features alone can still generate structured prioritization.<br><br>While this sensitivity analysis improves methodological rigor and maintains gene prioritization, we acknowledge that it does not have a full discovery capability independent of prior biological knowledge, particularly because the underlying interaction networks and pathway resources are themselves derived from accumulated experimental evidence.

		Therefore, we do not claim de novo biological discovery beyond the defined knowledge space; instead, we position the framework as a systematic prioritization and signal-integration tool whose novelty lies in quantitative ranking within a constrained domain.<br><br>This prioritization guides further downstream analysis using the most influential genes for GC pathogenesis.
4.	The authors appropriately revised the structural and energetic analyses by softening causal language and describing them as hypothesis-generating rather than definitive. Similarly, the atomic-level clustering analysis has been reframed as exploratory. These changes satisfactorily address the overinterpretation concern from a reporting standpoint, although the analyses remain descriptive in nature.	Regarding the structural, energetic, and atomic-level clustering analyses, we agree that these components are descriptive and do not establish causality, and we have ensured that the manuscript reflects this more cautious interpretation.<br><br>Our intention was not to claim definitive functional impairment based solely on computational structural deviation, but to provide a comparative biophysical context illustrating whether mutations induce measurable atomic-level divergence from the wild-type conformation. Similarly, the clustering analysis was designed to detect structural patterning and grouping tendencies rather than to serve as a formal inferential test. While these analyses are descriptive by nature, we believe they contribute meaningful hypothesis-driven mechanistic insight by highlighting consistent structural trends that can guide subsequent experimental validation. We have therefore

		deliberately limited interpretative scope and causal language to ensure that conclusions remain proportionate to the methodological strength of the evidence presented. For any definitive claim, we believe, experimental validation is necessary.
5.	The survival analyses have been improved by including false discovery rate correction, hazard ratios, and explicit acknowledgment that associations are correlative. Nevertheless, the analyses still lack adjustment for clinical covariates such as stage or treatment, meaning the biological interpretation remains limited. Thus, the statistical reporting is improved but the inferential strength remains modest.	For the survival analyses, we have incorporated false discovery rate correction, reported hazard ratios with confidence intervals, and explicitly stated the correlative nature of these associations. We acknowledge that multivariable adjustment for the clinical covariate, stage of cancer, would strengthen the analysis.<br><br>Therefore, we performed covariate analysis using TIMER 3.0 (https://compbio.cn/timer3/). The covariate analysis to interpret the different stages of cancer is presented in **Supplementary Table S11**. This is explained in the method section **2.14** and results section **3.14**.
6.	The manuscript length and redundancy have been reduced, and several secondary analyses have been moved to supplementary material, which adequately addresses the scope concern. Minor issues such as overly assertive language, terminology consistency, and code availability have also been corrected, and the inclusion of a public repository improves reproducibility. The technical questions were answered descriptively, though largely without	We sincerely thank you for acknowledging the improvements in manuscript structure, clarity, and reproducibility. Regarding the technical questions, we provided detailed methodological clarifications to improve transparency.<br><br>We acknowledge that further quantitative benchmarking and independent validation could strengthen the empirical depth of the study, and we present the current work as a rigorously documented and internally validated framework

	additional quantitative validation.	whose conclusions are appropriately bounded by the available evidence.<br><br>To justify our external validation or independent validation, we have provided external evidence to support our model.<br><br>We have provided mutation frequency information of genes in column 8 of **Table 2** of the manuscript as completely independent information to support and validate our findings. This information was, though, retrieved from the cBioPortal database, but the model was never trained with the frequency data. The top 20 genes exhibited significant mutation frequency, denoting the model ranking system in the right direction.<br><br>We have also performed a degree analysis, which also supports our findings in a real application scenario (**Figure 1**).<br><br>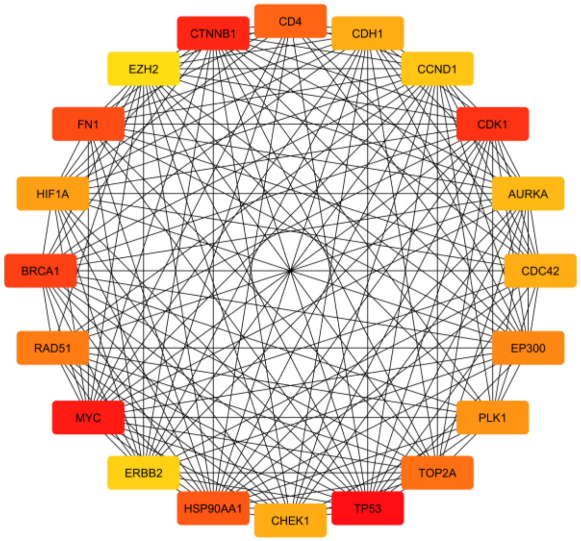

		**Figure 1.** Hub gene identification from the degree centrality measure has no connection to our model. (Red = Top ranked, Orange = Middle ranked, Yellow = Low ranked).<br><br>The top genes ranked by the model measures from the STRING database: TP53, CTNNB1, MYC, CDH1, GSK3B, ERBB2, CCND1, LRP5, LRP6, LEF1, SMAD3, EP300, GRB2, HNF4A, CDH17, WNT2, APC, TCF7L2, CDKN2A, and TOP2A.<br><br>Here, in **Figure 1**, we can see, even in a single method (degree centrality), several genes are overlapped with our model-predicted genes, which ensured the model's performance in an accurate direction.<br><br>Therefore, our model is not only supported by independent centralities, including degree, betweenness, and closeness centralities used by most of the previous studies during identification of hub genes, but also the integration of co-expression, experimentally determined interaction, database annotation, and an automated text-mining score calculated from the STRING database, making the model more advanced and comprehensive than any independent method used for the same purpose earlier.<br><br>Additionally, in the case of survival analysis, we

		have provided covariate analysis to interpret the different stages of cancer, to validate survival analysis as presented in **Supplementary Table S11**.
7.	Overall, the revision substantially improves clarity, organization, and transparency. However, the central methodological concerns — particularly the non-independent training objective, limited biological validation, and reliance on rediscovery of known drivers — remain only partially resolved. The manuscript now reads as a computational prioritization resource rather than a predictive or mechanistic framework, and the conclusions should be interpreted accordingly.	We appreciate your recognition of improvements in clarity, organization, and reproducibility.<br><br>Firstly, we have explained the use of a biologically constructed training objective, how we split and feed it into the model for training, and how we separate the inner loops (where data were used to train the model) from the outer loops (where unseen data were used to evaluate the model's performance during model development).<br><br>Example of the last fold (5th) during model development:<br><br>`Inner fold 1: best_epoch=195, best_val_loss=0.0052`<br>`Inner fold 2: best_epoch=198, best_val_loss=0.0061`<br>`Inner fold 3: best_epoch=190, best_val_loss=0.0050`<br>`Inner fold 4: best_epoch=146, best_val_loss=0.0042`<br>`Inner fold 5: best_epoch=199, best_val_loss=0.0050`<br>`Inner CV time: 107.7s`<br>`Chosen number of epochs after inner CV: 190`<br>`Outer fold 5 metrics: test_mse=0.0049, test_rmse=0.0701, test_r2=0.8600, test_accuracy=0.8647`<br><br>Secondly, we have justified the absence of independent external biological validation in the manuscript's mutation frequency analysis (Table 2) and in the independent degree centrality analysis (**Figure 1**).<br><br>Finally, we have explained that the model's ability to capture established driver genes remains, even though we remove them as

		pathway genes, for example, TP53, due to learning from network topologies. The TP53 node is connected to a large number of nodes within the network; hence, the model's prioritizing TP53 as a key driver is obvious.<br><br>Overall, our intention was not to present the framework as a definitive predictor of biological outcome, but rather as an integrative computational prioritization resource that systematically aggregates multi-layered biological evidence into a structured ranking strategy. This multi-layer integration makes our study a proposed mechanistic framework for GC that consists of molecular components that are highly significant and relevant to GC, unlike the discovery of components that may have a significant impact on GC or may not. Therefore, we believe this integrated framework will definitely guide further experimental studies regarding GC pathogenesis.<br><br>We have ensured the conclusion is derived from internally validated, hypothesis-generating, and supportive of downstream studies rather than as a standalone proof of novel biological causality or clinical predictiveness.<br><br>However, if you have any further queries, please let us know. We remain to explain and justify any further queries.<br><br>At the end, we want to clarify that we have

		followed the previous studies to design our study. The graph-neural network model for gene prioritization study is supported by several recent studies [12–14], and the methodology used in this study is also supported by recent studies [15–17].
Thank you for the thoughtful feedback. Your perspective was exactly what we needed to explain and justify the methodology and make our findings stand out more clearly. We are grateful for the time you put into helping us improve our manuscript.		

References

1. Ullanat V, Jing B, Sledzieski S, Berger B. Learning the language of protein-protein interactions. Nature Communications 2026 17:1. 2026;17: 1199-. doi:10.1038/s41467-025-67971-3

2. Hu L, Wan L, Han F, Huang Y, Zhu G, Cao W, et al. Optimizing anti-seizure medication selection for epilepsy in EAST/SeSAME syndrome: Insights from DrugBank and STRING databases. Genes Dis. 2025;106: 101852. doi:10.1016/j.gendis.2025.101852

3. Chevalier G, Udovin L, Otero-Losada M, Bordet S, Perez-Lloret S, Capani F. MicroRNAs and rs1803274 SNP-based BuChe downregulation are associated with metabolic syndrome through ghrelin hydrolysis and expression quantitative trait loci regulation in PD patients. Front Mol Neurosci. 2025;18: 1635201. doi:10.3389/fnmol.2025.1635201

4. Yu M, Tan Q, Dong W, Xiang B. Identification and prioritization of gene sets associated with schizophrenia risk by network analysis. Psychopharmacology 2025. 2025; 1–11. doi:10.1007/s00213-025-06867-y

5. Akgüller Ö, Balcı MA, Cioca G. A Multi-Modal Graph Neural Network Framework for Parkinson's Disease Therapeutic Discovery. International Journal of Molecular Sciences 2025, Vol 26, Page 4453. 2025;26: 4453. doi:10.3390/ijms26094453

6. Masson E, Zou W Bin, Génin E, Cooper DN, Le Gac G, Fichou Y, et al. Expanding ACMG variant classification guidelines into a general framework. Human Genomics 2022 16:1. 2022;16: 31-. doi:10.1186/s40246-022-00407-x

7. Doffe F, Carbonnier V, Tissier M, Leroy B, Martins I, Mattsson JSM, et al. Identification and functional characterization of new missense SNPs in the coding region of the TP53 gene. Cell Death & Differentiation 2020 28:5. 2020;28: 1477–1492. doi:10.1038/s41418-020-00672-0

8. Ali R, Sultan A, Ishrat R, Saini D, Hayat S, Khan NJ. Structural and functional consequences of non-synonymous SNPs within the LAMA2 protein: a molecular dynamics perspective. J Biomol Struct Dyn. 2024 [cited 4 Mar 2025]. doi:10.1080/07391102.2024.2426756

9. Ahmed A, Islam KMT, Sarker S, Mahmud S. In-silico analysis of structural and functional impact of missense SNPs of human CTSD protein on cancers and identification of potential novel cancer SNP biomarkers. Network Modeling Analysis in Health Informatics and Bioinformatics 2025 14:1. 2025;14: 152-. doi:10.1007/S13721-025-00644-9

10. Hossain S, Bin Manjur OH, Shimu MSS, Sultana T, Naim MR, Siddique S, et al. In silico evaluation of missense SNPs in cancer-associated Cystatin A protein and their potential to disrupt Cathepsin B interaction. Heliyon. 2025;11: e42478. doi:10.1016/j.heliyon.2025.e42478

11. Ahamed MS, Khanam R, Islam KMT, Tabassum F, Amin M Al, Fardous J, et al. Bioinformatics-driven identification of pathogenic missense nsSNPs in the human proto-oncogene SRC and cancer susceptibility. Journal of Genetic Engineering and Biotechnology. 2025;23: 100618. doi:10.1016/J.JGEB.2025.100618

12. Islam KMT, Shibly AZ. Network and pharmacophore guided and BCL2 and HSP90AA1 targeted drug repurposable approaches against rheumatoid arthritis mediated diffuse large B-cell lymphoma. Int J Biol Macromol. 2025;325: 146985. doi:10.1016/J.IJBIOMAC.2025.146985

13. Hossain MdM, Adhikari J, Shibly AZ. Deep learning, network pharmacology and computational simulation reveal multi-target therapeutic mechanisms of Vitis vinifera (Grapes) compounds against Alzheimer's disease. In Silico Research in Biomedicine. 2025;1: 100095. doi:10.1016/j.insi.2025.100095

14. Islam KMT, Mahmud S. SIRT7 as a context-dependent biomarker and therapeutic target: Insights from a pan-cancer study. Ahmed F, editor. PLoS One. 2026;21: e0342269. doi:10.1371/journal.pone.0342269

15. Alipeddi RR, Rani DN, Sampanmudumby P, Hanumanth SR. Computational analysis of functional, structural and pathogenic impacts of missense SNPs in the human SOCS3 gene. Mutation Research - Fundamental and Molecular Mechanisms of Mutagenesis. 2026;832: 111925. doi:10.1016/j.mrfmmm.2025.111925

16. Sivakumar K, Sidhic N, Subbiah U, Mookkandi S. Characterization of Pathogenic Missense Mutations in Nuclear Encoded Mitochondrial Dynamin -1 like protein. Mutation Research - Fundamental and Molecular Mechanisms of Mutagenesis. 2026; 111928. doi:10.1016/j.mrfmmm.2026.111928

17. Shadhin MST, Mia MS, Das Topu TK, Tanmoy MSI, Aktar S, Sultana J, et al. In silico functional, structural, and pathogenicity assessment of single nucleotide polymorphisms in

the human SOX9 gene. Scientific Reports 2025 16:1. 2025;16: 941-. doi:10.1038/s41598-025-30462-y
