# Supplementary material for: Computational screening of oncogenic genetic variations in tumor suppressor proteins driving gastric cancer pathogenesis
Source: PLoS One. 2026 Sep 17;21(9):e0358440. doi: 10.1371/journal.pone.0358440 (PMC13585236; doi:10.1371/journal.pone.0358440)
Supplement: Supplementary file 4 [file pone.0358440.s002.docx]

**Supplementary Figure S2.** Titration curve analysis to enable direct comparison of ionization behaviors. Comparison of TP53, CDH1, and APC wild-type and mutant types **(A)**, **(B)**, and **(C)**, respectively.
