# Supplementary material for: Computational screening of oncogenic genetic variations in tumor suppressor proteins driving gastric cancer pathogenesis
Source: PLoS One. 2026 Sep 17;21(9):e0358440. doi: 10.1371/journal.pone.0358440 (PMC13585236; doi:10.1371/journal.pone.0358440)
Supplement: Supplementary file 5 [file pone.0358440.s010.docx]

**Supplementary Table S6:** Estimated Protein Energies before minimization and after minimization.

| Proteins | SNP (Allele) | Energy Calculation (KJ/mol) | |
| --- | --- | --- | --- |
|  |  | Before Minimization | After Energy Minimization |
| APC  (Tunor Suppressor Domain) | Wild Type | -6637.889 | -8145.293 |
|  | R141S | -6314.409 | -7861.43 |
| CDH1  (Tunor Suppressor Domain) | Wild Type | -2184.448 | -3973.105 |
|  | G239R | -2488.543 | -4294.239 |
|  | D254Y | -2245.629 | -4033.903 |
| TP53  (Tunor Suppressor Domain) | Wild Type | -31026.859 | -46581.02 |
|  | D281A | -31050.859 | -46555.781 |
|  | D281G | -30961.692 | -46467.391 |
|  | D281H | -31065.76 | -46609.34 |
|  | R273P | -29956.252 | -45959.559 |
|  | R273G | -30742.311 | -46223.998 |
|  | G266E | 36920304 | -44596.148 |
|  | G266R | 401914.126 | -45043.535 |
|  | L265P | -26722.627 | -46173.676 |
|  | I254N | -31201.73 | -46756.992 |
|  | T253P | -28151.945 | -46074.422 |
|  | R248P | 929769.25 | -45124.496 |
|  | M246K | -29891.287 | -46322.273 |
|  | G245D | -5257.152 | -45826.109 |
|  | G245V | 32427.725 | -45336.906 |
|  | G245S | -30941.131 | -46609.344 |
|  | G245C | 84806.836 | -45452.832 |
|  | G244A | -30855.445 | -46648.668 |
|  | G244V | -2495.494 | -45693 |
|  | G244D | 358253.531 | -45768.383 |
|  | G244R | 139626.016 | -45758.652 |
|  | G244S | -30453.701 | -46342.996 |
|  | C242W | 93207960 | -44167.48 |
|  | C242Y | 7652196.5 | -44643.031 |
|  | S240R | 132832624 | -42449.18 |
|  | S240G | -30906.293 | -46459.371 |
|  | C238W | 77225128 | -39255.828 |
|  | C238F | 246466784 | -44578.555 |
|  | C238Y | 7698648268 | -42004.207 |
|  | C238G | -30922.057 | -46468.863 |
|  | C238R | 303159.406 | -45038.719 |
|  | Y236H | -27127.955 | -46316.484 |
|  | Y234H | -27085.008 | -46071.84 |
|  | Y234D | -27107.793 | -46058.59 |
|  | Y220C | -26865.359 | -45879.449 |
|  | Y220S | -26919.23 | -45907.207 |
|  | Y220H | -26920.797 | -45924.336 |
|  | V218E | -26011.471 | -46135.617 |
|  | R213P | 14597.831 | -45252.074 |
|  | G199V | 451260864 | -44955.715 |
|  | R196P | 451262176 | -44482.199 |
|  | H193N | 451288800 | -44663.547 |
|  | C176R | 466536800 | -43618.758 |
|  | C176F | 451770240 | -43276.691 |
|  | C176Y | 457240640 | -42236.082 |
|  | R175L | 451309120 | -43903.688 |
|  | R175G | 451308960 | -43839.66 |
|  | A159D | 562707840 | -40935.879 |
|  | P151R | 562537728 | -41034.176 |
|  | A138P | 562325248 | -41148.633 |
|  | C135R | 664825728 | -41204.938 |
|  | K132N | 562322432 | -41573.605 |
|  | K132R | 562328512 | -41524.742 |
|  | T125R | 562496256 | -40589.766 |
|  | T125K | 562330176 | -40885.309 |
