# Supplementary material for: Computational screening of oncogenic genetic variations in tumor suppressor proteins driving gastric cancer pathogenesis
Source: PLoS One. 2026 Sep 17;21(9):e0358440. doi: 10.1371/journal.pone.0358440 (PMC13585236; doi:10.1371/journal.pone.0358440)
Supplement: Supplementary file 6 [file pone.0358440.s015.docx]

**Supplementary Table S11.** Covariate analysis of survival in different stages of cancer.

| **Gene Model** | **Cancer** | **Variable** | **Hazard Ratio** | **95% CI** | **p-value** | **Significance Level** |
| --- | --- | --- | --- | --- | --- | --- |
| **TP53 model** | COAD | Stage I | 0.179 | 0.055 – 0.587 | 0.004 | ** |
|  |  | Stage IIA | 0.269 | 0.133 – 0.545 | <0.001 | *** |
|  |  | Stage IIIB | 0.292 | 0.114 – 0.748 | 0.010 | * |
|  |  | Stage IV | 3.634 | 2.022 – 6.530 | <0.001 | *** |
| **APC model** | STAD | Stage IA | 0.132 | 0.032 – 0.547 | 0.005 | ** |
|  |  | Stage IB | 0.317 | 0.148 – 0.676 | 0.003 | ** |
|  |  | Stage II | 0.278 | 0.118 – 0.654 | 0.003 | ** |
|  |  | Stage IIA | 0.232 | 0.092 – 0.585 | 0.002 | ** |
|  |  | Stage IIB | 0.368 | 0.199 – 0.682 | 0.001 | ** |
|  |  | Stage IIIA | 0.534 | 0.318 – 0.897 | 0.018 | * |
|  |  | Stage IIIB | 0.527 | 0.313 – 0.885 | 0.015 | * |
| **CDH1 model** | STAD | Stage IA | 0.138 | 0.033 – 0.570 | 0.006 | ** |
|  |  | Stage IB | 0.313 | 0.147 – 0.667 | 0.003 | ** |
|  |  | Stage II | 0.273 | 0.116 – 0.642 | 0.003 | ** |
|  |  | Stage IIA | 0.232 | 0.092 – 0.583 | 0.002 | ** |
|  |  | Stage IIB | 0.367 | 0.198 – 0.679 | 0.001 | ** |
|  |  | Stage IIIA | 0.513 | 0.304 – 0.864 | 0.012 | * |
|  |  | Stage IIIB | 0.516 | 0.307 – 0.867 | 0.012 | * |

*=Significant, **=Highly Significant, ***=Extremely Significant
