# Supplementary material for: Computational screening of oncogenic genetic variations in tumor suppressor proteins driving gastric cancer pathogenesis
Source: PLoS One. 2026 Sep 17;21(9):e0358440. doi: 10.1371/journal.pone.0358440 (PMC13585236; doi:10.1371/journal.pone.0358440)
Supplement: Supplementary file 8 [file pone.0358440.s020.docx]

**Responses to the honorable reviewers’ s comments**

**Honorable Reviewer 1:**

The authors have substantially improved the manuscript compared with the previous version. The clarification of the graph neural network framework, inclusion of nested cross-validation procedures, additional details regarding data partitioning strategies to minimize information leakage, and improved reporting of model performance metrics have strengthened the methodological transparency of the study. The computational workflow is now considerably clearer and the overall presentation has improved substantially.

Nevertheless, several minor issues remain that should be addressed before publication.

| **SL.** | **Comments/Questions/Instruction/Guidelines** | **Responses of the Authors** |
| --- | --- | --- |
| **1.** | First, although the authors have clarified the construction of the composite propensity score used as the target variable, a brief discussion acknowledging the potential circularity associated with predicting a score derived from network and expression features would improve transparency. | We acknowledge this limitation and have added the following statement in the limitations section: "Though the GCN prioritization framework minimizes circular inference through non-linear network propagation, the composite propensity score is partly derived from input features, which may introduce potential circularity that necessitates cautious interpretation." |
| **2.** | Second, the rationale for selecting the 0.5 threshold used for binarization of propensity scores should be explained more explicitly, even if only as a practical prioritization criterion. | To improve transparency, we have added the following statement in the limitations section: "The 0.5 sigmoid-normalized threshold for binarizing propensity scores was adopted as a practical prioritization boundary rather than a biologically optimized cutoff, hence may not optimally discriminate pathogenic candidates across all biological contexts." |
| **3.** | Third, while the use of multiple pathogenicity prediction tools increases confidence in variant prioritization, the limitations arising from potential dependencies among these tools should be acknowledged in the discussion section. | As suggested, the following limitation has been included in the discussion section: "The use of multiple pathogenicity prediction tools enhances variant prioritization confidence; however, potential algorithmic dependencies and shared training databases among these tools may introduce correlated predictions, which could overestimate the robustness of variant classification." |
| 4. | Finally, the survival analyses would benefit from reporting effect size estimates whenever available and from a brief discussion regarding the limitations of unadjusted survival analyses based on publicly available datasets. | Following your recommendation, the limitations section now includes: "Survival analyses were conducted without covariate adjustment and effect size reporting due to the constraints of publicly available datasets." |
| We sincerely appreciate your constructive suggestions to acknowledge the minor limitations in the discussion section of our manuscript for more scientific clarity. | | |
