# Supplementary material for: Computational screening of oncogenic genetic variations in tumor suppressor proteins driving gastric cancer pathogenesis
Source: PLoS One. 2026 Sep 17;21(9):e0358440. doi: 10.1371/journal.pone.0358440 (PMC13585236; doi:10.1371/journal.pone.0358440)
Supplement: Supplementary file 9 [file pone.0358440.s019.docx]

06 April, 2026

Amr Ahmed El-Arabey
Academic Editor
PLOS ONE

Dear Professor,

Special gratitude to you for allowing us to resubmit a more revised and updated version of our manuscript entitled **"Computational screening of oncogenic genetic variations in tumor suppressor proteins driving gastric cancer pathogenesis" (Manuscript ID: PONE-D-25-52287R2)** to the PLOS ONE journal as an original research article. We have revised the manuscript based on the reviewers’ comments and journal requirements. In response to valuable suggestions, we have modified, revised, explained, and justified the manuscripts. Additionally, we have provide more clear explanation regarding model validation and performance, stability, atomic and energetic analysis, and survival and covariate analysis. We hope our response below will successfully address the reviewer’s specific points and improve the manuscript. Please let us know if you have any additional questions or comments. Finally, we would like to express our thanks and gratitude for your kind cooperation.

Sincerely yours,

Shahin Mahmud

Assistant Professor

Department of Biotechnology and Genetic Engineering

Mawlana Bhashani Science and Technology University, Santosh, Tangail-1902, Bangladesh.

Email: [shahin018mbstu@gmail.com](mailto:shahin018mbstu@gmail.com)

**Responses to the honorable reviewers’ s comments**

**Honorable Reviewer 1:**

R2 revision demonstrates several improvements compared with the previous version; however, the revisions are still not fully sufficient from a methodological standpoint. While the authors have clarified several technical aspects of the modeling framework and expanded the methodological description, some of the fundamental statistical concerns remain unresolved.

| **SL.** | **Comments/Questions/Instruction/Guidelines** | **Responses of the Authors** |
| --- | --- | --- |
| **1.** | One positive improvement is the clearer description of the modeling framework. The authors now explain the Graph Neural Network architecture in greater detail and specify the training, validation, and testing split of the dataset using a 60–20–20 partition. In addition, the implementation of nested 5-fold cross validation for hyperparameter tuning and model evaluation represents an appropriate strategy to reduce information leakage and obtain a more unbiased estimate of model performance. These methodological clarifications improve transparency and reproducibility. However, the primary methodological concern persists because the dependent variable used in the model remains a constructed composite “propensity score” rather than an observed biological or clinical outcome. This score is generated by combining normalized gene expression magnitude, graph-topological centrality measures, and pathway membership information. Consequently, the model effectively learns to reproduce a score that is derived from the same features used as model inputs. In such cases, high predictive performance metrics, such as the reported AUC values around 0.93 and R² values around 0.85, may reflect internal consistency rather than genuine predictive capability. Therefore, the reported performance should be interpreted cautiously because the model is not predicting an independent biological outcome but rather a derived internal metric. | We agree with you that the composite “propensity score” is a constructed metric derived from related input features. We have further clarified in the manuscript that the model is designed to reproduce and refine an integrative prioritization score, rather than predict an independent biological or clinical outcome. Accordingly, we explicitly state that performance metrics (AUC, R²) reflect internal consistency and ranking capability, and interpreted as a gene prioritization model. We have clarified these in the method 2.1 section and discussion section. |
| **2.** | Another issue concerns the conversion of the continuous propensity score into a binary classification using a threshold of 0.5 after sigmoid normalization. Although the authors describe this step as a secondary interpretive analysis, the justification for this threshold is not clearly explained. Without a biological or statistically optimized decision threshold, the binarization process appears somewhat arbitrary and may influence the ROC-based evaluation metrics. In predictive modeling studies, threshold selection typically requires either clinical reasoning or optimization methods such as Youden index or cost-sensitive decision rules. | The use of a 0.5 threshold after sigmoid normalization was chosen based on the classification threshold. In both classification strategies, whether it is ROC-AUC or PR, 0.5 determines the discrimination boundary (Fig. 3E and F). Higher values than 0.5 mean better discrimination, and lower than 0.5 mean lower discrimination. Due to a classification-based study, it is more logical to choose a pre-defined boundary.  Though this step was intended solely as a secondary interpretative aid to discriminate between the low-priority gene class and the high-priority gene class. The genes that were in higher rank, importantly, the top 20-ranked genes, consistently exhibited high predicted scores (≥0.83). As shown in the table, the separation between high-priority and lower-priority genes is substantial, indicating that the selection of a 0.5 cutoff did not influence the prioritization of key targets.    Therefore, using this cutoff, we separated the top and bottom gene classes. We agree that threshold optimization methods such as the Youden index or cost-sensitive approaches are valuable, particularly in classification-focused studies. However, the use of a 0.5 threshold does not affect the robustness of our findings. Nonetheless, we have clarified this rationale in Section 2.1 of the method section of the manuscript to avoid any ambiguity.  “Due to the complexity of several layers of input data in the model, we did not consider the Youden index or cost-sensitive approaches in this case; rather, we chose a simplified classification boundary threshold (≥0.5 after sigmoid normalization) to remove background genes from more valuable genes.”  However, in future studies, we will try to apply your suggestion on using the Youden index or cost-sensitive approaches to develop new models. |
| **3.** | The validation strategy also shows partial improvement but remains limited. The authors expanded the variant validation step using data from the cBioPortal database and analyzed a large cohort of gastric cancer samples across multiple studies. While this strengthens the biological context of the findings, it still cannot be considered a true external validation. The training data sources and validation data originate from the same general genomic data ecosystem, meaning that the independence between training and validation datasets may not be sufficient to demonstrate real model generalizability. | Sorry for the confusion. The training data did not integrate any variants themselves. In Table 2, the 8^th^ column incorporates mutation frequency to select highly mutated genes TP53, ERBB2, APC, and CDH1.  In this case, we explained the percentages of variants available for each gene in cBioPortal, not which variants or even which class of variants.  Therefore, this first selection strategy was a gene prioritization strategy from the cBioPortal database based on how many percentages of variants available in the database, and later in the study, the variant classification strategy was completely independent, based on variants only and not associated with the training dataset or model.  Hence, in summary, in the beginning, the validation was for genes based on the percentage of mutation frequency of each gene, and later, the validation was from the variants themselves for variants only, not for genes. These datasets and strategies are completely different from each other.  Hope we have clarified the issues and removed confusion. We have now clarified this issue in the discussion section, where variant classification was discussed.  “This cBioPortal validation was completely different from the initial gene validation based on mutation frequency score, as in this case, all the variants themselves were validated from the database.” |
| 4. | Regarding statistical inference, the authors indicate that FDR-adjusted p-values were used for identifying differentially expressed genes. This is an appropriate correction for multiple comparisons. However, the manuscript still reports statistical significance primarily using the conventional p<0.05 threshold in several sections, which creates ambiguity regarding whether adjusted p-values or nominal p-values are being interpreted. If FDR correction was applied, the adjusted p-values should consistently be reported and used as the main inferential criterion. | Thank you for pointing out these discrepancies. We have reviewed the p-values and, in addition to differentially expressed genes, we have revised the manuscript to ensure that FDR-adjusted p-values are consistently reported and interpreted as the primary criterion for statistical significance by removing ambiguity associated with nominal p-value thresholds. |
| 5. | In the survival analysis section, Kaplan–Meier analysis is presented to assess associations between gene expression and patient survival. While this provides an initial descriptive assessment, the absence of multivariable Cox proportional hazards modeling limits the clinical interpretability of the results. Without adjusting for potential confounding variables such as tumor stage, age, or treatment factors, the survival associations remain exploratory rather than predictive. | We have presented multivariable Cox proportional hazards modeling and adjustment for clinical covariates across different cancer stages in detail in Supplementary Table S11 and explained them in Section 3.14. |
| 6. | The variant prioritization strategy also raises methodological considerations. The use of seven computational prediction tools to classify missense variants provides a consensus-based filtering strategy that may improve sensitivity; however, these tools are not statistically independent and often rely on overlapping structural and evolutionary features. As a result, agreement among tools should not be interpreted as independent validation. Ideally, inter-tool agreement statistics or external functional validation would strengthen the credibility of these predictions. | Thank you for this important suggestion. We have revised the text to clarify that agreement among tools reflects consensus rather than independent validation, and we highlight the importance of inter-tool agreement metrics in the limitations section of the discussion. |
| 7. | Furthermore, several analyses in the manuscript, including structural energy estimation, electrostatic analysis, and k-means clustering of atomic-level structural features, should be interpreted as exploratory computational analyses. While they provide potentially interesting mechanistic insights, they do not constitute direct biological validation. These analyses generate hypotheses regarding structural instability or functional perturbation, but the causal relationship between the predicted structural changes and gastric cancer pathogenesis remains inferential. | We agree that analyses such as structural energy estimation, electrostatic profiling, and clustering should be considered exploratory. The manuscript has been revised to clearly position these results as hypothesis-generating and provide supporting evidence, and we ensured that they do not sound like direct biological validation. |
| 8. | In summary, the R2 revision improves the clarity of the methodology and addresses several technical concerns raised in the previous review. The description of the neural network architecture, cross-validation framework, and computational workflow is now considerably more transparent. Nevertheless, the central statistical limitations remain partially unresolved. The model still relies on a derived dependent variable rather than an independent biological outcome, the classification threshold lacks clear justification, true external validation is absent, and several inferential conclusions remain exploratory. Therefore, while the revisions represent meaningful progress and partially address previous concerns, they are not entirely sufficient from a strict methodological and statistical perspective. Additional external validation, clearer justification of modeling choices, and stronger statistical inference would substantially strengthen the scientific reliability of the study. | The manuscript now consistently emphasizes that the work represents a computational prioritization and integrative analysis framework rather than any biological validation strategy.  We have further clarified the use of the classification threshold for the model based on the discrimination boundary. Moreover, we also clarified the validation of gene and variant strategies. They confirmed that they were from the cBioPortal database, but were not the same datasets. Overall, the mutation frequency score was used for gene validation, and the variant datasets themselves were used for variant validation as an external validation because the primary source of variants was the NCBI ClinVar database, not the cancer-specific cBioPortal database.  We have now provided a clearer justification of the model classification strategy, model choices, gene validation, and variant validation strategies. We have also revised statistical inference based on your suggestion and responded to each of the queries. Hope these revisions addressed all of your concerns, explicit the limitations more clearly, and significantly improved our manuscript. |
| We are grateful for your valuable feedback, which provided much-needed clarity on our methodology and the model's core contributions and enhanced the overall quality of the manuscript. | | |

**Honorable Reviewer 2:**

The manuscript has improved substantially in clarity, organization, and reporting transparency after revision. The authors have addressed many of the concerns raised in prior review rounds, especially with respect to manuscript structure, clearer method descriptions, inclusion of code availability, tempering of causal language, and better explanation of the graph neural network framework. While some methodological limitations remain, they are now more openly acknowledged, and the study is more appropriately framed as a computational prioritization resource. Given that this is the second round of review, I would support acceptance, while still noting the following comments for the authors and editor.

**Major Comments**

| **SL.** | **Comments/Questions/Instructions/Guidelines** | **Responses of the Authors** |
| --- | --- | --- |
| 1. | The revised manuscript is clearer in defining the GNN objective, and the authors now explain that the model predicts a composite propensity score integrating expression dysregulation, network centrality, and pathway membership. This improves transparency. However, the model still appears to predict a score constructed from features closely related to the same inputs, so the framework should be interpreted primarily as a **prioritization model** rather than as an independently predictive biological model. | Thank you for your suggestion. We have clarified that the model predicts a composite propensity score derived from related input features and explicitly frame the method as a prioritization model rather than an independently predictive biological model in Section 2.1 of the Method. |
| 2. | The manuscript now more explicitly acknowledges that the study is focused on **prioritization rather than novel discovery**, which is an appropriate clarification. This is important because the principal highlighted genes, particularly **TP53, CDH1, and APC**, are already well-established in gastric cancer. The revised framing is more accurate, but the conclusions should continue to avoid implying that the framework uncovers fundamentally new driver biology. | We acknowledge this point and have revised the conclusion to consistently emphasize that the study focuses on prioritization rather than discovery. |
| 3. | The added explanation of nested cross-validation and gene-level partitioning strengthens the description of the validation framework. However, the integrated use of partially overlapping resources such as GEPIA2, STRING, cBioPortal, and KEGG still means that the reported performance should be interpreted with caution. The manuscript is improved on this point, but the results remain more supportive of **internal ranking robustness** than of broad biological generalizability. | We appreciate this observation, and to overcome this partially overlapping resources matter, we have come to a common ground with a propensity score for each. Therefore, we have clarified that the reported performance was based on the primary metrics: propensity score and secondary metrics: classification-based ROC.  We have also clarified that results primarily demonstrate internal ranking robustness rather than broad biological generalization in the discussion section. |
| 4. | The baseline comparisons and clarification of supervised versus semi-supervised elements improve the methodological presentation. Still, the biological interpretation of the “predicted score” versus “importance score” remains somewhat abstract. The current revision is acceptable, but the manuscript should continue to emphasize that these scores are **computational prioritization measures** and not direct biological effect estimates. | We have further clarified the distinction and explicitly state that both scores represent computational prioritization measures, not direct biological effect estimates, in the discussion section. |
| 5. | The survival analyses have been improved by including false discovery rate correction, hazard ratios, and explicit acknowledgment that associations are correlative. Nevertheless, the analyses still lack adjustment for clinical covariates such as stage or treatment, meaning the biological interpretation remains limited. Thus, the statistical reporting is improved but the inferential strength remains modest. | We have incorporated clinical covariates (different stages of cancer) in the Supplementary Table S11. |
| 6. | Overall, the revised paper is stronger as a computational resource and integrative prioritization study than as a mechanistic or predictive framework. I think the manuscript is now acceptable in that context, but the interpretation should remain aligned with that level of evidence | We appreciate your recommendations for acceptance and ensured that the claims in our study are consistent with the level of evidence. We have described the predictive framework as a prioritization of genes by predicting the score for each gene based on differentially expressed genes, protein-protein interactions, pathway genes, etc. We also clarified that the mechanistic framework is not proposed based on the model only, but based on the whole system-level study, and we have therefore used the word proposed for the framework, and also clarified that the findings could be an integrative source of further experimental studies. |

**Minor Comments**

| **SL.** | **Comments/Questions/Instructions/Guidelines** | **Responses of the Authors** |
| --- | --- | --- |
| 1. | The writing is improved, but a final language edit would still help in a few places to correct grammar, article usage, and sentence flow. | The manuscript has undergone an additional careful editing to improve grammar, article usage, and overall sentence flow using the Grammarly tool. |
| 2. | Some sections remain long and slightly repetitive, especially where multiple computational tools converge on the same conclusion. A final pass for conciseness would improve readability further. | We have performed a thorough revision to reduce redundancy, particularly in sections where multiple computational approaches support similar conclusions. These sections have been streamlined to improve clarity and readability. |
| 3. | Figure legends have improved, but a few dense figures would still benefit from even more explicit labeling of axes, color scales, and panel-specific interpretation. | We have tried our best to maintain proper figure legends to ensure clearer interpretation. Axes, color scales, and panel-specific descriptions have been reviewed and modified, especially for complex figures 9-11. |
| 4. | Terminology should remain fully consistent throughout the manuscript, especially for terms such as “variant,” “missense SNP,” “oncogenic,” “tumor suppressor,” “importance score,” and “predicted score.” | We have ensured the standardization of terminology throughout the manuscript, ensuring consistent usage of key terms such as “variant,” “missense SNP,” “oncogenic,” “tumor suppressor,” “importance score,” and “predicted score.”  We have consistently used “variants” when referring broadly to genetic alterations, particularly in the discussion section, and reserved “missense SNPs” to denote amino-acid-changing SNPs only. Additionally, we maintained “oncogenic” only during pathogenic SNP classification in relation to cancer; otherwise, we have particularly defined the word “tumor suppressor” for tumor suppressor proteins.  Furthermore, the terminology importance score is only used when data is derived from the features, and the terminology predicted score is used when the score is derived from the model. |
| 5. | The manuscript now includes code availability, which is a major strength. The final version should ensure that the repository link, software versions, seeds, and any required input formatting are easy for readers to follow. | We have provided the repository link in the data availability section of the manuscript to ensure clarity and usability. The software versions, random seeds, and input data formatting requirements have been properly guided in the requirements.txt and README.md files at the repository link: <https://github.com/tanjida255/GNNHub.git>. |
| Thank you for the insightful suggestions, which have helped us further improve the clarity and consistency in our manuscript. | | |
