# Supplementary material for: Computational screening of oncogenic genetic variations in tumor suppressor proteins driving gastric cancer pathogenesis
Source: PLoS One. 2026 Sep 17;21(9):e0358440. doi: 10.1371/journal.pone.0358440 (PMC13585236; doi:10.1371/journal.pone.0358440)
Supplement: Supplementary file 10 [file pone.0358440.s021.docx]

**Responses to the honorable reviewers’ s comments**

**Reviewer 1:**

The authors have made a substantial effort to address the concerns raised during the previous rounds of review. Compared with the earlier versions, the manuscript has been significantly strengthened, particularly with respect to the transparency of the computational workflow and the methodological description. The Graph Neural Network architecture is now described in considerably greater detail, the training, validation, and testing strategy is clearly presented, nested cross-validation has been implemented, and additional information regarding data partitioning and measures to reduce information leakage has been incorporated. The statistical methodology is now more transparent and reproducible than in the previous submissions. Furthermore, the authors have clarified the distinction between the primary regression framework and the secondary classification analysis, expanded the justification for the computational prediction tools employed, improved the description of multiple testing correction, and substantially enhanced the Discussion by acknowledging several methodological limitations. The data availability statement has also been revised in accordance with the journal's requirements. Overall, the manuscript has undergone considerable methodological improvement, and the majority of the previous statistical concerns have been satisfactorily addressed.

| **SL.** | **Comments/Questions/Instruction/Guidelines** | **Responses of the Authors** |
| --- | --- | --- |
| **1.** | One methodological issue, however, still deserves a clearer acknowledgment rather than additional analyses. The Graph Neural Network is trained to predict a researcher-derived composite propensity score that integrates gene expression, network topology, and pathway information rather than an independent biological or clinical outcome. Consequently, the reported predictive performance metrics should be interpreted as measures of the model's ability to prioritize biologically relevant candidate genes within the constructed framework rather than as evidence of true clinical prediction or external prognostic performance. Although the authors have substantially clarified this modeling strategy, explicitly emphasizing this distinction in the Discussion and Conclusions would prevent potential overinterpretation of the reported predictive accuracy and further improve the scientific rigor of the manuscript. | We have revised the Discussion and Conclusion to explicitly state that the Graph Neural Network was trained to predict a composite propensity score integrating gene expression, network topology, and pathway information, rather than an independent clinical outcome. Accordingly, we have clarified that the reported performance metrics reflect the model's ability to prioritize biologically relevant candidate genes within this framework, rather than true clinical predictive or prognostic accuracy. The corresponding revisions have been incorporated into the Discussion and Conclusion sections of the manuscript. |
| **Comments from the honourable reviewer:**  Overall, I believe the authors have responded carefully and constructively to the reviewers' comments, and the manuscript has improved substantially during the revision process. The remaining concern is primarily one of interpretation rather than methodology and can be adequately addressed through a minor textual clarification. **Therefore, I consider the manuscript suitable for publication after minor revision.**  **Author response:**  We sincerely appreciate your constructive suggestions to acknowledge the minor issue in the discussion and conclusion sections of our manuscript for more clarification. | | |
