# Supplementary material for: Computational screening of oncogenic genetic variations in tumor suppressor proteins driving gastric cancer pathogenesis
Source: PLoS One. 2026 Sep 17;21(9):e0358440. doi: 10.1371/journal.pone.0358440 (PMC13585236; doi:10.1371/journal.pone.0358440)
Supplement: Peer review history

# Peer review: Decision Letter 0

**DOI:** 10.1371/journal.pone.0358440.r001

**Authors:** Amr Ahmed El-Arabey

19 Jan 2026

Dear Dr. Mahmud,

Thank you for submitting your manuscript to PLOS ONE. After careful consideration, we feel that it has merit but does not fully meet PLOS ONE’s publication criteria as it currently stands. Therefore, we invite you to submit a revised version of the manuscript that addresses the points raised during the review process.

Please submit your revised manuscript by Mar 05 2026 11:59PM. If you will need more time than this to complete your revisions, please reply to this message or contact the journal office at plosone@plos.org. When you're ready to submit your revision, log on to https://www.editorialmanager.com/pone/ and select the 'Submissions Needing Revision' folder to locate your manuscript file.

- A letter that responds to each point raised by the academic editor and reviewer(s). You should upload this letter as a separate file labeled 'Response to Reviewers'.
- A marked-up copy of your manuscript that highlights changes made to the original version. You should upload this as a separate file labeled 'Revised Manuscript with Track Changes'.
- An unmarked version of your revised paper without tracked changes. You should upload this as a separate file labeled 'Manuscript'.

If you would like to make changes to your financial disclosure, please include your updated statement in your cover letter. Guidelines for resubmitting your figure files are available below the reviewer comments at the end of this letter.

If applicable, we recommend that you deposit your laboratory protocols in protocols.io to enhance the reproducibility of your results. Protocols.io assigns your protocol its own identifier (DOI) so that it can be cited independently in the future. For instructions see: https://journals.plos.org/plosone/s/submission-guidelines#loc-laboratory-protocols. Additionally, PLOS ONE offers an option for publishing peer-reviewed Lab Protocol articles, which describe protocols hosted on protocols.io. Read more information on sharing protocols at https://plos.org/protocols?utm_medium=editorial-email&utm_source=authorletters&utm_campaign=protocols.

We look forward to receiving your revised manuscript.

Kind regards,

Amr Ahmed El-Arabey

Academic Editor

PLOS One

**Journal Requirements:**

1. When submitting your revision, we need you to address these additional requirements.

Please ensure that your manuscript meets PLOS ONE's style requirements, including those for file naming. The PLOS ONE style templates can be found at

https://journals.plos.org/plosone/s/file?id=wjVg/PLOSOne_formatting_sample_main_body.pdf and

https://journals.plos.org/plosone/s/file?id=ba62/PLOSOne_formatting_sample_title_authors_affiliations.pdf

2. Please note that PLOS One has specific guidelines on code sharing for submissions in which author-generated code underpins the findings in the manuscript. In these cases, we expect all author-generated code to be made available without restrictions upon publication of the work. Please review our guidelines at https://journals.plos.org/plosone/s/materials-and-software-sharing#loc-sharing-code and ensure that your code is shared in a way that follows best practice and facilitates reproducibility and reuse.

3. Please upload a new copy of Figures 2, 3, 4, 7, 9, 10, 11, 12, 13, 15 and 17, as the detail is not clear. Please follow the link for more information:  https://journals.plos.org/plosone/s/figures

4. Please include captions for your Supporting Information files at the end of your manuscript, and update any in-text citations to match accordingly. Please see our Supporting Information guidelines for more information: http://journals.plos.org/plosone/s/supporting-information.

5. If the reviewer comments include a recommendation to cite specific previously published works, please review and evaluate these publications to determine whether they are relevant and should be cited. There is no requirement to cite these works unless the editor has indicated otherwise. 

[Note: HTML markup is below. Please do not edit.]

Reviewers' comments:

**Comments to the Author**

1. Is the manuscript technically sound, and do the data support the conclusions?

Reviewer #1: Yes

Reviewer #2: Yes

**********

2. Has the statistical analysis been performed appropriately and rigorously?

Reviewer #1: No

Reviewer #2: Yes

**********

3. Have the authors made all data underlying the findings in their manuscript fully available?

The PLOS Data policy

Reviewer #1: Yes

Reviewer #2: Yes

**********

4. Is the manuscript presented in an intelligible fashion and written in standard English?

Reviewer #1: Yes

Reviewer #2: Yes

**********

**Reviewer #1:**  This study presents an ambitious and interdisciplinary computational framework aimed at elucidating oncogenic genetic variations in tumor suppressor proteins involved in gastric cancer pathogenesis. While the topic is timely and the integration of multi-layered bioinformatics and deep learning approaches is noteworthy, the manuscript in its current form exhibits substantial methodological and statistical limitations that undermine the robustness, interpretability, and translational value of its conclusions.

First and foremost, although a graph neural network (GNN) model is central to the study, the statistical and conceptual definition of the model’s prediction task remains insufficiently specified. It is unclear what exact biological or clinical outcome the model is trained to predict and how this outcome is operationalized. The simultaneous reporting of regression-based metrics (MSE, R²) and classification-based metrics (AUC-ROC) without a clearly defined dependent variable introduces methodological ambiguity. In particular, the use of AUC-ROC implies a binary classification framework, yet no explicit definition of class labels or decision thresholds is provided. This lack of clarity makes it difficult to assess whether the reported high performance values genuinely reflect predictive accuracy or stem from an ill-defined modeling objective.

Furthermore, the train–validation–test split (60:20:20), although standard in machine learning practice, does not adequately address the issue of data dependency inherent in the integrated use of GEPIA2, cBioPortal, STRING, and KEGG databases. These resources are not statistically independent and share overlapping biological knowledge, which raises serious concerns about information leakage. Consequently, the reported high performance metrics (e.g., AUC values exceeding 0.93) may be overly optimistic. From a biostatistical standpoint, the absence of a truly independent external validation cohort or more robust resampling strategies (such as nested cross-validation) substantially weakens the credibility of the model’s generalizability.

Another major methodological shortcoming lies in the extensive use of multiple hypothesis testing without any formal control for multiplicity. The study evaluates thousands of genes, hundreds of variants, and numerous downstream computational outputs, yet no false discovery rate (FDR) or family-wise error correction is applied. This omission is particularly problematic in survival analyses, where statistical significance is reported solely as p < 0.05, without accompanying effect size estimates (e.g., hazard ratios with confidence intervals) or adjustment for multiple comparisons. As a result, the risk of false-positive findings is substantial, and the clinical relevance of the reported associations remains uncertain.

The classification of “high-risk” missense SNPs based on consensus across seven prediction tools appears intuitively appealing; however, it implicitly assumes independence among these tools, which is not the case. Many of the employed algorithms rely on overlapping sequence conservation and structural features, thereby inflating concordance rates. Without a formal assessment of inter-tool agreement (e.g., using kappa statistics or latent class modeling), it is unclear whether consensus truly reflects biological risk or merely methodological redundancy. Addressing this issue would significantly strengthen the inferential rigor of the variant prioritization strategy.

Similarly, downstream analyses of protein stability, energy minimization, titration behavior, and post-translational modification sites are presented primarily in a descriptive manner. While these analyses suggest potential structural and functional perturbations, they lack a quantitative or causal framework linking computational outputs to biologically meaningful thresholds of functional impairment. For instance, changes in energy values or isoelectric points are reported without reference to established benchmarks indicating functional loss, limiting their interpretive value and weakening translational claims.

From a reporting perspective, the manuscript would benefit from a clearer separation between methods, results, and interpretation. In several sections, methodological justification is interwoven with result presentation, which obscures the distinction between empirical findings and analytical assumptions. A more disciplined structure would enhance readability and allow readers to more clearly evaluate the strength of the evidence.

In conclusion, while the study addresses an important biological problem and employs a wide array of modern computational tools, its current methodological framework does not yet meet the standards required for robust statistical inference and clinical interpretation. Clarifying the modeling objective, strengthening validation strategies, rigorously controlling for multiple testing, quantifying effect sizes, and improving causal interpretation of computational findings are essential steps to substantially improve the scientific reliability and impact of the work. With these revisions, the study has the potential to evolve from a broad exploratory analysis into a methodologically sound contribution to gastric cancer research.

**Reviewer #2:**  Overall Assessment

1. The manuscript presents an ambitious and extensive computational systems-level analysis integrating gene expression, mutation frequency, graph neural networks, and multiple structural bioinformatics tools to study tumor suppressor disruption in gastric cancer (GC).

2. The study compiles a large number of analyses and databases and attempts to link mutation burden, protein structural instability, regulatory disruption, and survival associations.

3. While the breadth of analysis is impressive, the manuscript currently suffers from methodological over-claiming, insufficient validation, unclear modeling objectives, and limited biological grounding, which substantially weakens its interpretability and reproducibility.

4. The work would benefit from substantial revision to clarify the analytical framework, reduce redundancy, and align conclusions more closely with the presented evidence.

Major Comments

1. Conceptual Novelty and Framing

The manuscript claims a “systems-level” and “deep learning–based” framework; however:

The biological novelty is limited, as TP53, CDH1, and APC are already well-established drivers in gastric cancer.

The study primarily reconfirms known biology using computational screening rather than uncovering new mechanistic insights.

The authors should explicitly clarify:

What new biological insight emerges beyond known mutation frequencies and pathway involvement?

How this framework advances beyond prior integrative studies using TCGA, cBioPortal, and structural prediction tools.

2. Graph Neural Network (GNN) Model – Purpose and Interpretation

The GNN framework is insufficiently justified conceptually:

It is unclear what biological question the model is predicting (classification? regression? prioritization?).

The definition of “predicted score” and “importance score” lacks interpretability in biological terms.

Performance metrics (MSE, R², AUC-ROC) are reported, but:

There is no baseline comparison (e.g., random forest, logistic regression, network centrality alone).

High AUC values may reflect class imbalance or circular feature selection (genes pre-selected from known GC pathways).

The authors should:

Explicitly define the target variable used for training.

Clarify whether the task is supervised or semi-supervised.

Include ablation or baseline models to justify the use of a GNN.

3. Risk of Circular Reasoning

Multiple steps risk circular inference:

Genes are selected from GC databases → network centrality is computed → high-centrality GC genes are “predicted” as important.

TP53 being identified as the top hub gene is expected and does not validate the model.

The manuscript should:

Explicitly acknowledge this limitation.

Demonstrate whether the model can identify less obvious candidates or stratify within known genes.

4. Structural and Energetic Analyses – Overinterpretation

Structural stability predictions (I-Mutant, MUpro, Missense3D, HOPE) are:

In silico predictions only, yet are frequently described in causal language (e.g., “catastrophic instability,” “severe disruption”).

Energy minimization and hydropathy analyses lack:

Statistical thresholds

Replicates

Sensitivity analysis

The authors should:

Temper causal language.

Explicitly state that these analyses are hypothesis-generating.

Avoid equating predicted destabilization with functional loss.

5. Unsupervised Machine Learning on Atomic Coordinates

The k-means clustering of atomic features is not sufficiently justified:

Feature engineering is vaguely described.

Cluster number selection is not explained.

No quantitative validation (e.g., silhouette score, stability analysis) is provided.

Claims that cluster disruption equates to loss of tumor suppressor function are not supported by experimental evidence.

This section should either be:

Significantly simplified, or

Reframed as exploratory with strong caveats.

6. Survival and Expression Analyses

Survival analyses using GEPIA2 and KM-Plotter:

Do not control for clinical covariates (stage, subtype, treatment).

Risk overstating associations between expression and outcome.

The manuscript should clarify:

That survival associations are correlative, not causal.

Whether multiple testing correction was applied.

7. Scope and Length

The manuscript is excessively long and methodologically dense.

Many analyses (PTMs, titration curves, energetic profiles) repeat the same conclusion: “mutation disrupts protein stability.”

The authors should:

Consolidate overlapping analyses.

Move secondary or confirmatory analyses to Supplementary Materials.

Minor Comments

Clarity and Writing

The manuscript frequently uses overly assertive language (“proves,” “confirms,” “catastrophic”) inappropriate for computational predictions.

Several sections repeat background information that could be condensed.

Some sentences are excessively long and should be simplified for clarity.

Figures and Tables

Several figures (e.g., Figs. 10–12) are visually dense and difficult to interpret without extensive captions.

Figure legends should:

Clearly define axes, units, and color scales.

Avoid repeating Results text verbatim.

Terminology and Consistency

“Missense SNPs” is used interchangeably with “variants”; consider consistent terminology.

“Oncogenic” is used for tumor suppressor loss-of-function variants; clarify usage to avoid confusion.

Data Availability and Reproducibility

While tools and databases are listed, code availability is not addressed.

For a computational study of this scope, the authors should:

Provide scripts or workflows (e.g., GitHub).

Specify software versions and random seeds.

Specific Technical Questions for the Authors

How were negative examples defined for the GNN?

Were node features independent of pathway annotations used later for interpretation?

How sensitive are results to:

Different train/test splits?

Removal of TP53 from the network?

Why were only three tumor suppressors carried forward despite identifying 1,886 candidate genes?

**********

what does this mean?). If published, this will include your full peer review and any attached files.

If you choose “no”, your identity will remain anonymous but your review may still be made public.

**Do you want your identity to be public for this peer review?**  For information about this choice, including consent withdrawal, please see our Privacy Policy

Reviewer #1: No

Reviewer #2: **Yes:**  Somnath Tagore

**********

[NOTE: If reviewer comments were submitted as an attachment file, they will be attached to this email and accessible via the submission site. Please log into your account, locate the manuscript record, and check for the action link "View Attachments". If this link does not appear, there are no attachment files.]

To ensure your figures meet our technical requirements, please review our figure guidelines: https://journals.plos.org/plosone/s/figures

You may also use PLOS’s free figure tool, NAAS, to help you prepare publication quality figures: https://journals.plos.org/plosone/s/figures#loc-tools-for-figure-preparation.

NAAS will assess whether your figures meet our technical requirements by comparing each figure against our figure specifications.

# Author comment: Author response to Decision Letter 1

**DOI:** 10.1371/journal.pone.0358440.r002

11 Feb 2026

12 February, 2026

Amr Ahmed El-Arabey

Academic Editor

PLOS One

Dear Editor,

Special gratitude to you for allowing us to resubmit a more revised and updated version of our manuscript entitled "Computational screening of oncogenic genetic variations in tumor suppressor proteins driving gastric cancer pathogenesis" (Manuscript ID: PONE-D-25-52287, EMID:715c02d22fb7297f) to the PLOS ONE journal as an original research article. We have organized the manuscript based on the reviewers’ comments and journal requirements. In response to valuable suggestions, we have modified, revised, organized, and corrected the manuscripts. We have appropriately addressed all queries of Reviewers in the response letter, line by line. We also followed journal style, providing revised Figures 2, 3, 4, 7, 9, 10, 11, 12, 13, 15, and 17, along with sharing code utilized in this study. Additionally, we have included captions for Supporting Information files at the end of our manuscript and updated any in-text citations to match accordingly. We hope our response below will successfully address the reviewer’s specific points and improve the manuscript. Please let us know if you have any additional questions or comments. Finally, we would like to express our thanks and gratitude for your kind cooperation.

Sincerely yours,

Shahin Mahmud

Assistant Professor

Department of Biotechnology and Genetic Engineering

Mawlana Bhashani Science and Technology University, Santosh, Tangail-1902, Bangladesh.

Email: shahin018mbstu@gmail.com

Responses to the honorable reviewers’ s comments

Honorable Reviewer 1:

This study presents an ambitious and interdisciplinary computational framework aimed at elucidating oncogenic genetic variations in tumor suppressor proteins involved in gastric cancer pathogenesis. While the topic is timely and the integration of multi-layered bioinformatics and deep learning approaches is noteworthy, the manuscript in its current form exhibits substantial methodological and statistical limitations that undermine the robustness, interpretability, and translational value of its conclusions.

Comments/Questions/Instruction/Guidelines:

First and foremost, although a graph neural network (GNN) model is central to the study, the statistical and conceptual definition of the model’s prediction task remains insufficiently specified. It is unclear what exact biological or clinical outcome the model is trained to predict and how this outcome is operationalized. The simultaneous reporting of regression-based metrics (MSE, R²) and classification-based metrics (AUC-ROC) without a clearly defined dependent variable introduces methodological ambiguity. In particular, the use of AUC-ROC implies a binary classification framework, yet no explicit definition of class labels or decision thresholds is provided. This lack of clarity makes it difficult to assess whether the reported high performance values genuinely reflect predictive accuracy or stem from an ill-defined modeling objective.

Responses of the Authors:

Thank you for highlighting this important point regarding the conceptual clarity of the prediction task and the use of evaluation metrics for GNN model. We fully agree that an explicit definition of the model’s learning objective is essential for methodological transparency and interpretability. We have substantially revised Section 2.1 to clearly define the inputs and the biological outcome being modeled and to justify the combined use of regression and classification-based performance measures.

“At first, the GNN is trained to predict a propensity score based on the relevance for 3 different sets of genes.

(1) Mutation-prone Differentially Expressed Genes.

(2) Genes from the protein-protein interaction network.

(3) Gastric cancer (GC) pathway-specific genes. This represents the integrated likelihood that a gene contributes to gastric cancer pathogenesis due to mutations, expression dysregulation, and network-level functional importance. A continuous propensity score is operationalized by combining normalized gene expression magnitude with multiple graph-topological centrality measures (degree, betweenness, and closeness) and, when available, pathway membership information. These biological components reflect the premise that genes that are both dysregulated and centrally positioned in protein interaction networks, along with being present in the GC pathway, are more likely to act as key disease drivers. The resulting composite score serves as the dependent variable in a regression framework and ranks the top genes. Therefore, the primary regression performance metrics were mean squared error (MSE), root mean squared error (RMSE), and coefficient of determination (R²).

Afterward, the classification metrics were used as a secondary interpretive analysis (AUC-ROC reporting) rather than the primary modeling objective. For this purpose, the continuous propensity scores were binarized using a biologically interpretable threshold (≥0.5 after sigmoid normalization), representing high-confidence versus low-confidence pathogenic candidates. This transformation allowed us to assess the model’s ability to discriminate high-risk oncogenic genes from background genes.” We have now explicitly stated the thresholding strategy, the rationale behind it, and the biological interpretation of the resulting classes in the revised manuscript.

The regression captures fine-grained biological risk ranking, while ROC and precision-recall analyses provide the discrimination performance to remove background genes for prioritization tasks. We also clarified that all reported metrics were computed on independent training, validation, and test partitions to ensure robustness. Figure 2C details the explanation now for the input data:

Figure 2C. GNN Model.

Overall, these revisions remove ambiguity regarding the learning objective and align the reported performance metrics with a clearly defined biological prediction task. The manuscript now explicitly frames the GNN as a continuous disease-driver prioritization model, ensuring that the reported high performance reflects genuine predictive capability rather than an ill-specified outcome.

Comments/Questions/Instruction/Guidelines:

Furthermore, the train–validation–test split (60:20:20), although standard in machine learning practice, does not adequately address the issue of data dependency inherent in the integrated use of GEPIA2, cBioPortal, STRING, and KEGG databases. These resources are not statistically independent and share overlapping biological knowledge, which raises serious concerns about information leakage. Consequently, the reported high performance metrics (e.g., AUC values exceeding 0.93) may be overly optimistic. From a biostatistical standpoint, the absence of a truly independent external validation cohort or more robust resampling strategies (such as nested cross-validation) substantially weakens the credibility of the model’s generalizability.

Responses of the Authors:

We appreciate your important concern regarding potential data dependency and the risk of optimistic performance estimation arising from integrated biological databases. In response, we have strengthened both the validation strategy and its methodological justification in the revised manuscript.

First, we now explicitly acknowledge that GEPIA2, STRING, cBioPortal, and KEGG are biologically interconnected resources and that naïve random splitting can introduce information leakage. To maintain and balance the highest accuracy and relevancy, we have removed all duplicates before feeding the input to the model, although the Torch library can maintain unique values as well. Then, we implemented a standard gene-level partitioning strategy in which all features and network relationships associated with a given gene are confined to prevent cross-set propagation of topological or annotation-derived information. This change is now clearly described in the revised Methods section.

Second, beyond the original train-validation-test division, we incorporated a nested 5-fold cross-validation (CV) strategy based on your suggestion, which maintains both hyperparameter tuning and unbiased test estimate with 2 loops: outer loop for model evaluation and inner loop for hyperparameter tuning (Method section 2.1).

Performance metrics are now reported as mean ± standard deviation across folds, providing a more conservative and statistically robust estimate of generalization performance: MSE, RMSE, R2 and AUC values (Table 1 and Supplementary File S1).

However, most importantly, the external biological validation independent of the model is confirmed from variants using cBioPortal mutation frequencies, survival associations, and literature-supported gastric cancer drivers. This step serves as a functional external benchmark rather than a purely computational split.

We believe these methodological enhancements directly addressed information leakage concerns and substantially strengthened the credibility and generalizability of the proposed GNN framework.

Comments/Questions/Instruction/Guidelines:

Another major methodological shortcoming lies in the extensive use of multiple hypothesis testing without any formal control for multiplicity. The study evaluates thousands of genes, hundreds of variants, and numerous downstream computational outputs, yet no false discovery rate (FDR) or family-wise error correction is applied. This omission is particularly problematic in survival analyses, where statistical significance is reported solely as p < 0.05, without accompanying effect size estimates (e.g., hazard ratios with confidence intervals) or adjustment for multiple comparisons. As a result, the risk of false-positive findings is substantial, and the clinical relevance of the reported associations remains uncertain.

Responses of the Authors:

At first, we have specifically adjusted p-values using the Benjamini–Hochberg false discovery rate (FDR) procedure to screen DEGs, with significance reported as FDR-adjusted p-values throughout the manuscript (< 0.05). This correction has been applied to differential expression filtering, and a p-value less than 0.05 to consider significance has been applied to all the analyses. The Methods section 2.5 now clearly details the correction strategy and reporting thresholds.

Second, for survival analyses, we no longer rely solely on nominal p-values. We now report hazard ratios (HRs) with 95% confidence intervals, alongside FDR-adjusted significance values and low and high expression cohort differences (months) (Result section 3.15). This provides both statistical robustness and clinically meaningful effect size interpretation.

These changes substantially reduce the risk of false-positive discovery, strengthen statistical credibility, and improve the clinical relevance of the reported associations.

Comments/Questions/Instruction/Guidelines:

The classification of “high-risk” missense SNPs based on consensus across seven prediction tools appears intuitively appealing; however, it implicitly assumes independence among these tools, which is not the case. Many of the employed algorithms rely on overlapping sequence conservation and structural features, thereby inflating concordance rates. Without a formal assessment of inter-tool agreement (e.g., using kappa statistics or latent class modeling), it is unclear whether consensus truly reflects biological risk or merely methodological redundancy. Addressing this issue would significantly strengthen the inferential rigor of the variant prioritization strategy.

Responses of the Authors:

Thank you for raising a fair point about whether these prediction tools are truly independent, but there is a strong case for why the consensus approach still works well. Yes, many of these algorithms do use similar information, such as evolutionary conservation and protein structure, but they process this information in very different ways using different mathematical methods, such as machine learning classifiers, hidden Markov models, likelihood ratio tests, and ensemble methods with distinct training datasets, weighting schemes, and decision boundaries.

What really matters is that this multi-tool consensus method has been tested extensively in real-world studies and consistently performs better than relying on any single tool alone. Studies show that when multiple tools agree, the predictions are much more likely to be correct, which is why this approach has become widely accepted and is recommended in clinical genetics guidelines from the ACMG variant classification guidelines [1].

The key point is that we are not trying to invent a new prediction method; rather, we are using a well-established strategy to narrow down which genetic variants are worth the time and expense of laboratory testing. Requiring seven tools to agree sets a high bar that helps avoid false alarms, making sure we focus our resources on the variants most likely to actually matter.

While your suggestion to formally measure how much these tools overlap is reasonable and could add useful information, the fact that this consensus approach has been proven reliable across countless genetics studies gives us good reason to trust it for identifying high-priority variants to investigate further [2–6].

We have now mentioned the utilization of ACMG variant classification guidelines in the 2.5 method section.

Comments/Questions/Instruction/Guidelines:

Similarly, downstream analyses of protein stability, energy minimization, titration behavior, and post-translational modification sites are presented primarily in a descriptive manner. While these analyses suggest potential structural and functional perturbations, they lack a quantitative or causal framework linking computational outputs to biologically meaningful thresholds of functional impairment. For instance, changes in energy values or isoelectric points are reported without reference to established benchmarks indicating functional loss, limiting their interpretive value and weakening translational claims.

Responses of the Authors:

We have presented biophysical shifts in protein stability and post-translational modification sites as structural and functional perturbations, which is a well-established practice in genomics studies [4,7,8]. However, we performed additional energetic profiles and titration behavior to provide comparative studies to estimate how much deviation could be observed due to a SNP in terms of energy and pI changes, respectively, rather than defining a functional loss.

The main motif of these methods was to estimate the statistically significant destabilization, altered charge distributions, or loss of regulatory modification sites compared to native structures. Such comparative frameworks are routinely employed in variant effect prediction and disease mutation studies.

Therefore, together, these strategies provide prioritization of SNPs rather than any definitive claims. Hence, these positions form our approach not as descriptive speculation but as a biologically grounded and community-accepted strategy for prioritizing high-impact pathogenic variants, consistent with best practices in computational genomics.

Comments/Questions/Instruction/Guidelines:

From a reporting perspective, the manuscript would benefit from a clearer separation between methods, results, and interpretation. In several sections, methodological justification is interwoven with result presentation, which obscures the distinction between empirical findings and analytical assumptions. A more disciplined structure would enhance readability and allow readers to more clearly evaluate the strength of the evidence.

Responses of the Authors:

We thank you for this valuable suggestion regarding manuscript structure. We have reorganized the manuscript to ensure a clear separation between Methods, Results, and Discussion, relocating all methodological justifications exclusively to the Methods section and reserving the Results for empirical findings only. Interpretative insights are now presented in the Discussion, improving readability and enabling a more transparent evaluation of analytical rigor and evidence strength.

Comments/Questions/Instruction/Guidelines:

In conclusion, w

Attachment Submitted filename: Response to Reviewers.pdf

# Peer review: Decision Letter 1

**DOI:** 10.1371/journal.pone.0358440.r003

**Authors:** Amr Ahmed El-Arabey

18 Feb 2026

Dear Dr. Mahmud,

Thank you for submitting your manuscript to PLOS ONE. After careful consideration, we feel that it has merit but does not fully meet PLOS ONE’s publication criteria as it currently stands. Therefore, we invite you to submit a revised version of the manuscript that addresses the points raised during the review process.

Please submit your revised manuscript by Apr 04 2026 11:59PM. If you will need more time than this to complete your revisions, please reply to this message or contact the journal office at plosone@plos.org. When you're ready to submit your revision, log on to https://www.editorialmanager.com/pone/ and select the 'Submissions Needing Revision' folder to locate your manuscript file.

- A letter that responds to each point raised by the academic editor and reviewer(s). You should upload this letter as a separate file labeled 'Response to Reviewers'.
- A marked-up copy of your manuscript that highlights changes made to the original version. You should upload this as a separate file labeled 'Revised Manuscript with Track Changes'.
- An unmarked version of your revised paper without tracked changes. You should upload this as a separate file labeled 'Manuscript'.

If you would like to make changes to your financial disclosure, please include your updated statement in your cover letter. Guidelines for resubmitting your figure files are available below the reviewer comments at the end of this letter.

If applicable, we recommend that you deposit your laboratory protocols in protocols.io to enhance the reproducibility of your results. Protocols.io assigns your protocol its own identifier (DOI) so that it can be cited independently in the future. For instructions see: https://journals.plos.org/plosone/s/submission-guidelines#loc-laboratory-protocols. Additionally, PLOS ONE offers an option for publishing peer-reviewed Lab Protocol articles, which describe protocols hosted on protocols.io. Read more information on sharing protocols at https://plos.org/protocols?utm_medium=editorial-email&utm_source=authorletters&utm_campaign=protocols.

We look forward to receiving your revised manuscript.

Kind regards,

Amr Ahmed El-Arabey

Academic Editor

PLOS One

Journal Requirements:

If the reviewer comments include a recommendation to cite specific previously published works, please review and evaluate these publications to determine whether they are relevant and should be cited. There is no requirement to cite these works unless the editor has indicated otherwise.

[Note: HTML markup is below. Please do not edit.]

Reviewers' comments:

Reviewer's Responses to Questions

**Comments to the Author**

Reviewer #1: All comments have been addressed

Reviewer #2: (No Response)

**********

2. Is the manuscript technically sound, and do the data support the conclusions?

Reviewer #1: Partly

Reviewer #2: Yes

**********

3. Has the statistical analysis been performed appropriately and rigorously?

Reviewer #1: No

Reviewer #2: Yes

**********

4. Have the authors made all data underlying the findings in their manuscript fully available?

The PLOS Data policy

Reviewer #1: Yes

Reviewer #2: Yes

**********

5. Is the manuscript presented in an intelligible fashion and written in standard English?

Reviewer #1: Yes

Reviewer #2: Yes

**********

Reviewer #1: After examining the revised manuscript, it is clear that the authors attempted to respond to many comments; however, a substantial portion of the revisions remains explanatory rather than corrective, and the statistical reliability of the study has not been fully established. First, the clarification regarding the GNN model target variable is not methodologically sufficient because the “propensity score” is not an observed biological outcome but a composite score constructed by the researchers. Therefore, the model does not predict a real outcome but rather relearns predefined features within the same dataset. Consequently, the reported high AUC and R² values reflect internal consistency rather than genuine predictive performance, and they cannot be considered a valid validation metric. In addition, converting the continuous score into a binary classification using a threshold of 0.5 is statistically arbitrary and lacks biological or clinical justification.

Although the addition of nested cross validation is a positive step regarding data leakage concerns, it still does not provide true external validation because all data sources originate from overlapping biological knowledge bases. Presenting cBioPortal confirmation as independent validation is methodologically incorrect since it belongs to the same information universe used during model construction. Therefore, the generalizability problem remains unresolved.

The application of FDR correction is an appropriate direction; however, the statement that significance was considered at p<0.05 across analyses is contradictory because, once FDR is applied, adjusted p values must be the primary inference criterion. Adding hazard ratios and confidence intervals to survival analysis is appropriate, yet without clinical covariates the results still do not reach the level of an interpretable prognostic model.

The justification of variant classification using a seven tool consensus cites literature support but does not resolve the independence assumption problem, and without measuring inter tool agreement bias persists. Therefore, inferential reliability remains limited.

Reframing structural analyses as hypothesis generating is scientifically appropriate, but since no quantitative thresholds linking energy change to functional loss are defined, the biological interpretability of the results remains weak. Similarly, presenting k means analysis as exploratory is acceptable, yet without confirmatory statistical validation its scientific contribution remains limited.

Acknowledging circular reasoning as a limitation is positive, but because the dataset initially consists of cancer related genes, the model’s ability to generate novel information has still not been demonstrated. Accepting that identifying TP53 is expected does not eliminate this methodological limitation.

In conclusion, the revisions improved clarity and transparency but did not resolve the fundamental statistical problems. The model does not predict a true clinical or biological outcome, no independent validation exists, performance metrics remain misleading due to the derived target variable, and most interpretations remain exploratory. Therefore, the revisions are partially corrective but still insufficient methodologically, and the study can only be considered reliable after strong external validation, a true dependent variable definition, and stricter limitation of causal interpretations.

Reviewer #2: Comments

-------------

I thank the authors for the detailed revision and point-by-point response. The manuscript has clearly improved in structure, clarity, and reporting transparency. Several concerns raised in the initial review have been acknowledged and textually addressed. However, in many instances the revision resolves presentation issues rather than the underlying methodological limitations. As a result, the interpretability of the biological conclusions remains only partially improved.

1. Regarding conceptual novelty, the authors now explicitly state that the aim of the study is prioritization rather than discovery. While this clarification is helpful, it effectively confirms the original concern that the analysis primarily re-identifies well-established gastric cancer drivers such as TP53, CDH1, and APC rather than generating new biological insight. Thus, although the authors have answered the question about novelty, the revision does not fundamentally strengthen the scientific contribution beyond integrative ranking.

2. For the graph neural network framework, the authors now define the model output as a composite “propensity score” derived from expression dysregulation, network centrality, and pathway membership. This clarification improves transparency but simultaneously reveals that the model is trained to predict a constructed heuristic score based on the same input features. Consequently, the reported performance metrics represent successful reconstruction of an engineered ranking rather than predictive biological inference. The authors clarified whether the task is supervised or semi-supervised and added baseline comparisons, which addresses part of the methodological description. However, the concern about inflated performance due to feature selection and lack of biological validation remains unresolved.

3. The issue of circular reasoning has been acknowledged by the authors, who now discuss the expected identification of TP53 as a hub and provide an option to exclude known pathway genes. This represents a partial improvement in transparency, but the study still does not demonstrate discovery capability independent of prior biological knowledge. Therefore, the core concern is acknowledged but not experimentally resolved.

4. The authors appropriately revised the structural and energetic analyses by softening causal language and describing them as hypothesis-generating rather than definitive. Similarly, the atomic-level clustering analysis has been reframed as exploratory. These changes satisfactorily address the overinterpretation concern from a reporting standpoint, although the analyses remain descriptive in nature.

5. The survival analyses have been improved by including false discovery rate correction, hazard ratios, and explicit acknowledgment that associations are correlative. Nevertheless, the analyses still lack adjustment for clinical covariates such as stage or treatment, meaning the biological interpretation remains limited. Thus, the statistical reporting is improved but the inferential strength remains modest.

6. The manuscript length and redundancy have been reduced, and several secondary analyses have been moved to supplementary material, which adequately addresses the scope concern. Minor issues such as overly assertive language, terminology consistency, and code availability have also been corrected, and the inclusion of a public repository improves reproducibility. The technical questions were answered descriptively, though largely without additional quantitative validation.

Overall, the revision substantially improves clarity, organization, and transparency. However, the central methodological concerns — particularly the non-independent training objective, limited biological validation, and reliance on rediscovery of known drivers — remain only partially resolved. The manuscript now reads as a computational prioritization resource rather than a predictive or mechanistic framework, and the conclusions should be interpreted accordingly.

**********

what does this mean?). If published, this will include your full peer review and any attached files.

If you choose “no”, your identity will remain anonymous but your review may still be made public.

**Do you want your identity to be public for this peer review?**  For information about this choice, including consent withdrawal, please see our Privacy Policy

Reviewer #1: **Yes:**  Mustafa Agah TEKİNDAL

Reviewer #2: **Yes:**  Somnath Tagore

**********

[NOTE: If reviewer comments were submitted as an attachment file, they will be attached to this email and accessible via the submission site. Please log into your account, locate the manuscript record, and check for the action link "View Attachments". If this link does not appear, there are no attachment files.]

To ensure your figures meet our technical requirements, please review our figure guidelines: https://journals.plos.org/plosone/s/figures

You may also use PLOS’s free figure tool, NAAS, to help you prepare publication quality figures: https://journals.plos.org/plosone/s/figures#loc-tools-for-figure-preparation.

NAAS will assess whether your figures meet our technical requirements by comparing each figure against our figure specifications.

# Author comment: Author response to Decision Letter 2

**DOI:** 10.1371/journal.pone.0358440.r004

4 Mar 2026

For better presentation, responses are attached within the cover letter.

Attachment Submitted filename: Response_to_Reviewers_auresp_2.pdf

# Peer review: Decision Letter 2

**DOI:** 10.1371/journal.pone.0358440.r005

**Authors:** Amr Ahmed El-Arabey

27 Mar 2026

Dear Dr. Mahmud,

Thank you for submitting your manuscript to PLOS ONE. After careful consideration, we feel that it has merit but does not fully meet PLOS ONE’s publication criteria as it currently stands. Therefore, we invite you to submit a revised version of the manuscript that addresses the points raised during the review process.

Please submit your revised manuscript by May 11 2026 11:59PM. If you will need more time than this to complete your revisions, please reply to this message or contact the journal office at plosone@plos.org. When you're ready to submit your revision, log on to https://www.editorialmanager.com/pone/ and select the 'Submissions Needing Revision' folder to locate your manuscript file.

- A letter that responds to each point raised by the academic editor and reviewer(s). You should upload this letter as a separate file labeled 'Response to Reviewers'.
- A marked-up copy of your manuscript that highlights changes made to the original version. You should upload this as a separate file labeled 'Revised Manuscript with Track Changes'.
- An unmarked version of your revised paper without tracked changes. You should upload this as a separate file labeled 'Manuscript'.

If you would like to make changes to your financial disclosure, please include your updated statement in your cover letter. Guidelines for resubmitting your figure files are available below the reviewer comments at the end of this letter.

If applicable, we recommend that you deposit your laboratory protocols in protocols.io to enhance the reproducibility of your results. Protocols.io assigns your protocol its own identifier (DOI) so that it can be cited independently in the future. For instructions see: https://journals.plos.org/plosone/s/submission-guidelines#loc-laboratory-protocols. Additionally, PLOS ONE offers an option for publishing peer-reviewed Lab Protocol articles, which describe protocols hosted on protocols.io. Read more information on sharing protocols at https://plos.org/protocols?utm_medium=editorial-email&utm_source=authorletters&utm_campaign=protocols.

As the corresponding author, your ORCID iD is verified in the submission system and will appear in the published article. PLOS supports the use of ORCID, and we encourage all coauthors to register for an ORCID iD and use it as well. Please encourage your coauthors to verify their ORCID iD within the submission system before final acceptance, as unverified ORCID iDs will not appear in the published article. *Only*  the individual author can complete the verification step; PLOS staff *cannot*  verify ORCID iDs on behalf of authors.

We look forward to receiving your revised manuscript.

Kind regards,

Amr Ahmed El-Arabey

Academic Editor

PLOS One

**Journal Requirements:**

1. If the reviewer comments include a recommendation to cite specific previously published works, please review and evaluate these publications to determine whether they are relevant and should be cited. There is no requirement to cite these works unless the editor has indicated otherwise.

2. Please review your reference list to ensure that it is complete and correct. If you have cited papers that have been retracted, please include the rationale for doing so in the manuscript text, or remove these references and replace them with relevant current references. Any changes to the reference list should be mentioned in the rebuttal letter that accompanies your revised manuscript. If you need to cite a retracted article, indicate the article’s retracted status in the References list and also include a citation and full reference for the retraction notice.

[Note: HTML markup is below. Please do not edit.]

Reviewers' comments:

Reviewer's Responses to Questions

**Comments to the Author**

Reviewer #1: All comments have been addressed

Reviewer #2: All comments have been addressed

**********

2. Is the manuscript technically sound, and do the data support the conclusions?

Reviewer #1: Yes

Reviewer #2: Partly

**********

3. Has the statistical analysis been performed appropriately and rigorously?

Reviewer #1: No

Reviewer #2: Yes

**********

4. Have the authors made all data underlying the findings in their manuscript fully available?

The PLOS Data policy

Reviewer #1: Yes

Reviewer #2: Yes

**********

5. Is the manuscript presented in an intelligible fashion and written in standard English?

Reviewer #1: Yes

Reviewer #2: Yes

**********

**Reviewer #1:**  R2 revision demonstrates several improvements compared with the previous version; however, the revisions are still not fully sufficient from a methodological standpoint. While the authors have clarified several technical aspects of the modeling framework and expanded the methodological description, some of the fundamental statistical concerns remain unresolved.

One positive improvement is the clearer description of the modeling framework. The authors now explain the Graph Neural Network architecture in greater detail and specify the training, validation, and testing split of the dataset using a 60–20–20 partition. In addition, the implementation of nested 5-fold cross validation for hyperparameter tuning and model evaluation represents an appropriate strategy to reduce information leakage and obtain a more unbiased estimate of model performance. These methodological clarifications improve transparency and reproducibility. However, the primary methodological concern persists because the dependent variable used in the model remains a constructed composite “propensity score” rather than an observed biological or clinical outcome. This score is generated by combining normalized gene expression magnitude, graph-topological centrality measures, and pathway membership information. Consequently, the model effectively learns to reproduce a score that is derived from the same features used as model inputs. In such cases, high predictive performance metrics, such as the reported AUC values around 0.93 and R² values around 0.85, may reflect internal consistency rather than genuine predictive capability. Therefore, the reported performance should be interpreted cautiously because the model is not predicting an independent biological outcome but rather a derived internal metric.

Another issue concerns the conversion of the continuous propensity score into a binary classification using a threshold of 0.5 after sigmoid normalization. Although the authors describe this step as a secondary interpretive analysis, the justification for this threshold is not clearly explained. Without a biological or statistically optimized decision threshold, the binarization process appears somewhat arbitrary and may influence the ROC-based evaluation metrics. In predictive modeling studies, threshold selection typically requires either clinical reasoning or optimization methods such as Youden index or cost-sensitive decision rules.

The validation strategy also shows partial improvement but remains limited. The authors expanded the variant validation step using data from the cBioPortal database and analyzed a large cohort of gastric cancer samples across multiple studies. While this strengthens the biological context of the findings, it still cannot be considered a true external validation. The training data sources and validation data originate from the same general genomic data ecosystem, meaning that the independence between training and validation datasets may not be sufficient to demonstrate real model generalizability.

Regarding statistical inference, the authors indicate that FDR-adjusted p-values were used for identifying differentially expressed genes. This is an appropriate correction for multiple comparisons. However, the manuscript still reports statistical significance primarily using the conventional p<0.05 threshold in several sections, which creates ambiguity regarding whether adjusted p-values or nominal p-values are being interpreted. If FDR correction was applied, the adjusted p-values should consistently be reported and used as the main inferential criterion.

In the survival analysis section, Kaplan–Meier analysis is presented to assess associations between gene expression and patient survival. While this provides an initial descriptive assessment, the absence of multivariable Cox proportional hazards modeling limits the clinical interpretability of the results. Without adjusting for potential confounding variables such as tumor stage, age, or treatment factors, the survival associations remain exploratory rather than predictive.

The variant prioritization strategy also raises methodological considerations. The use of seven computational prediction tools to classify missense variants provides a consensus-based filtering strategy that may improve sensitivity; however, these tools are not statistically independent and often rely on overlapping structural and evolutionary features. As a result, agreement among tools should not be interpreted as independent validation. Ideally, inter-tool agreement statistics or external functional validation would strengthen the credibility of these predictions.

Furthermore, several analyses in the manuscript, including structural energy estimation, electrostatic analysis, and k-means clustering of atomic-level structural features, should be interpreted as exploratory computational analyses. While they provide potentially interesting mechanistic insights, they do not constitute direct biological validation. These analyses generate hypotheses regarding structural instability or functional perturbation, but the causal relationship between the predicted structural changes and gastric cancer pathogenesis remains inferential.

In summary, the R2 revision improves the clarity of the methodology and addresses several technical concerns raised in the previous review. The description of the neural network architecture, cross-validation framework, and computational workflow is now considerably more transparent. Nevertheless, the central statistical limitations remain partially unresolved. The model still relies on a derived dependent variable rather than an independent biological outcome, the classification threshold lacks clear justification, true external validation is absent, and several inferential conclusions remain exploratory. Therefore, while the revisions represent meaningful progress and partially address previous concerns, they are not entirely sufficient from a strict methodological and statistical perspective. Additional external validation, clearer justification of modeling choices, and stronger statistical inference would substantially strengthen the scientific reliability of the study.

**Reviewer #2:**  The manuscript has improved substantially in clarity, organization, and reporting transparency after revision. The authors have addressed many of the concerns raised in prior review rounds, especially with respect to manuscript structure, clearer method descriptions, inclusion of code availability, tempering of causal language, and better explanation of the graph neural network framework. While some methodological limitations remain, they are now more openly acknowledged, and the study is more appropriately framed as a computational prioritization resource. Given that this is the second round of review, I would support acceptance, while still noting the following comments for the authors and editor.

Major comments

--------------------

1. The revised manuscript is clearer in defining the GNN objective, and the authors now explain that the model predicts a composite propensity score integrating expression dysregulation, network centrality, and pathway membership. This improves transparency. However, the model still appears to predict a score constructed from features closely related to the same inputs, so the framework should be interpreted primarily as a **prioritization model** rather than as an independently predictive biological model.

2. The manuscript now more explicitly acknowledges that the study is focused on **prioritization rather than novel discovery**, which is an appropriate clarification. This is important because the principal highlighted genes, particularly **TP53, CDH1, and APC**, are already well-established in gastric cancer. The revised framing is more accurate, but the conclusions should continue to avoid implying that the framework uncovers fundamentally new driver biology.

3. The added explanation of nested cross-validation and gene-level partitioning strengthens the description of the validation framework. However, the integrated use of partially overlapping resources such as GEPIA2, STRING, cBioPortal, and KEGG still means that the reported performance should be interpreted with caution. The manuscript is improved on this point, but the results remain more supportive of **internal ranking robustness** than of broad biological generalizability.

4. The baseline comparisons and clarification of supervised versus semi-supervised elements improve the methodological presentation. Still, the biological interpretation of the “predicted score” versus “importance score” remains somewhat abstract. The current revision is acceptable, but the manuscript should continue to emphasize that these scores are **computational prioritization measures** and not direct biological effect estimates.

Overall, the revised paper is stronger as a computational resource and integrative prioritization study than as a mechanistic or predictive framework. I think the manuscript is now acceptable in that context, but the interpretation should remain aligned with that level of evidence.

Minor comments

------------------

1. The writing is improved, but a final language edit would still help in a few places to correct grammar, article usage, and sentence flow.

2. Some sections remain long and slightly repetitive, especially where multiple computational tools converge on the same conclusion. A final pass for conciseness would improve readability further.

3. Figure legends have improved, but a few dense figures would still benefit from even more explicit labeling of axes, color scales, and panel-specific interpretation.

4. Terminology should remain fully consistent throughout the manuscript, especially for terms such as “variant,” “missense SNP,” “oncogenic,” “tumor suppressor,” “importance score,” and “predicted score.”

5. The manuscript now includes code availability, which is a major strength. The final version should ensure that the repository link, software versions, seeds, and any required input formatting are easy for readers to follow.

**********

what does this mean?). If published, this will include your full peer review and any attached files.

If you choose “no”, your identity will remain anonymous but your review may still be made public.

**Do you want your identity to be public for this peer review?**  For information about this choice, including consent withdrawal, please see our Privacy Policy

Reviewer #1: No

Reviewer #2: **Yes:**  Somnath Tagore

**********

[NOTE: If reviewer comments were submitted as an attachment file, they will be attached to this email and accessible via the submission site. Please log into your account, locate the manuscript record, and check for the action link "View Attachments". If this link does not appear, there are no attachment files.]

To ensure your figures meet our technical requirements, please review our figure guidelines: https://journals.plos.org/plosone/s/figures

You may also use PLOS’s free figure tool, NAAS, to help you prepare publication quality figures: https://journals.plos.org/plosone/s/figures#loc-tools-for-figure-preparation.

NAAS will assess whether your figures meet our technical requirements by comparing each figure against our figure specifications.

# Author comment: Author response to Decision Letter 3

**DOI:** 10.1371/journal.pone.0358440.r006

6 Apr 2026

For better presentation, the responses were provided with the cover letter.

Attachment Submitted filename: Response to Reviewers.docx

# Peer review: Decision Letter 3

**DOI:** 10.1371/journal.pone.0358440.r007

**Authors:** Amr Ahmed El-Arabey

7 Jun 2026

Dear Dr. Mahmud,

Thank you for submitting your manuscript to PLOS ONE. After careful consideration, we feel that it has merit but does not fully meet PLOS ONE’s publication criteria as it currently stands. Therefore, we invite you to submit a revised version of the manuscript that addresses the points raised during the review process.

Please submit your revised manuscript by Jul 22 2026 11:59PM. If you will need more time than this to complete your revisions, please reply to this message or contact the journal office at plosone@plos.org. When you're ready to submit your revision, log on to https://www.editorialmanager.com/pone/ and select the 'Submissions Needing Revision' folder to locate your manuscript file.

- A letter that responds to each point raised by the academic editor and reviewer(s). You should upload this letter as a separate file labeled 'Response to Reviewers'.
- A marked-up copy of your manuscript that highlights changes made to the original version. You should upload this as a separate file labeled 'Revised Manuscript with Track Changes'.
- An unmarked version of your revised paper without tracked changes. You should upload this as a separate file labeled 'Manuscript'.

If you would like to make changes to your financial disclosure, please include your updated statement in your cover letter. Guidelines for resubmitting your figure files are available below the reviewer comments at the end of this letter.

If applicable, we recommend that you deposit your laboratory protocols in protocols.io to enhance the reproducibility of your results. Protocols.io assigns your protocol its own identifier (DOI) so that it can be cited independently in the future. For instructions see: https://journals.plos.org/plosone/s/submission-guidelines#loc-laboratory-protocols. Additionally, PLOS ONE offers an option for publishing peer-reviewed Lab Protocol articles, which describe protocols hosted on protocols.io. Read more information on sharing protocols at https://plos.org/protocols?utm_medium=editorial-email&utm_source=authorletters&utm_campaign=protocols.

As the corresponding author, your ORCID iD is verified in the submission system and will appear in the published article. PLOS supports the use of ORCID, and we encourage all coauthors to register for an ORCID iD and use it as well. Please encourage your coauthors to verify their ORCID iD within the submission system before final acceptance, as unverified ORCID iDs will not appear in the published article. *Only*  the individual author can complete the verification step; PLOS staff *cannot*  verify ORCID iDs on behalf of authors.

We look forward to receiving your revised manuscript.

Kind regards,

Amr Ahmed El-Arabey

Academic Editor

PLOS One

Journal Requirements:

If the reviewer comments include a recommendation to cite specific previously published works, please review and evaluate these publications to determine whether they are relevant and should be cited. There is no requirement to cite these works unless the editor has indicated otherwise.

Please review your reference list to ensure that it is complete and correct. If you have cited papers that have been retracted, please include the rationale for doing so in the manuscript text, or remove these references and replace them with relevant current references. Any changes to the reference list should be mentioned in the rebuttal letter that accompanies your revised manuscript. If you need to cite a retracted article, indicate the article’s retracted status in the References list and also include a citation and full reference for the retraction notice.

[Note: HTML markup is below. Please do not edit.]

Reviewers' comments:

Reviewer's Responses to Questions

**Comments to the Author**

Reviewer #1: All comments have been addressed

**********

2. Is the manuscript technically sound, and do the data support the conclusions?

Reviewer #1: Partly

**********

3. Has the statistical analysis been performed appropriately and rigorously?

Reviewer #1: Yes

**********

4. Have the authors made all data underlying the findings in their manuscript fully available?

The PLOS Data policy

Reviewer #1: Yes

**********

5. Is the manuscript presented in an intelligible fashion and written in standard English?

Reviewer #1: Yes

**********

Reviewer #1: The authors have substantially improved the manuscript compared with the previous version. The clarification of the graph neural network framework, inclusion of nested cross-validation procedures, additional details regarding data partitioning strategies to minimize information leakage, and improved reporting of model performance metrics have strengthened the methodological transparency of the study. The computational workflow is now considerably clearer and the overall presentation has improved substantially.

Nevertheless, several minor issues remain that should be addressed before publication. First, although the authors have clarified the construction of the composite propensity score used as the target variable, a brief discussion acknowledging the potential circularity associated with predicting a score derived from network and expression features would improve transparency. Second, the rationale for selecting the 0.5 threshold used for binarization of propensity scores should be explained more explicitly, even if only as a practical prioritization criterion. Third, while the use of multiple pathogenicity prediction tools increases confidence in variant prioritization, the limitations arising from potential dependencies among these tools should be acknowledged in the discussion section. Finally, the survival analyses would benefit from reporting effect size estimates whenever available and from a brief discussion regarding the limitations of unadjusted survival analyses based on publicly available datasets.

These points do not require additional experiments or major reanalysis and can be addressed through clarification and expanded discussion. Therefore, I believe the manuscript is suitable for publication after minor revision.

**********

what does this mean?). If published, this will include your full peer review and any attached files.

If you choose “no”, your identity will remain anonymous but your review may still be made public.

**Do you want your identity to be public for this peer review?**  For information about this choice, including consent withdrawal, please see our Privacy Policy

Reviewer #1: No

**********

[NOTE: If reviewer comments were submitted as an attachment file, they will be attached to this email and accessible via the submission site. Please log into your account, locate the manuscript record, and check for the action link "View Attachments". If this link does not appear, there are no attachment files.]

To ensure your figures meet our technical requirements, please review our figure guidelines: https://journals.plos.org/plosone/s/figures

You may also use PLOS’s free figure tool, NAAS, to help you prepare publication quality figures: https://journals.plos.org/plosone/s/figures#loc-tools-for-figure-preparation.

NAAS will assess whether your figures meet our technical requirements by comparing each figure against our figure specifications.

# Author comment: Author response to Decision Letter 4

**DOI:** 10.1371/journal.pone.0358440.r008

18 Jun 2026

Dear Editor,

Greetings.

For better understanding the 'response to reviewer' has been included just after cover letter.

Thank you.

Attachment Submitted filename: Review response.docx

# Peer review: Decision Letter 4

**DOI:** 10.1371/journal.pone.0358440.r009

**Authors:** Amr Ahmed El-Arabey

2 Jul 2026

Dear Dr. Mahmud,

Thank you for submitting your manuscript to PLOS ONE. After careful consideration, we feel that it has merit but does not fully meet PLOS ONE’s publication criteria as it currently stands. Therefore, we invite you to submit a revised version of the manuscript that addresses the points raised during the review process.

Please submit your revised manuscript by Aug 16 2026 11:59PM. If you will need more time than this to complete your revisions, please reply to this message or contact the journal office at plosone@plos.org. When you're ready to submit your revision, log on to https://www.editorialmanager.com/pone/ and select the 'Submissions Needing Revision' folder to locate your manuscript file.

- A letter that responds to each point raised by the academic editor and reviewer(s). You should upload this letter as a separate file labeled 'Response to Reviewers'.
- A marked-up copy of your manuscript that highlights changes made to the original version. You should upload this as a separate file labeled 'Revised Manuscript with Track Changes'.
- An unmarked version of your revised paper without tracked changes. You should upload this as a separate file labeled 'Manuscript'.

If you would like to make changes to your financial disclosure, please include your updated statement in your cover letter. Guidelines for resubmitting your figure files are available below the reviewer comments at the end of this letter.

If applicable, we recommend that you deposit your laboratory protocols in protocols.io to enhance the reproducibility of your results. Protocols.io assigns your protocol its own identifier (DOI) so that it can be cited independently in the future. For instructions see: https://journals.plos.org/plosone/s/submission-guidelines#loc-laboratory-protocols. Additionally, PLOS ONE offers an option for publishing peer-reviewed Lab Protocol articles, which describe protocols hosted on protocols.io. Read more information on sharing protocols at https://plos.org/protocols?utm_medium=editorial-email&utm_source=authorletters&utm_campaign=protocols.

As the corresponding author, your ORCID iD is verified in the submission system and will appear in the published article. PLOS supports the use of ORCID, and we encourage all coauthors to register for an ORCID iD and use it as well. Please encourage your coauthors to verify their ORCID iD within the submission system before final acceptance, as unverified ORCID iDs will not appear in the published article. *Only*  the individual author can complete the verification step; PLOS staff *cannot*  verify ORCID iDs on behalf of authors.

We look forward to receiving your revised manuscript.

Kind regards,

Amr Ahmed El-Arabey

Academic Editor

PLOS One

Journal Requirements:

1. If the reviewer comments include a recommendation to cite specific previously published works, please review and evaluate these publications to determine whether they are relevant and should be cited. There is no requirement to cite these works unless the editor has indicated otherwise.

2. Please review your reference list to ensure that it is complete and correct. If you have cited papers that have been retracted, please include the rationale for doing so in the manuscript text, or remove these references and replace them with relevant current references. Any changes to the reference list should be mentioned in the rebuttal letter that accompanies your revised manuscript. If you need to cite a retracted article, indicate the article’s retracted status in the References list and also include a citation and full reference for the retraction notice.

[Note: HTML markup is below. Please do not edit.]

Reviewers' comments:

Reviewer's Responses to Questions

**Comments to the Author**

Reviewer #1: All comments have been addressed

**********

2. Is the manuscript technically sound, and do the data support the conclusions?

Reviewer #1: Partly

**********

3. Has the statistical analysis been performed appropriately and rigorously?

Reviewer #1: Yes

**********

4. Have the authors made all data underlying the findings in their manuscript fully available?

The PLOS Data policy

Reviewer #1: Yes

**********

5. Is the manuscript presented in an intelligible fashion and written in standard English?

Reviewer #1: Yes

**********

Reviewer #1: The authors have made a substantial effort to address the concerns raised during the previous rounds of review. Compared with the earlier versions, the manuscript has been significantly strengthened, particularly with respect to the transparency of the computational workflow and the methodological description. The Graph Neural Network architecture is now described in considerably greater detail, the training, validation, and testing strategy is clearly presented, nested cross-validation has been implemented, and additional information regarding data partitioning and measures to reduce information leakage has been incorporated. The statistical methodology is now more transparent and reproducible than in the previous submissions. Furthermore, the authors have clarified the distinction between the primary regression framework and the secondary classification analysis, expanded the justification for the computational prediction tools employed, improved the description of multiple testing correction, and substantially enhanced the Discussion by acknowledging several methodological limitations. The data availability statement has also been revised in accordance with the journal's requirements. Overall, the manuscript has undergone considerable methodological improvement, and the majority of the previous statistical concerns have been satisfactorily addressed.

One methodological issue, however, still deserves a clearer acknowledgment rather than additional analyses. The Graph Neural Network is trained to predict a researcher-derived composite propensity score that integrates gene expression, network topology, and pathway information rather than an independent biological or clinical outcome. Consequently, the reported predictive performance metrics should be interpreted as measures of the model's ability to prioritize biologically relevant candidate genes within the constructed framework rather than as evidence of true clinical prediction or external prognostic performance. Although the authors have substantially clarified this modeling strategy, explicitly emphasizing this distinction in the Discussion and Conclusions would prevent potential overinterpretation of the reported predictive accuracy and further improve the scientific rigor of the manuscript.

Overall, I believe the authors have responded carefully and constructively to the reviewers' comments, and the manuscript has improved substantially during the revision process. The remaining concern is primarily one of interpretation rather than methodology and can be adequately addressed through a minor textual clarification. Therefore, I consider the manuscript suitable for publication after minor revision.

**********

what does this mean?). If published, this will include your full peer review and any attached files.

If you choose “no”, your identity will remain anonymous but your review may still be made public.

**Do you want your identity to be public for this peer review?**  For information about this choice, including consent withdrawal, please see our Privacy Policy

Reviewer #1: **Yes:**  Mustafa Agah TEKİNDAL

**********

[NOTE: If reviewer comments were submitted as an attachment file, they will be attached to this email and accessible via the submission site. Please log into your account, locate the manuscript record, and check for the action link "View Attachments". If this link does not appear, there are no attachment files.]

To ensure your figures meet our technical requirements, please review our figure guidelines: https://journals.plos.org/plosone/s/figures

You may also use PLOS’s free figure tool, NAAS, to help you prepare publication quality figures: https://journals.plos.org/plosone/s/figures#loc-tools-for-figure-preparation.

NAAS will assess whether your figures meet our technical requirements by comparing each figure against our figure specifications.

# Author comment: Author response to Decision Letter 5

**DOI:** 10.1371/journal.pone.0358440.r010

5 Jul 2026

For better presentation, response to reviewer has been attached just after the cover letter.

Attachment Submitted filename: Response to Reviewer (GC).docx

# Peer review: Decision Letter 5

**DOI:** 10.1371/journal.pone.0358440.r011

**Authors:** Mohammed Razzaque

2 Sep 2026

Computational screening of oncogenic genetic variations in tumor suppressor proteins driving gastric cancer pathogenesis

PONE-D-25-52287R5

Dear Dr. Mahmud,

We’re pleased to inform you that your manuscript has been judged scientifically suitable for publication and will be formally accepted for publication once it meets all outstanding technical requirements.

Within one week, you’ll receive an e-mail detailing the required amendments. When these have been addressed, you’ll receive a formal acceptance letter and your manuscript will be scheduled for publication.

An invoice will be generated when your article is formally accepted. Please note, if your institution has a publishing partnership with PLOS and your article meets the relevant criteria, all or part of your publication costs will be covered. Please make sure your user information is up-to-date by logging into Editorial Manager at Editorial Manager® and clicking the ‘Update My Information' link at the top of the page. For questions related to billing, please contact billing support.

If your institution or institutions have a press office, please notify them about your upcoming paper to help maximize its impact. If they’ll be preparing press materials, please inform our press team as soon as possible -- no later than 48 hours after receiving the formal acceptance. Your manuscript will remain under strict press embargo until 2 pm Eastern Time on the date of publication. For more information, please contact onepress@plos.org.

Kind regards,

Mohammed S. Razzaque, MBBS, PhD

Academic Editor

PLOS One

Additional Editor Comments (optional):

Reviewers' comments:

Reviewer's Responses to Questions

**Comments to the Author**

Reviewer #1: All comments have been addressed

**********

2. Is the manuscript technically sound, and do the data support the conclusions?

Reviewer #1: Partly

**********

3. Has the statistical analysis been performed appropriately and rigorously?

Reviewer #1: Yes

**********

4. Have the authors made all data underlying the findings in their manuscript fully available?

The PLOS Data policy

Reviewer #1: (No Response)

**********

5. Is the manuscript presented in an intelligible fashion and written in standard English?

Reviewer #1: Yes

**********

Reviewer #1: (No Response)

**********

what does this mean?). If published, this will include your full peer review and any attached files.

If you choose “no”, your identity will remain anonymous but your review may still be made public.

**Do you want your identity to be public for this peer review?**  For information about this choice, including consent withdrawal, please see our Privacy Policy

Reviewer #1: No

**********

# Editor report: Acceptance letter

**DOI:** 10.1371/journal.pone.0358440.r012

**Authors:** Mohammed Razzaque

PONE-D-25-52287R5

PLOS One

Dear Dr. Mahmud,

I'm pleased to inform you that your manuscript has been deemed suitable for publication in PLOS One. Congratulations! Your manuscript is now being handed over to our production team.

At this stage, our production department will prepare your paper for publication. This includes ensuring the following:

* All references, tables, and figures are properly cited

* All relevant supporting information is included in the manuscript submission,

* There are no issues that prevent the paper from being properly typeset

You will receive further instructions from the production team, including instructions on how to review your proof when it is ready. Please keep in mind that we are working through a large volume of accepted articles, so please give us a few days to review your paper and let you know the next and final steps.

Lastly, if your institution or institutions have a press office, please let them know about your upcoming paper now to help maximize its impact. If they'll be preparing press materials, please inform our press team within the next 48 hours. Your manuscript will remain under strict press embargo until 2 pm Eastern Time on the date of publication. For more information, please contact onepress@plos.org.

You will receive an invoice from PLOS for your publication fee after your manuscript has reached the completed accept phase. If you receive an email requesting payment before acceptance or for any other service, this may be a phishing scheme. Learn how to identify phishing emails and protect your accounts at https://explore.plos.org/phishing.

If we can help with anything else, please email us at customercare@plos.org.

Thank you for submitting your work to PLOS One and supporting open access.

Kind regards,

PLOS One Editorial Office Staff

on behalf of

Dr. Mohammed S. Razzaque

Academic Editor

PLOS One
